# clusterExperiment and RSEC: A Bioconductor package and framework for clustering of single-cell and other large gene expression datasets

**DOI:** 10.1371/journal.pcbi.1006378

**Published:** 2018-09-04

**Authors:** Davide Risso, Liam Purvis, Russell B. Fletcher, Diya Das, John Ngai, Sandrine Dudoit, Elizabeth Purdom

**Affiliations:** 1 Division of Biostatistics and Epidemiology, Weill Cornell Medicine, New York, New York, United States of America; 2 Department of Statistics, University of California - Berkeley, Berkeley, California, United States of America; 3 Department of Molecular and Cell Biology, University of California - Berkeley, Berkeley, California, United States of America; 4 Berkeley Institute for Data Science, UC University of California - Berkeley, Berkeley, California, United States of America; 5 Division of Epidemiology and Biostatistics, University of California - Berkeley, Berkeley, California, United States of America; University of Technology Sydney, AUSTRALIA

## Abstract

Clustering of genes and/or samples is a common task in gene expression analysis. The goals in clustering can vary, but an important scenario is that of finding biologically meaningful subtypes within the samples. This is an application that is particularly appropriate when there are large numbers of samples, as in many human disease studies. With the increasing popularity of single-cell transcriptome sequencing (RNA-Seq), many more controlled experiments on model organisms are similarly creating large gene expression datasets with the goal of detecting previously unknown heterogeneity within cells. It is common in the detection of novel subtypes to run many clustering algorithms, as well as rely on subsampling and ensemble methods to improve robustness. We introduce a Bioconductor R package, clusterExperiment, that implements a general and flexible strategy we entitle Resampling-based Sequential Ensemble Clustering (RSEC). RSEC enables the user to easily create multiple, competing clusterings of the data based on different techniques and associated tuning parameters, including easy integration of resampling and sequential clustering, and then provides methods for consolidating the multiple clusterings into a final consensus clustering. The package is modular and allows the user to separately apply the individual components of the RSEC procedure, i.e., apply multiple clustering algorithms, create a consensus clustering or choose tuning parameters, and merge clusters. Additionally, clusterExperiment provides a variety of visualization tools for the clustering process, as well as methods for the identification of possible cluster signatures or biomarkers. The R package clusterExperiment is publicly available through the Bioconductor Project, with a detailed manual (vignette) as well as well documented help pages for each function.

This is a *PLOS Computational Biology* Software paper.

## Introduction

The clustering of samples or genes is one of the most common tasks in gene expression studies and, in many studies with large numbers of samples, the precise allocation of samples to clusters is critical to identify biological subtypes. With single-cell transcriptome sequencing (scRNA-Seq) studies, in particular, the boundaries between subtypes can be quite fuzzy, with individual cells lying on cluster boundaries. Similarly, outlying contaminant cells are also common. These problems highlight the need for robust identification of clusters, and several clustering algorithms have been recently proposed for the specific task of clustering single-cell sequencing data [1–7].

We introduce here the Bioconductor R package clusterExperiment, which implements not a specific clustering algorithm, but a general and flexible framework in particular useful for the clustering of cells based on single-cell RNA-Seq data. We also introduce a specific clustering workflow, entitled Resampling-based Sequential Ensemble Clustering (RSEC). It enables researchers to easily try a variety of different clustering algorithms and associated tuning parameters and generate a stable consensus clustering from these many candidate clusterings. Specifically, given user-supplied base clustering algorithms and associated tuning parameters, RSEC runs the algorithms and generates the corresponding collection of candidate clusterings, with the option of resampling cells and of using a sequential clustering procedure as in [8]. As in supervised learning, resampling can improve the stability of clusters [9–14] and has been frequently suggested in gene expression clustering [15–19] and more recently for single-cell studies specifically [1]. Additionally, considering an ensemble of methods and tuning parameters allows the user to capitalize on the different strengths of the base algorithms and avoid the subjective selection of tuning parameters. RSEC provides a strategy for defining a consensus clustering from the many candidate clusterings and a method of further merging similar clusters that do not show strong individual gene expression differences.

Unlike many existing clustering software for single-cell sequencing and gene expression data, clusterExperiment provides a flexible framework that allows for user customization of the clustering algorithm and accompanying manipulation of the data. Finally, the clusterExperiment package is fully integrated into the Bioconductor software suite, inheriting from the existing SingleCellExperiment class (a baseline class for storing single-cell data) [20], and interfaces with common differential expression (DE) packages like limma [21], MAST [22], and edgeR [23] to find marker genes for the clusters.

## Design and implementation

In what follows, we define a “clustering” as the set of clusters found by a single run of a clustering method, while a “cluster” refers to a set of samples within a clustering.

### RSEC workflow

The clusterExperiment package provides a novel workflow for creating a unified clustering from many clustering results, which we entitle Resampling-based Sequential Ensemble Clustering (RSEC). RSEC formalizes many choices that are often seen in practice when clustering large RNA-Seq expression datasets. In particular, RSEC formalizes the process of manually experimenting with many different parameter choices by systematically running them all and then provides a formal mechanism for creating an ensemble or consensus clustering from the results.

**Fig 1:**
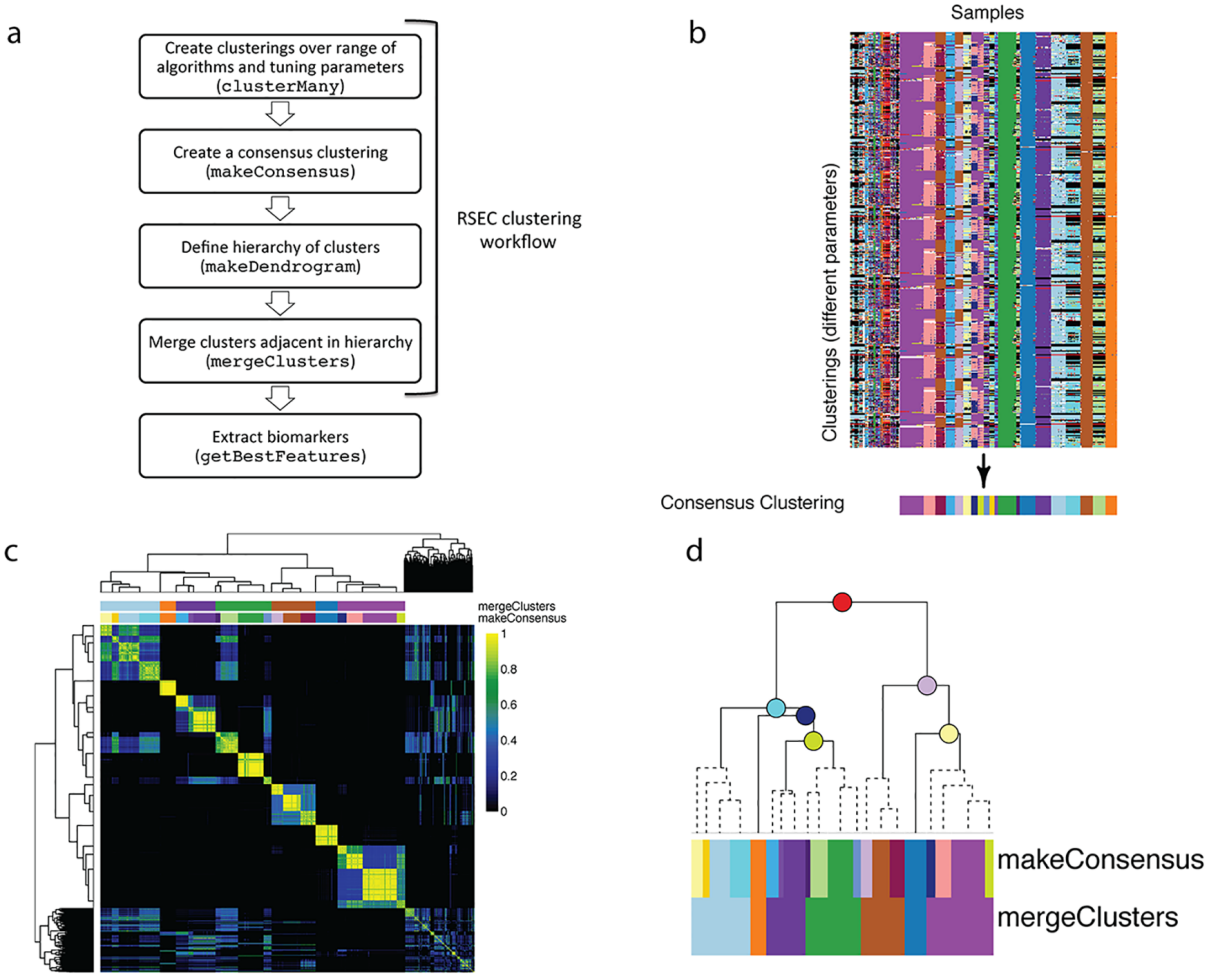
Main steps of RSEC workflow. (a) shows a diagram of the steps to the workflow while (b)-(d) demonstrate these steps on the olfactory epithelium dataset. (b) The clusterMany step produces many clusterings from the different combinations of algorithms and tuning parameters. These clusterings are displayed using the plotClusters function. Each column of the plot corresponds to a sample and each row to a clustering from the clusterMany step. The samples in each row are color-coded by their cluster assignment in that clustering; samples that are not assigned to a cluster are left white. The colors across different clusterings (rows) are assigned so as to have similar colors for clusters with similar samples across clusterings. The consensus clustering obtained from the makeConsensus step is also shown below the individual clusterings. (c) The makeConsensus step finds a consensus clustering across the clusterMany clusterings based on the co-occurrence of samples in these clusterings. The heatmap of the matrix of co-occurrence proportions is plotted using the plotCoClustering function. The resulting cluster assignments from makeConsensus are color-coded above the matrix, as are the assignments from the next step, mergeClusters. (d) The makeDendrogram step creates a hierarchy between the consensus clusters and then similar clusters in sister nodes are merged with mergeClusters. Plotted here with the function plotDendrogram is the hierarchy of the clusters from makeDendrogram, with merged nodes indicated with dashed lines. The makeConsensus clusters and resulting mergeClusters clusters are indicated as color-coded blocks below the dendrogram, sized according to the number of samples in each cluster. The estimated proportions of DE genes of each node are shown in S1 Fig.

The RSEC workflow comprises the following steps, demonstrated in Fig 1:

1. clusterMany Implementation of one or more clustering methods across a wide range of tuning parameter and data dimensionality choices.
2. makeConsensus Determination of a single consensus clustering from these many candidate clusterings.
3. Merging of individual clusters from the consensus clustering, involving two steps:
  - makeDendrogram Defining a hierarchical clustering of the clusters;
  - mergeClusters Merging clusters along that hierarchy by collapsing into a single cluster sister nodes between which only a small proportion of genes show differential expression.

The main RSEC wrapper function in the clusterExperiment package is our recommended implementation of this workflow. In addition to performing all workflow steps within a single function, it implements our preferred choice of subsampling and sequential clustering in the clusterMany step. It is our experience that these choices are particularly relevant for single-cell sequencing and other large RNA-Seq experiments. However, the user has the option to perform the individual steps separately using a sequence of function calls and make more customized choices within this workflow. The intermediate clusterings found at all of these steps are retained using a dedicated class for RSEC clustering results (ClusterExperiment), so that the user can examine the impact of each step using the visualization tools of the clusterExperiment package.

### Clustering procedures

We briefly describe here the core procedures that make up the RSEC workflow. More details can be found in S1 Text.

#### Generating many candidate clusterings (clusterMany)

The function clusterMany allows the user to easily select a range of clustering algorithms and tuning parameters and create different clusterings for each combination thereof. The option is given to parallelize computations across multiple cores. A few examples of parameters that can be compared are: the dimensionality reduction method, the number of dimensions, the number of clusters *K* (if appropriate for the clustering method), whether to subsample to create the clustering, and whether to sequentially detect clusters.

The choices regarding whether to subsample the data and whether to sequentially detect clusters can be paired with any clustering algorithm, but their application can be non-trivial, which makes the implementation of these procedures, independent of a particular clustering method, particularly useful.

The subsampling option in clusterExperiment generates clusterings based on randomly sampled subsets of the full set of *n* observations, and each resampled dataset is clustered with the baseline clustering algorithm. Subsampling defines a dissimilarity matrix between samples, with entries *D*_*ij*_ = 1 − *p*_*ij*_, where *p*_*ij*_ is defined as the proportion of times the pair of samples *i* and *j* were in the same cluster across all of the resampled datasets (see section Subsampling in S1 Text for details of the implementation). Subsampling does not itself define a clustering of the samples; RSEC uses the dissimilarity matrix *D* to cluster the samples. The clustering algorithm applied to *D* does not need to be that which was used on the resampled datasets. Furthermore, because *D* is a dissimilarity matrix, with entries on a well defined scale of 0–1, it is intuitive to cluster *D* so as to constrain the level of between-sample dissimilarity within clusters [8], rather than set a particular *K* for the number of clusters, a feature which we also allow.

Sequential clustering refers to the iterative detection and removal of single clusters and relies on a base clustering algorithm, like subsampling, which is iteratively re-applied after each removal of a cluster. For each clustering iteration, the sequential algorithm requires a method for specifying which is the “best” cluster so that it can be removed and the iteration continued. Our implementation of the sequential detection of clusters follows that of the tight clustering algorithm [8], but we have generalized it to fit arbitrary clustering techniques for which the user specifies the number of clusters *K*. Specifically, the “best” cluster is chosen by [8] to be that cluster which varies the least in its membership as the parameter *K* for the number of clusters is increased, as measured by the maximal percentage overlap of clusters from clusterings from *K* and *K* + 1 (the ratio of cardinality of intersection to cardinality of union).

The sequential algorithm can be particularly helpful when there is an outlying cluster that is widely different from others. Removing this cluster and re-clustering can minimize its effect on the global clustering results. Similarly, not clustering all samples can be beneficial in finding homogeneous clusters if the data are noisy or there are many samples on the boundary between clusters. (See also the clustering algorithm pcaReduce proposed by [2] for single-cell data, which adopts a sequential discovery of clusters, but is more narrowly based on reducing the number of dimensions for principal component analysis).

#### Creating a consensus clustering (makeConsensus)

After running clusterMany, the resulting ClusterExperiment object contains many clusterings and the next step is to find a single clustering that represents the commonality across the many clusterings. The function makeConsensus does this by creating a dissimilarity matrix between samples, defined by entries *D*_*ij*_ = 1 − *p*_*ij*_, where *p*_*ij*_ is the proportion of clusterings for which the pair of samples *i* and *j* are in the same cluster (Fig 1c). This dissimilarity matrix is similar to that of subsampling and is likewise clustered to create a consensus clustering.

#### Merging clusters based on a cluster hierarchy (mergeClusters)

The strategy of finding a consensus clustering used by makeConsensus emphasizes shared assignments across clusterings and can result in many small clusters. The number of final clusters can be adjusted in earlier steps in the clustering process, but it is more intuitive in practice to visualize clustering results (e.g., using heatmaps) to see which clusters have clear differences in the expression of individual genes.

Since these individual gene effects are of great interest to practitioners and are used to evaluate the quality of a cluster, we formalize this practice by systematically evaluating the estimated number of genes with large effects between the clusters and using this as a metric to merge together clusters. We do not compare all pairs of clusters, but instead hierarchically order the clusters from makeConsensus via hierarchical clustering on the median value of each gene in each cluster. For each node in the resulting dendrogram, their children nodes define two sets of samples that are candidates for being merged into a single cluster, and each gene is individually tested for differential expression between the samples in the two sets (Fig 1d). Using these individual gene results, mergeClusters calculates an estimate of the proportion of differentially expressed genes at each node comparison (with multiple published methods for doing so available to the user [24–28]). These estimates provide the basis for whether to merge clusters, working from the leaves (clusters) upward. Some of these estimates can also be optionally adjusted to require a minimal amount of log fold-change difference between the groups for a gene to be considered differentially expressed (see section mergeClusters in S1 Text).

We note that, in addition to merging clusters, the hierarchy between clusters is convenient for visualization of the clusters, as we discuss below (see the section “Visualization” below).

### Biomarker detection

A common task in clustering of gene expression datasets is to identify biomarkers, i.e., genes that strongly differentiate the clusters. Differential expression techniques involving hypothesis testing are often used for these tasks [21–23, 29], though it should be emphasized that such tools must be used merely for exploratory purposes, since there is severe overfitting when the groups being compared have been found by clustering on the same data.

**Fig 2:**
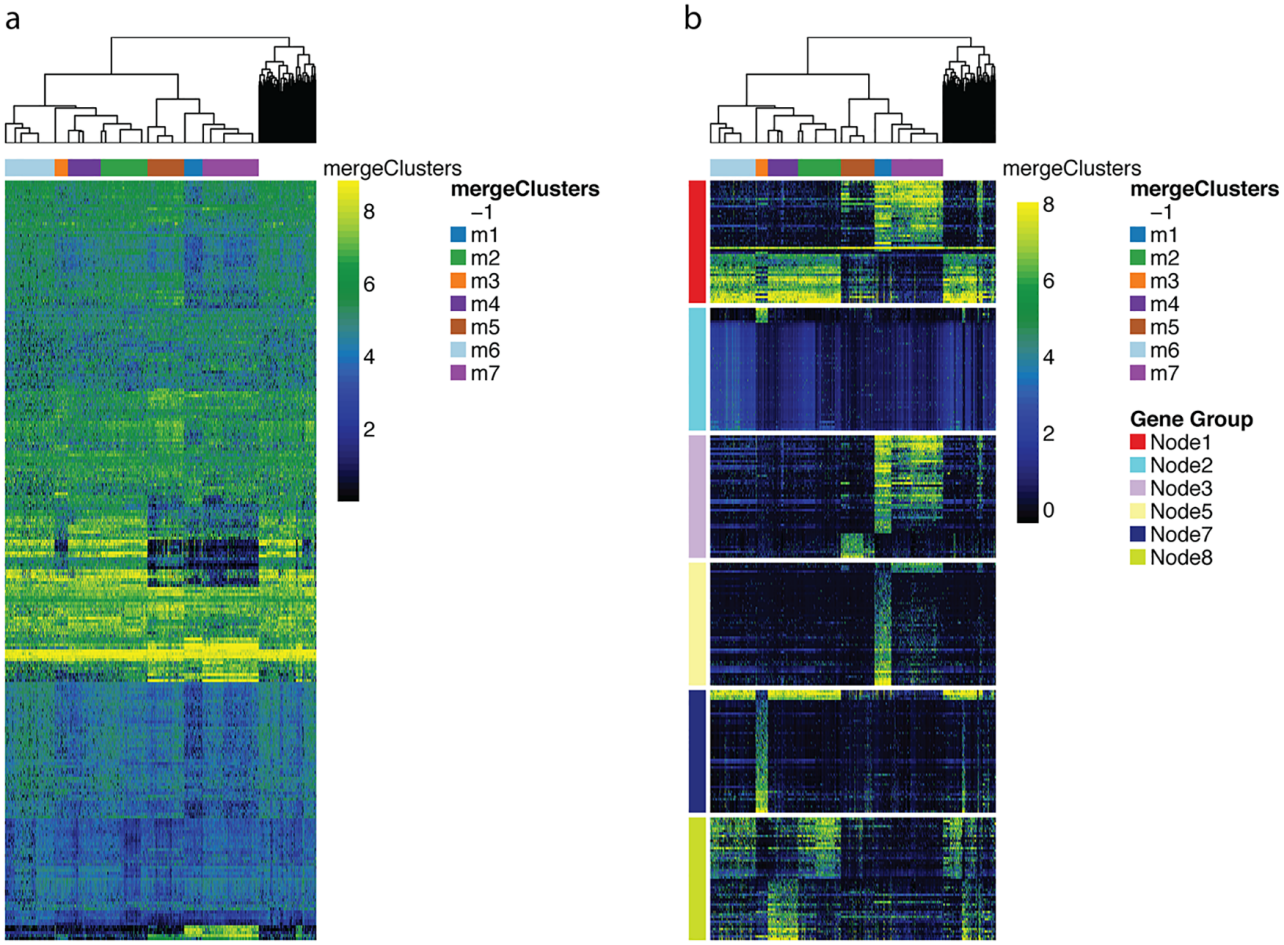
Biomarker detection, demonstrated on the olfactory epithelium dataset. Heatmap from the plotHeatmap function showing genes found differentially expressed (DE) between clusters by the function getBestFeatures, using both the global *F*-statistic (a) and hierarchical contrasts (b) options. Each of the contrasts in (b) corresponds to nodes in the dendrogram color-coded as in Fig 1d; we retained only the top 50 DE genes per node. Genes found DE in multiple contrasts may be plotted multiple times. For comparison purposes, in (a), we retained the top 256 DE genes according to a global *F*-statistic, where 256 is the number of unique genes in the hierarchical contrasts shown in (b).

Since clustering of large gene expression datasets, such as single-cell RNA-Seq datasets, generally results in a large number of clusters, finding biomarkers for the clusters corresponds to testing for differential expression between many groups. A standard *F*-statistic from an ANOVA analysis is commonly used to assess differences between the groups. However, generally vast numbers of genes will have “significant” *F*-statistics and the largest *F*-statistics can easily be dominated by genes that differentiate the single, most outlying cluster (Fig 2a). A better approach is to test for specific differences between groups, e.g., pairwise differences between two groups, by forming contrasts from the full ANOVA model, which most DE packages allow. This has the added advantage of using all of the data for estimation of the variance parameters regardless of the size of the clusters.

However, a large number of clusters will imply a large set of contrasts (for example, all pairwise contrasts). Such a large number of contrasts are practically quite difficult to construct with the common DE packages. The clusterExperiment package provides tools to create relevant contrasts and optionally returns the relevant biomarkers via analysis by limma [21], limma with voom weights [30], edgeR [23], or edgeR with weights to correct for zero-inflation [31]. We also provide the ability to port these contrasts to MAST [22].

We supply three different kinds of contrasts that are useful for finding biomarkers for clusters:

1. all pair-wise comparisons;
2. each cluster versus all remaining clusters comparisons;
3. hierarchical comparisons, where, for each node in the cluster dendrogram from makeDendrogram, a comparison is performed between the two children nodes.

The last type of contrasts—hierarchical—is our novel and preferred approach for specifying comparisons based on the hierarchical relationships between the clusters, as described in the makeDendrogram step above. Unlike the other comparisons that clusterExperiment implements, the hierarchical comparisons allow a multi-resolution approach, identifying both biomarkers that have widespread differences between large groups of samples, and biomarkers that separate small groups of samples (i.e. individual clusters).

### Visualization

The clusterExperiment package provides many visualization tools in addition to the above algorithmic functions, often making use of existing R and Bioconductor functionality. clusterExperiment brings them together in one package and, most importantly, makes it easy to integrate the clustering results with the visualization of the gene expression data.

For example, one of the most frequently used visualization methods in gene expression studies is the heatmap or pseudo-color image representation of the genes-by-samples expression matrix. clusterExperiment provides a function plotHeatmap, that relies on the aheatmap function in the package NMF [32] and adapts it to be specific to the results of clusterExperiment by optionally plotting the clustering results alongside the samples. Furthermore, a common problem in standard heatmaps is that the hierarchical clustering of the samples with a basic hierarchical clustering algorithm does not exactly correspond to the clustering found by other methods. clusterExperiment uses instead a hierarchical clustering of the *clusters* (described in the section “Merging clusters based on a cluster hierarchy (mergeClusters)”) that keeps the clusters together and, importantly, does so by also placing the most similar clusters close to each other (see Fig 2).

Another example is the function plotClusters for visually comparing large numbers of clusterings, following the work of [33]. This function calculates an alignment of the cluster assignments across samples, along with an ordering of samples, so that the user can visualize the similarity in cluster assignments (see Fig 1b).

Other visualization tools include plotting of the hierarchy of the clusterings (plotDendrogram), plotting the concordance of two clusterings via a barplot (plotBarplot) or heatmap of their contigency table (plotClustersTable), plotting a two-dimensional representation of the data color-coded by cluster (plotReducedDims), and plotting boxplots of the expression levels of an individual gene per cluster (plotFeatureBoxplot). These are all common visualization tools for which the clusterExperiment package implementation makes it simple to integrate the clustering information, and they are all demonstrated in the vignette that accompanies the package.

## Results

### Olfactory epithelium data (Fluidigm C1)

We demonstrate the usage of the clusterExperiment package with a single-cell RNA-Seq dataset of 747 cells run on a Fluidigm C1 machine from neuronal stem cell differentiation in the mouse olfactory epithelium (OE) [34]. The OE is made of four major mature cell types and two progenitor cell populations. Fletcher et al. [34] used RSEC to find 13 experimentally validated clusters that clearly correspond to the known mature and progenitor cell types. The RSEC clusters were used as a starting point to identify the lineage trajectories that produce the major cell types in the OE.

Here, we independently run the entire RSEC workflow on the OE dataset with the function RSEC, which implements our preferred pipeline of subsampling and sequential detection of clusters across many parameter choices. We follow the original paper in preprocessing the data, including filtering poor quality cells and lowly-expressed genes (see section Data used in the Manuscript in S1 Text). However, note that some of the options of RSEC differ from those used in the original article; in particular, we run far more clusterings on the OE data than a typical use case in order to demonstrate the various parameters that can be varied (Table 1), which resulted in 432 separate clusterings.

**Table 1:** Parameters varied when applying RSEC in the analysis of the olfactory epithelium and hypothamlus datasets. See section clusterMany in S1 Text for a complete list of arguments that can be varied in the RSEC workflow.

Parameter	Olfactory	Hypothalamus	Description
# dimensions	20, 50, 100	50	Number of dimensions retained from PCA
*α*	0.1, 0.2	0.1, 0.3	Required similarity for finding a cluster from the co-occurrence matrix from subsampling
*β*	0.8, 0.9	0.9	Required percentage of overlapping samples as *k* is increased to sequentially find a cluster
*k*_0_	4–15	5, 10, …, 35	The initial *k* for the number of clusters to find in each subsampling routine
minSize	1, 5, 10	5	The minimum size of a cluster required of each of the clusterings of clusterMany

We visualize the clustering results from the clusterMany step on the OE data using the function plotClusters in Fig 1b. We observe that the many different clusterings from the clusterMany step generally create similar clusters for the vast majority of the cells, while there exist fewer cells that are noisily assigned to different clusters as the parameters change in different clusterings. This pattern, typical for single-cell sequencing data when making use of both the subsampling and sequential steps, demonstrates the robustness provided by these approaches.

We can visualize how stable different cells were in their clusterings with a heatmap of the co-occurrence matrix (Fig 1c): each entry of the matrix corresponds to the proportion of clusterings in which a pair of samples were clustered together. We can see strong blocks of cells that are almost always clustered together. We also note that the underlying similarity between the clusterings, despite large changes in tuning parameters, also makes the use of consensus across these clustering a logical choice.

This co-occurrence matrix forms the basis for creating a consensus between the different clusterings using the function makeConsensus, shown in Fig 1b. We can see that not all of the cells are assigned to a cluster by makeConsensus. These are samples that are either not consistently co-clustered with other samples across the different clusterings or are unassigned in a high percentage of the clusterings. The reason why such samples are unassigned may vary depending on the dataset: they could correspond to rare cell types, noisy samples, or *doublets*. Without additional, external information, it is very difficult to distinguish between these and clusterExperiment takes the conservative approach of not using such samples for downstream analyses, such as the marker gene detection carried out by getBestFeatures. In the OE dataset the majority of unassigned samples lies in between clusters, suggesting that they do not constitute distinct rare cell populations (S7 Fig).

We further merge together similar clusters from the consensus clustering using mergeClusters, as described in the section “Merging clusters based on a cluster hierarchy (mergeClusters)”. Specifically, mergeClusters creates a hierarchy between clusters and scores each node based on the percentage of genes showing differential expression between samples in the children nodes. Fig 1d visualizes the hierarchy found by makeConsensus as well as which clusters were merged by mergeClusters. We use a simple measure to estimate the proportion of genes that are differentially expressed between children nodes: the proportion of genes called significant at nominal level 0.05 based on the Benjamini and Hochberg [35] procedure for controlling the false discovery rate (FDR); a comparison of the different implemented methods is shown in S1 Fig.

We would also note that clusterExperiment makes it easy to switch from the merged clusters back to the consensus clusters found by makeConsensus, effectively allowing the user to explore the data at two different levels of resolution.

We finally use the function getBestFeatures to find genes that show strong differences in expression between clusters. For each gene, we compute DE statistics for contrasts based on the hierarchy of the tree, as well as a global *F*-statistic. The heatmaps for the top DE genes resulting from both of these approaches are shown in Fig 2 and illustrate that differences between the clusters are much more striking with the hierarchical contrasts, as compared to a global *F*-statistic. Indeed, of the top genes shown in the figures, only 18 genes overlap between the hierarchical and *F* statistics (of 256 genes in each).

Fig 2 also demonstrates clusterExperiment’s heatmap function, plotHeatmap, that seamlessly incorporates the clustering information into the heatmap visualization. The function automatically adds the cluster identifications of individual cells to the heatmap, but also makes use of the hierarchy that is created between the clusters (Fig 1d). In this way, the cluster structure will be respected in the ordering of the samples, unlike a standard hierarchical clustering of the cells, yet ensures that similar clusters will be plotted close to each other rather than in an arbitrary order.

### Hypothalamus data (Drop-Seq)

We further run the RSEC workflow on a set of 14,437 cells sequenced from the hypothalamus of adult mice using Drop-Seq [36] made available in bioconductor format by [37]. For this larger data set, we use a far smaller set of parameters (Table 1) resulting in only 14 clusterings from the clusterMany step. We again follow the preprocessing steps of the original paper, but unlike [36], we cluster all of the cells using RSEC (in [36] the authors clustered only the 3,319 cells with at least 2,000 expressed genes, using the method of [4], and then assigned the remaining cells to those clusters).

Our RSEC workflow generally recapitulates the clusters of [36]. Indeed, only a total of 65 cells differ between RSEC and [36] as to whether they are assigned to neuronal or non-neuronal clusters—the two predominant classes of cells in this system—with neuronal clusters determined based on the expression levels of the cluster of neuronal gene markers *Snap25* and *Syt1* (S2 Fig). Examining gene expression of these neuronal markers shows that the majority of the cells that differ in their neuronal/non-neuronal classification are either correctly identified by RSEC or show markers for both neuronal and non-neuronal cell types (S3 Fig). Similarly, the division of cells into the major subcategories of neuronal and non-neuronal cells of [36], which were decided by [36] based on the expression values of specific marker genes on the set of 3,319 cells, are overwhelmingly conserved between the two approaches (S4 and S5 Figs). One of the larger differences between RSEC and the results of [36] is that RSEC provides classifications for a larger number of cells than [36] (2,348 cells are missing a classification by [36] versus 1,151 for RSEC, with 484 of these not assigned by either). 82% of the cells additionally classified by RSEC are in neuronal clusters.

RSEC also largely preservers the more fine-grained clusters of [36]. For example, the authors of [36] further classify their neuronal clusters into inhibitory and excitatory neurons, (“Glu” and “GABA” in [36]) based on expression of marker genes *Slc17a6* and *Slc32a1*, respectively. We again see that RSEC conserves this split (S4(c) Fig), except for a slight mixing in one of the clusters found by RSEC. Similarly, RSEC creates clusters that separate out both the four large classes of different types of non-neuronal cells (S4(b) Fig), as well as the 11 more fine-grained divisions of these four classes created by [36] (S4(a) Fig) with the exception of the subdivisions of the endothelial cells, which RSEC clusters differently. Furthermore, in the differences we mentioned above—the subdivision of neuronal cells and of endothelial cells—upon examination of the gene markers originally used by [36] to characterize the biological function of their clusters, we find that the differences in our clustering as compared to [36] represent good separation of these markers, and at times better separates the cells based on these biological differences (see Section Comparison of RSEC to clusters of [18] in S1 Text for details).

### Other comparisons via clusterExperiment

**Fig 3:**
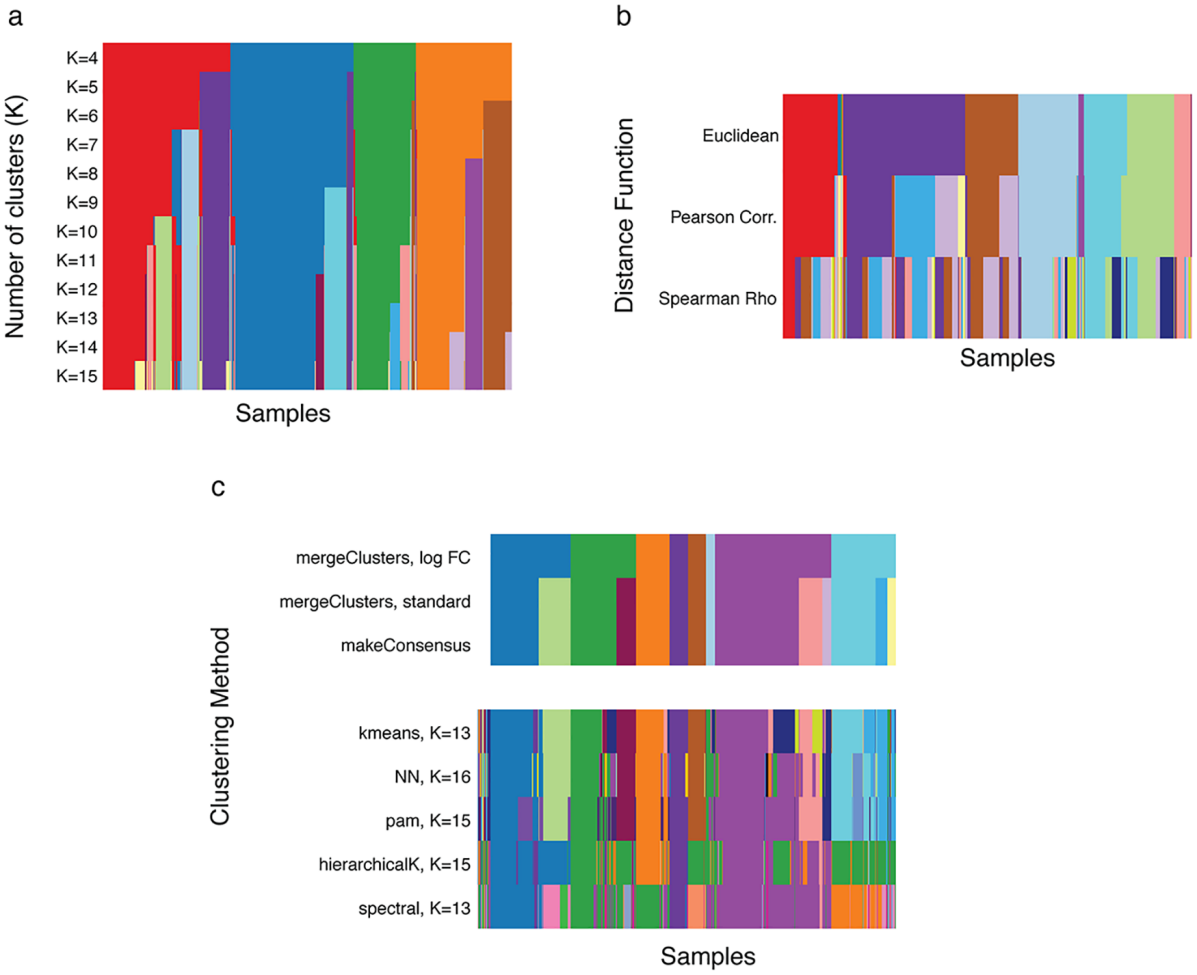
Comparison of methods and tuning parameter choices using clusterMany and plotClusters, demonstrated on the olfactory epithelium dataset. The figure provides examples of using clusterExperiment to compare clustering methods and tuning parameter choices via the function clusterMany to implement the clustering procedures and the function plotClusters to visualize results. (a) shows the clustering results after running PAM with different choices of *K*, the number of clusters. (b) shows the clustering results for different between-sample distance measures. ‘Euclidean’ refers to the standard Euclidean distance; ‘Pearson Corr.’ and ‘Spearman’s Rho’ to a correlation-based distance, *d*(*i*, *j*) = 1/2(1 − *ρ*(*i*, *j*)), where *ρ*(*i*, *j*) is either the standard Pearson correlation coefficient or the robust Spearman rank correlation coefficient between samples *i* and *j*, respectively. (c) shows the clustering results for different choices of clustering algorithms. Each method is shown with the “best” choice of *K*, as determined by the maximum average silhouette width; “NN” refers to a user-defined, nearest-neighbor clustering (see Section Data used in the Manuscript in S1 Text). Also shown is the result of applying the consensus and merging steps of the RSEC workflow to this set of clusterings. The clusterings in (a) and (c) were run with the top 50 PCA dimensions as input. The clusterings in (b) involve comparing different between-gene distance measures and therefore were run directly on the gene expression measures after filtering to the top 1,000 most variable genes, as determined by the median absolute deviation (MAD), a robust version of variance.

We can also use the clusterExperiment package for straightforward implementation and comparison of specific clustering algorithms and parameters, and the clusterExperiment framework makes it easy to store and compare the results. Fig 3 shows several such examples of comparisons available in clusterExperiment: varying the choice of *K* for partitions around medoids (PAM), comparing choices of how to calculate distances between genes, and comparing different clustering algorithms. All of these different choices can be made by a choice of arguments to clusterMany. In comparing the clustering algorithms, we further demonstrate the ability to use a user-defined clustering algorithm based on nearest-neighbor clustering, similar to the package Seurat [38] (see Section Data used in the Manuscript in S1 Text for details of implementation), and include it in the comparison to four algorithms provided by clusterExperiment (for a list of all clustering algorithms currently provided by the packages see Section clusterMany in S1 Text). For this comparison of different algorithms, we also demonstrate how the relevant RSEC workflow steps can be used on these different clustering algorithms to find a consensus clustering, make a hierarchy of clusters, and merge clusters. We also demonstrate in this comparison the ability to require a necessary log-fold change cutoff to be considered differentially expressed for the purposes of merging, see Section mergeClusters in S1 Text.

#### Computational cost

The RSEC analysis on the olfactory data performed 432 clusterings on 747 cells, where each clustering consisted of an iterative sequential analysis, each iteration of the sequential clustering involved 100 clusterings of subsamples of the data, and each clustering used in the subsampling used 10 random restarts of the *k*-means algorithm. In that analysis we demonstrated the possible range of parameters that could be compared with the package, and thus this is a much larger number of clusterings than would often be performed in practice. Particularly, for very large single-cell sequencing studies, fewer than 50 clusterings would normally be sufficient given the computational costs. In S6 Fig, we show the clusterings of the olfactory data from the makeConsensus step applied to different subsets of parameters from the clusterMany step, and the results are quite similar. Similarly, for the analysis of the hypothalamus dataset with 14,437 cells, we used only 14 different parameter combinations (i.e. clusterings) in the clusterMany step; each of those clusterings were the same procedure as the olfactory data—iterative sequential clustering, with each iteration using 100 clusterings of subsamples of the data (though we did not use random restarts of the *k*-means algorithm).

In Table 2 we give the computational time and memory usage of the clustering of the olfactory and hypothalamus datasets, as well as results for running the identical analysis on the hypothalamus data, but for smaller, random subsets of the data.

**Table 2:** Computational costs: For each of the above runs, we give the total number of hours, total CPU time, and maximum memory usage to run the RSEC workflow when parallelized across 15 cores on an AMD Opteron(TM) Processor 6272 node with 270GB of RAM. The olfactory analysis consisted of 432 clusterings, while that of the hypothalamus of only 14.

Dataset	# Cells	Time (hours)	CPU time (hours)	Max RAM (Gb)
Olfactory	747	4.0	50.5	35
Hypothalamus	14,437	30.7	250	151
	10,000	17.0	115.3	95
	5,000	7.2	35.4	63
	1,000	0.70	4.2	54

## Availability and future directions

We have demonstrated the use of a new software framework for clustering and visualizing large gene expression datasets, in particular, single-cell RNA-Seq datasets. The clusterExperiment package implements a wide range of clustering routines appropriate for this type of data, as well as encoding a novel workflow strategy that emphasizes robust clustering. The entire workflow is flexible and extendable by the user to other clustering routines. Furthermore, users can create clusterings externally from the package functions and upload the clustering results to a ClusterExperiment object to make use of the visualization and comparisons capabilities of the package.

clusterExperiment is specifically designed for sample clustering, which is often the primary goal of single-cell RNA-seq analyses. Although the package can be used to cluster genes, some of its functions have to be used with care, as there are different statistical considerations to make when clustering genes rather than samples. Furthermore, the simultaneous clustering of both samples and genes in the same object (e.g., *biclustering*) is not covered by this package.

Similarly, clusterExperiment’s goal is to identify discrete clusters corresponding to cell types, and thus it is not designed for the identification of continuous developmental trajectories. However, several method for the inference of cell trajectories from single-cell RNA-Seq use an initial clustering of cells as a first step in estimating these underlying gradients [39–41]. Our published workflow [42] shows how RSEC can be used in combination with our method Slingshot [39] in an analysis of developmental data, which was the strategy used in [34] for the olefactory data.

The package clusterExperiment is publicly available through the Bioconductor Project, with a detailed manual (vignette) as well as well-documented help pages for each function. The code for implementing all of the analyses shown here is available on the GitHub repository: www.github.com/epurdom/RSECPaper. The analysis in this paper was run using the clusterExperiment package version 2.1.5, R version 3.5.0, and Bioconductor 3.7.

## Supporting information

S1 Text Supplementary text. Supplemental text giving a more detailed description of the RSEC framework and its implementation, as well as information about the analysis of the datasets.
(AUX)

S1 Fig Proportions found null for per node of cluster hierarchy, OE data. (a) Dendrogram of the hierarchical relationship between clusters used for mergeClusters step as well as in finding best features for each cluster. (b) shows for each node in the dendrogram the proportion of genes found differentially expressed between its children’s nodes, for each method implemented in mergeClusters.
(TIF)

S2 Fig Boxplots of the log gene expression values for neuronal markers. Log gene expression of Snap25 or Syt1 for each of the clusters of RSEC using plotFeatureBoxplot. Expression of either Snap25 or Syt1 are used to identify neuronal clusters by the authors of [36].
(TIF)

S3 Fig Expression of main markers of cells differing in neuronal/non-neuronal classification. We isolate the 65 cells that were assigned to neuronal or non-neuronal clusters differently between RSEC and [36] and plot their log expression levels for the markers in [36] using the plotFeatureScatter function: Snap25 (neuronal), Syt1 (neuronal), Olig1 (oligodendrocyte), Cldn5 (endothelial), C1qa (microglia/macrophages), Sox9 (astrocytes, ependymocyte, and tanycytes). Cells in (a) are assigned to a RSEC neuronal cluster, and a non-neuronal cluster in [36] and in (b) assigned to a RSEC non-neuronal cluster, and a neuronal cluster in [36]. The data has been “jittered” so as to be able to see points with the same values. Note the axes for different genes can be on widely different scales.
(TIF)

S4 Fig Comparison of clusters of RSEC and [36], hypothalamus data. We show the results of clustering using the RSEC workflow on the hypothalamus data using plotClusters. (a) shows the clustering results of RSEC on all cells, compared to the clusters of [36]; (b) shows the clustering results of RSEC all cells, grouped by the major subtypes identified by marker genes by [36]; (c) is restricted to those cells identified as neuronal either by RSEC or by [36]. We include below the results of RSEC a color indication as to whether the cells were identified as GABA or Glu by [36] (with white indicating that [36] did not assign those cells to a cluster).
(TIF)

S5 Fig Percentage overlap of RSEC with clusters of [36], hypothalamus data. We plot the percentage of overlap of each RSEC clusters with the classifications of [36] using the plotClustersTable function of clusterExperiment. (a) shows the overlap of RSEC with the major subtype classifications of [36], based on collapsing their clusters via shared marker gene status. (b) shows the overlap of RSEC with the full set of clusters of [36]. Each column corresponds to a cluster from the final mergeClusters step of RSEC. The gray scale shows the distribution of each RSEC cluster across the classifications of [36] on the rows, so that the sum of the percentages of each column equals 1. We calculate the percentages based only on those cells classified by both methods.
(TIF)

S6 Fig Smaller numbers of parameters on OE data. We show the clustering results on the olfactory data, when running makeConsensus on increasingly small choices of parameters in the clusterMany step. Note that this does not require rerunning the (intensive) clusterMany step, but just a selection of clusterings already calculated in the input into the makeConsensus step.
(TIF)

S7 Fig Plotting top two PCA dimensions, OE data. We demonstrate the use of plotReducedDims to show the clustering results of the makeConsensus step on the first two PCA dimensions, with the unassigned samples colored in grey.
(TIF)

S1 Code Source code for version 2.1.5. We provide the source code for the clusterExperiment package, version 2.1.5, used to do the analyses provided in the paper for reproducibility. However, users should not use this source code, but rather follow the Bioconductor installation instructions at https://www.bioconductor.org/install/ for installation of the package.
(GZ) Click here for additional data file.

S1 Vignette Vignette/Manual. We provide the vignette that accompanies the clusterExperiment package, version 2.1.5 used in this paper. The most up-to-date manual can be found at https://bioconductor.org/packages/release/bioc/vignettes/clusterExperiment/inst/doc/clusterExperimentTutorial.html.
(HTML)

## References

[1] Kiselev VY, Kirschner K, Schaub MT, Andrews T, Yiu A, Chandra T, et al SC3: consensus clustering of single-cell RNA-seq data. Nature Methods. 2017;14(5):483–486. 10.1038/nmeth.4236 28346451 PMC5410170

[2] Žurauskienė J, Yau C. pcaReduce: hierarchical clustering of single cell transcriptional profiles. BMC Bioinformatics. 2016;17(1):140 10.1186/s12859-016-0984-y 27005807 PMC4802652

[3] Xu C, Su Z. Identification of cell types from single-cell transcriptomes using a novel clustering method. Bioinformatics (Oxford, England). 2015;31(12):1974–1980. 10.1093/bioinformatics/btv088 PMC6280782 25805722

[4] Macosko EZ, Basu A, Satija R, Nemesh J, Shekhar K, Goldman M, et al Highly Parallel Genome-wide Expression Profiling of Individual Cells Using Nanoliter Droplets. Cell. 2015;161(5):1202–1214. 10.1016/j.cell.2015.05.002 26000488 PMC4481139

[5] Grün D, Lyubimova A, Kester L, Wiebrands K, Basak O, Sasaki N, et al Single-cell messenger RNA sequencing reveals rare intestinal cell types. Nature. 2015;525(7568):251–255. 10.1038/nature14966 26287467

[6] Ntranos V, Kamath GM, Zhang JM, Pachter L, Tse DN. Fast and accurate single-cell RNA-seq analysis by clustering of transcript-compatibility counts. Genome biology. 2016;17(1):1396 10.1186/s13059-016-0970-8 PMC4881296 27230763

[7] Guo M, Wang H, Potter SS, Whitsett JA, Xu Y. SINCERA: A Pipeline for Single-Cell RNA-Seq Profiling Analysis. PLoS computational biology. 2015;11(11):e1004575 10.1371/journal.pcbi.1004575 26600239 PMC4658017

[8] Tseng GC, Wong WH. Tight clustering: a resampling-based approach for identifying stable and tight patterns in data. Biometrics. 2005;61(1):10–16. 10.1111/j.0006-341X.2005.031032.x 15737073

[9] Minaei-bidgoli B, Topchy A, Punch WF. A Comparison of Resampling Methods for Clustering Ensembles. In: In IC-AI; 2004. p. 939–945.

[10] Abul O, Lo A, Alhajj R, Systems FP, and M, 2003. Cluster validity analysis using subsampling. In: 44th Hawaii International Conference on System Sciences; 2003.

[11] Li HG, Wu GQ, Hu XG, Zhang J, Li L, Wu X. K-means clustering with bagging and mapreduce. In: 44th Hawaii International Conference on System Sciences; 2011.

[12] Jia J, Xiao X, Liu B, Jiao L. Bagging-based spectral clustering ensemble selection. Pattern Recognition Letters. 2011;32(10):1456–1467. 10.1016/j.patrec.2011.04.008

[13] Leisch F. Bagged clustering. In: Working Papers SFB Adaptive Information Systems and Modelling in Economics and Management Science. SFB Adaptive Information Systems and Modelling in Economics and Management Science, WU Vienna University of Economics and Business; 1999.

[14] Minaei-Bidgoli B, Parvin H, Alinejad-Rokny H, Alizadeh H, Punch WF. Effects of resampling method and adaptation on clustering ensemble efficacy. Artificial Intelligence Review. 2011;41(1):27–48. 10.1007/s10462-011-9295-x

[15] Monti S, Tamayo P, Mesirov J, Golub T. Consensus clustering: a resampling-based method for class discovery and visualization of gene expression microarray data. Machine learning. 2003;52(1):91–118. 10.1023/A:1023949509487

[16] Ben-Hur A, Guyon I. Detecting Stable Clusters Using Principal Component Analysis In: Functional Genomics. New Jersey: Humana Press; 2003 p. 159–182. 10.1385/1-59259-364-X:159 12710673

[17] Smolkin M, Ghosh D. Cluster stability scores for microarray data in cancer studies. BMC Bioinformatics. 2003;4:36 10.1186/1471-2105-4-36 12959646 PMC200969

[18] Dudoit S, Fridlyand J. Bagging to improve the accuracy of a clustering procedure. Bioinformatics. 2003;19(9):1090–9. 10.1093/bioinformatics/btg038 12801869

[19] Tibshirani R, Walther G. Cluster Validation by Prediction Strength. Journal of Computational and Graphical Statistics. 2012;14(3):511–528. 10.1198/106186005X59243

[20] Lun A, Risso D. SingleCellExperiment: S4 Classes for Single Cell Data; 2017.

[21] Smyth GK. Limma: linear models for microarray data In: Gentleman R, Carey V, Dudoit S, Irizarry R, H W, editors. Bioinformatics and Computational Biology Solutions using R and Bioconductor. New York: Springer; 2005 p. 397–420.

[22] Finak G, McDavid A, Yajima M, Deng J, Gersuk V, Shalek AK, et al MAST: a flexible statistical framework for assessing transcriptional changes and characterizing heterogeneity in single-cell RNA sequencing data. Genome biology. 2015;16(1):1–13. 10.1186/s13059-015-0844-5 26653891 PMC4676162

[23] Robinson MD, Mccarthy DJ, Smyth GK. edgeR: a Bioconductor package for differential expression analysis of digital gene expression data. Bioinformatics (Oxford, England). 2010;26(1):139–140. 10.1093/bioinformatics/btp616 PMC2796818 19910308

[24] Storey J. A Direct Approach to False Discovery Rates. Journal of the Royal Statistical Society Series B (Statistical Methodology). 2002;64(3):479–498. 10.1111/1467-9868.00346

[25] Efron B. Large-Scale Simultaneous Hypothesis Testing: The Choice of a Null Hypothesis. Journal of the American Statistical Association. 2004;99(465):96–104. 10.1198/016214504000000089

[26] Pounds S, Cheng C. Improving false discovery rate estimation. Bioinformatics (Oxford, England). 2004;20(11):1737–1745. 10.1093/bioinformatics/bth160 14988112

[27] Meinshausen N, Buhlmann P. Lower bounds for the number of false null hypotheses for multiple testing of associations under general dependence structures. Biometrika. 2005;92(4):893–907. 10.1093/biomet/92.4.893

[28] Jin Jiashun, Cai T Tony. Estimating the Null and the Proportion of Nonnull Effects in Large-Scale Multiple Comparisons. Journal of the American Statistical Association. 2007;102(478):495–506. 10.1198/016214507000000167

[29] Love MI, Huber W, Anders S. Moderated estimation of fold change and dispersion for RNA-Seq data with DESeq2. bioRxiv. 2014;. 10.1186/s13059-014-0550-8 PMC4302049 25516281

[30] Law CW, Chen Y, Shi W, Smyth GK. voom: Precision weights unlock linear model analysis tools for RNA-seq read counts. Genome Biology. 2014;15(2):R29 10.1186/gb-2014-15-2-r29 24485249 PMC4053721

[31] Van den Berge K, Perraudeau F, Soneson C, Love MI, Risso D, Vert JP, et al Observation Weights to Unlock Bulk Rna-Seq Tools for Zero Inflation and Single-Cell Applications. Genome Biology. 2018;19(24). 10.1186/s13059-018-1406-4 29478411 PMC6251479

[32] Gaujoux R, Seoighe C. A flexible R package for nonnegative matrix factorization. BMC Bioinformatics. 2010;11:367 10.1186/1471-2105-11-367 20598126 PMC2912887

[33] Wilkerson MD, Hayes DN. ConsensusClusterPlus: a class discovery tool with confidence assessments and item tracking. Bioinformatics (Oxford, England). 2010;26(12):1572–1573. 10.1093/bioinformatics/btq170 PMC2881355 20427518

[34] Fletcher RB, Das D, Gadye L, Street KN, Baudhuin A, Wagner A, et al Deconstructing Olfactory Stem Cell Trajectories at Single-Cell Resolution. Cell stem cell. 2017;20(6):817–830.e8. 10.1016/j.stem.2017.04.003 28506465 PMC5484588

[35] Benjamini Y, Hochberg Y. Controlling the False Discovery Rate: A Practical and Powerful Approach to Multiple Testing. Journal of the Royal Statistical Society Series B (Methodological). 1995;57(1):289–300.

[36] Chen R, Wu X, Jiang L, Zhang Y. Single-Cell RNA-Seq Reveals Hypothalamic Cell Diversity. Cell Reports. 2017;18(13):3227–3241. 10.1016/j.celrep.2017.03.004. 28355573 PMC5782816

[37] at the Sanger Institute HG. scRNA-Seq Datasets; 2018. Available from: https://hemberg-lab.github.io/scRNA.seq.datasets/.

[38] Butler A, Hoffman P, Smibert P, Papalexi E, Satija R. Integrating single-cell transcriptomic data across different conditions, technologies, and species. Nature Biotechnology. 2018;. 10.1038/nbt.4096 29608179 PMC6700744

[39] Street K, Risso D, Fletcher RB, Das D, Ngai J, Yosef N, et al Slingshot: Cell lineage and pseudotime inference for single-cell transcriptomics. BMC Genomics. 2018;19:477 10.1186/s12864-018-4772-0 29914354 PMC6007078

[40] Ji Z, Ji H. TSCAN: Pseudo-time reconstruction and evaluation in single-cell RNA-seq analysis. Nucleic acids research. 2016;44(13):e117–e117. 10.1093/nar/gkw430 27179027 PMC4994863

[41] Shin J, Berg DA, Zhu Y, Shin JY, Song J, Bonaguidi MA, et al Single-cell RNA-seq with waterfall reveals molecular cascades underlying adult neurogenesis. Cell stem cell. 2015;17(3):360–372. 10.1016/j.stem.2015.07.013 26299571 PMC8638014

[42] Perraudeau F, Risso D, Street K, Purdom E, Dudoit S. Bioconductor workflow for single-cell RNA sequencing: Normalization, dimensionality reduction, clustering, and lineage inference. F1000Research. 2017;6 doi: 10.12688/f1000research.12122.1 28868140 10.12688/f1000research.12122.1 PMC5558107

