# Correction: clusterExperiment and RSEC: A Bioconductor package and framework for clustering of single-cell and other large gene expression datasets

**DOI:** 10.1371/journal.pcbi.1006727

**Published:** 2019-01-11

**Authors:** The PLOS Computational Biology Staff

The wrong file was uploaded for the Supporting Information Supplementary text. Please view the correct supplementary text file below. The publisher apologizes for the error.

## Supporting information

S1 Text Supplementary text. Supplemental text giving a more detailed description of the RSEC framework and its implementation, as well as information about the analysis of the datasets.
(PDF)
