# Supplementary figures and images for: clusterExperiment and RSEC: A Bioconductor package and framework for clustering of single-cell and other large gene expression datasets
Source: PLoS Comput Biol. 2018 Sep 4;14(9):e1006378. doi: 10.1371/journal.pcbi.1006378 (PMC6138422; doi:10.1371/journal.pcbi.1006378)

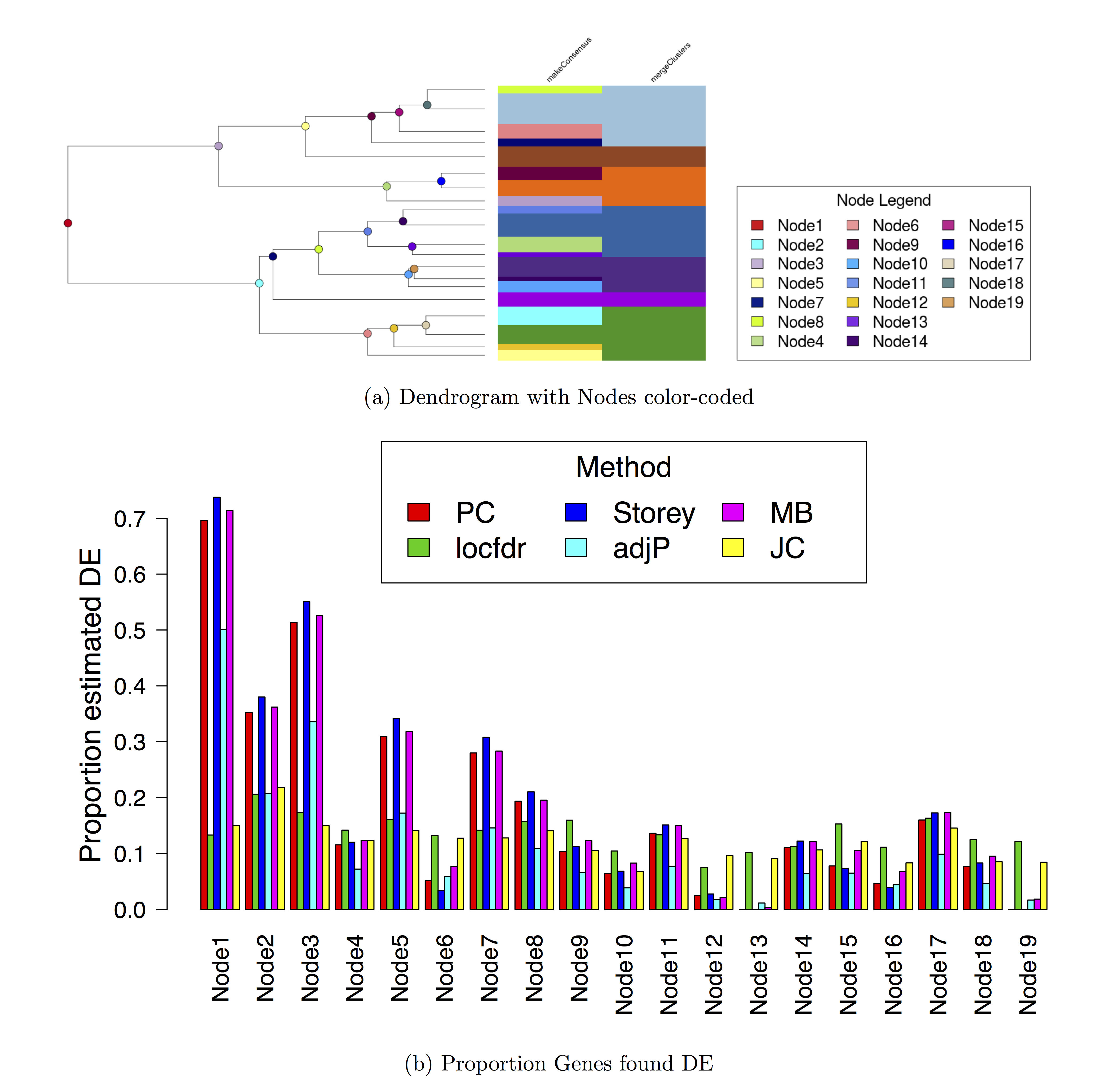

Supplement: S1 Fig — (a) Dendrogram of the hierarchical relationship between clusters used for mergeClusters step as well as in finding best features for each cluster. (b) shows for each node in the dendrogram the proportion of genes found differentially expressed between its children’s nodes, for each method implemented in mergeClusters. (TIF) [file pcbi.1006378.s002.tif]

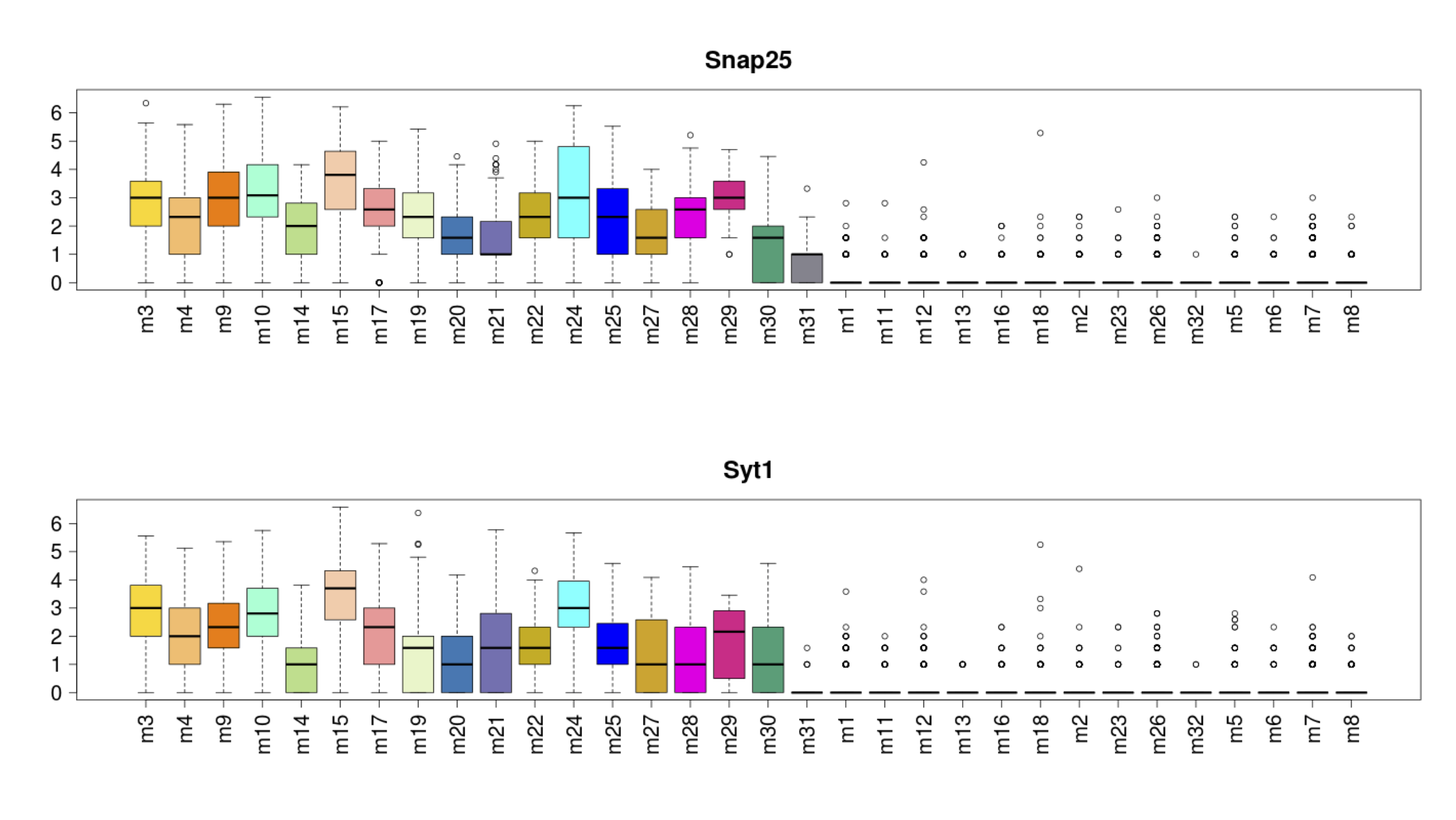

Supplement: S2 Fig — Log gene expression of Snap25 or Syt1 for each of the clusters of RSEC using plotFeatureBoxplot. Expression of either Snap25 or Syt1 are used to identify neuronal clusters by the authors of [36]. (TIF) [file pcbi.1006378.s003.tif]

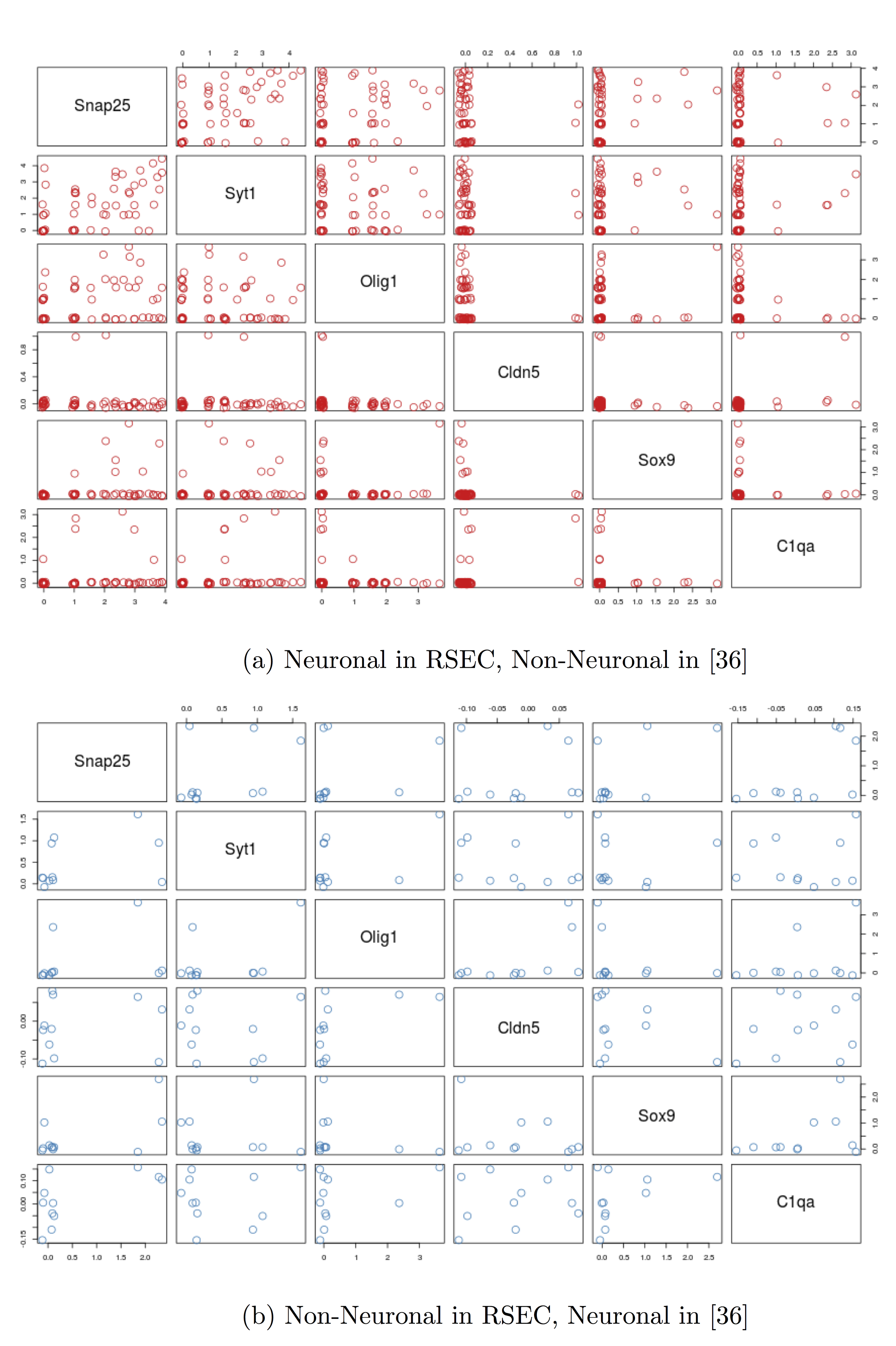

Supplement: S3 Fig — We isolate the 65 cells that were assigned to neuronal or non-neuronal clusters differently between RSEC and [36] and plot their log expression levels for the markers in [36] using the plotFeatureScatter function: Snap25 (neuronal), Syt1 (neuronal), Olig1 (oligodendrocyte), Cldn5 (endothelial), C1qa (microglia/macrophages), Sox9 (astrocytes, ependymocyte, and tanycytes). Cells in (a) are assigned to a RSEC neuronal cluster, and a non-neuronal cluster in [36] and in (b) assigned to a RSEC non-neuronal cluster, and a neuronal cluster in [36]. The data has been “jittered” so as to be able to see points with the same values. Note the axes for different genes can be on widely different scales. (TIF) [file pcbi.1006378.s004.tif]

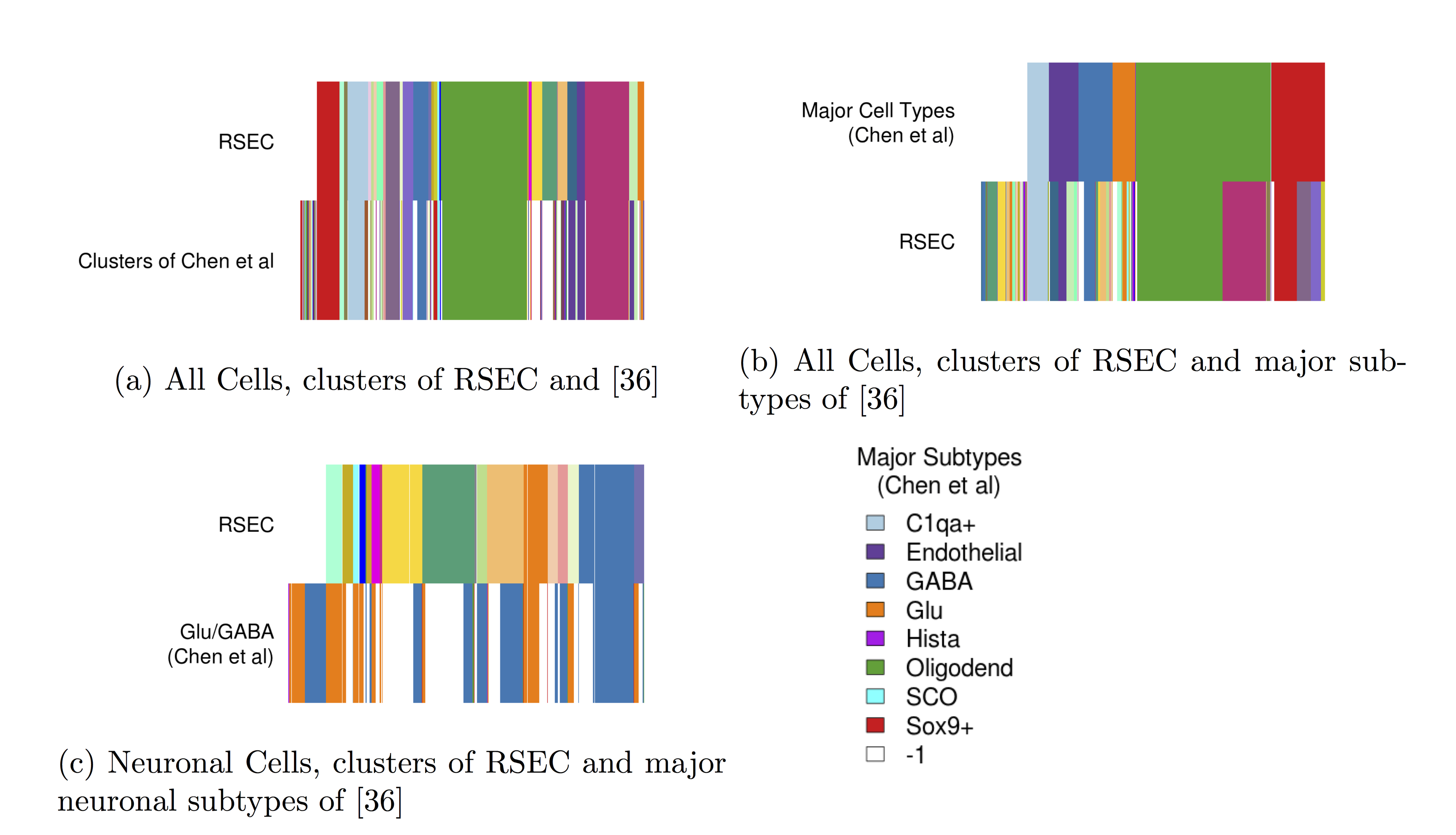

Supplement: S4 Fig — We show the results of clustering using the RSEC workflow on the hypothalamus data using plotClusters. (a) shows the clustering results of RSEC on all cells, compared to the clusters of [36]; (b) shows the clustering results of RSEC all cells, grouped by the major subtypes identified by marker genes by [36]; (c) is restricted to those cells identified as neuronal either by RSEC or by [36]. We include below the results of RSEC a color indication as to whether the cells were identified as GABA or Glu by [36] (with white indicating that [36] did not assign those cells to a cluster). (TIF) [file pcbi.1006378.s005.tif]

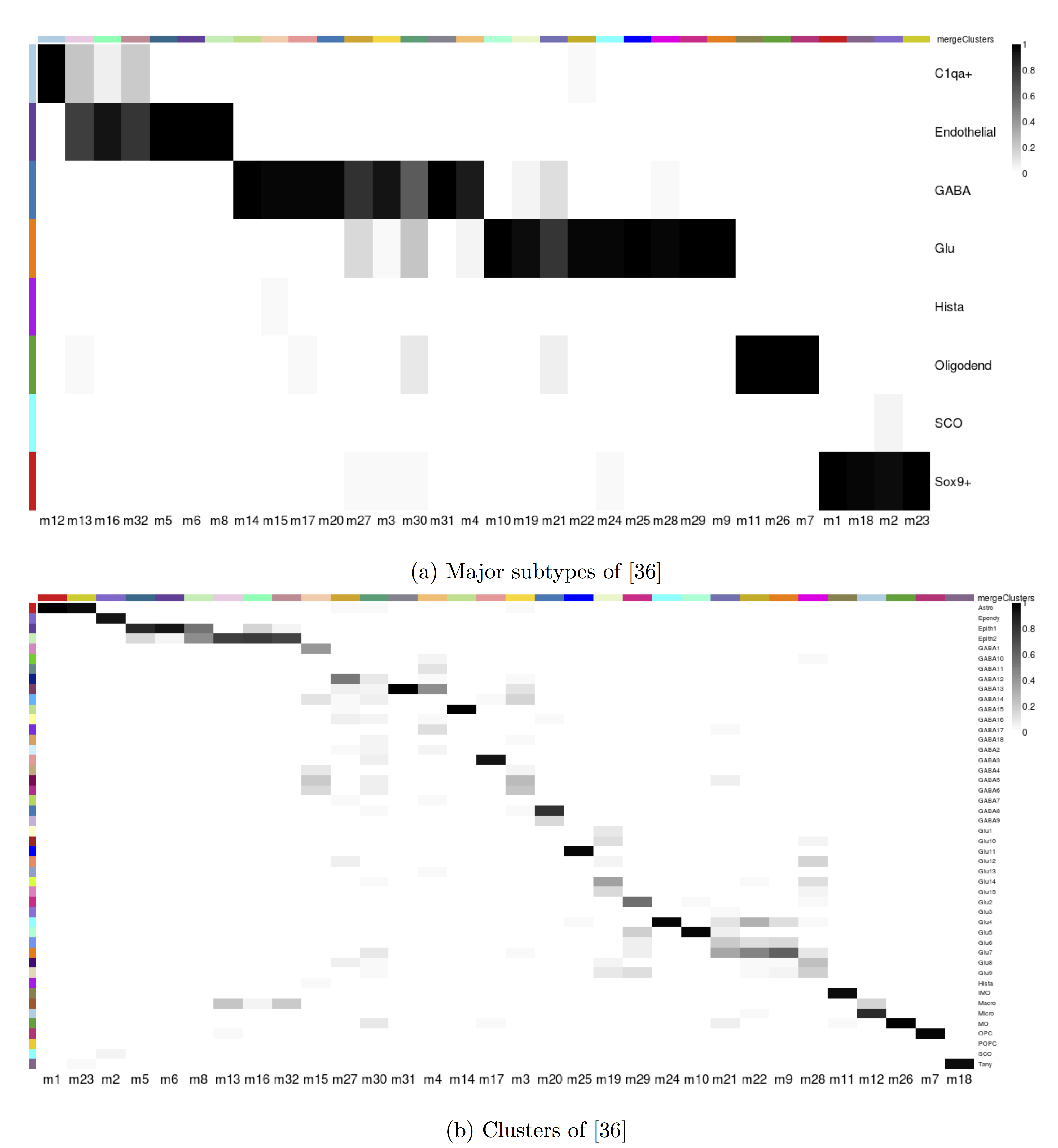

Supplement: S5 Fig — We plot the percentage of overlap of each RSEC clusters with the classifications of [36] using the plotClustersTable function of clusterExperiment. (a) shows the overlap of RSEC with the major subtype classifications of [36], based on collapsing their clusters via shared marker gene status. (b) shows the overlap of RSEC with the full set of clusters of [36]. Each column corresponds to a cluster from the final mergeClusters step of RSEC. The gray scale shows the distribution of each RSEC cluster across the classifications of [36] on the rows, so that the sum of the percentages of each column equals 1. We calculate the percentages based only on those cells classified by both methods. (TIF) [file pcbi.1006378.s006.tif]

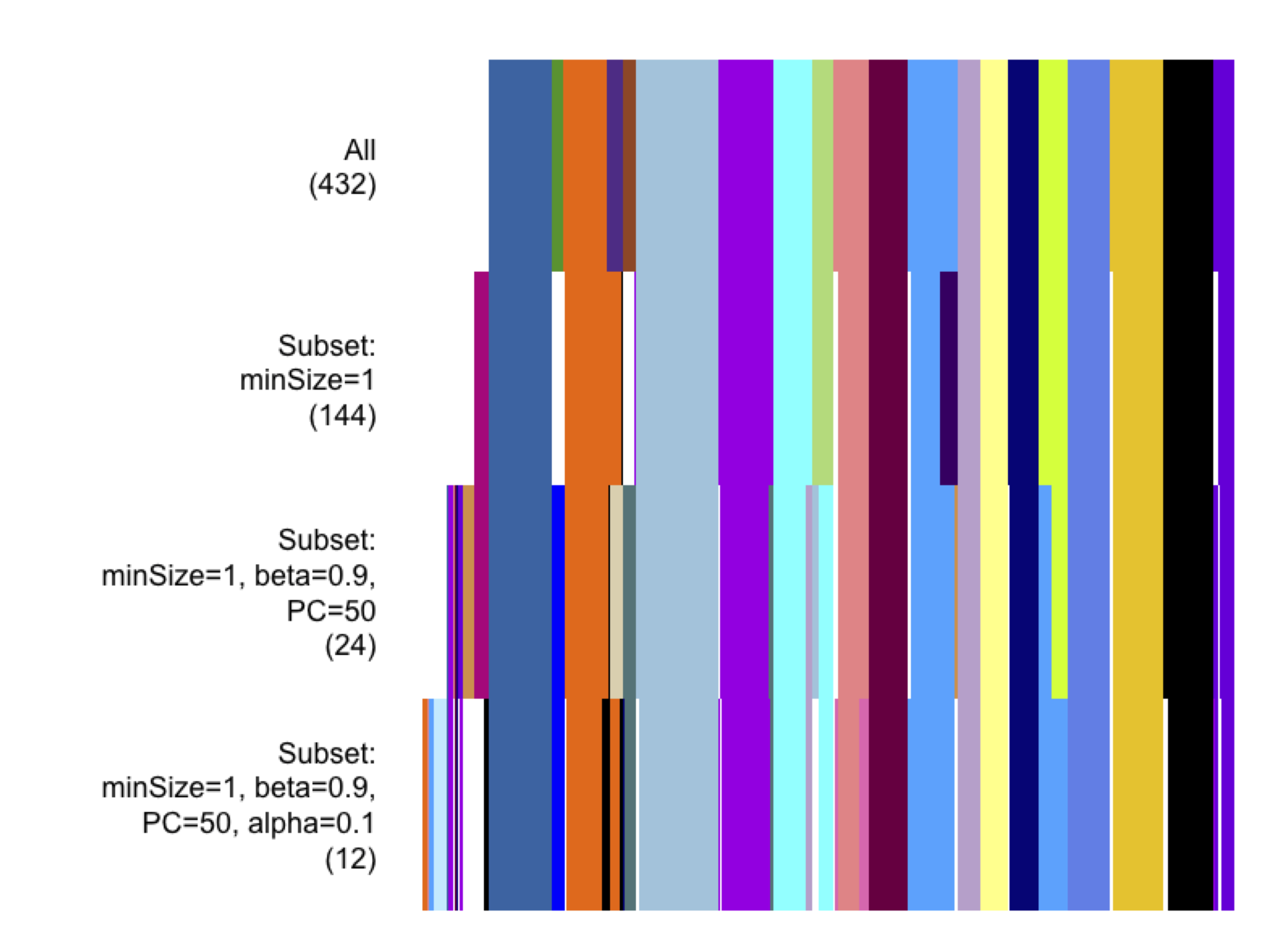

Supplement: S6 Fig — We show the clustering results on the olfactory data, when running makeConsensus on increasingly small choices of parameters in the clusterMany step. Note that this does not require rerunning the (intensive) clusterMany step, but just a selection of clusterings already calculated in the input into the makeConsensus step. (TIF) [file pcbi.1006378.s007.tif]

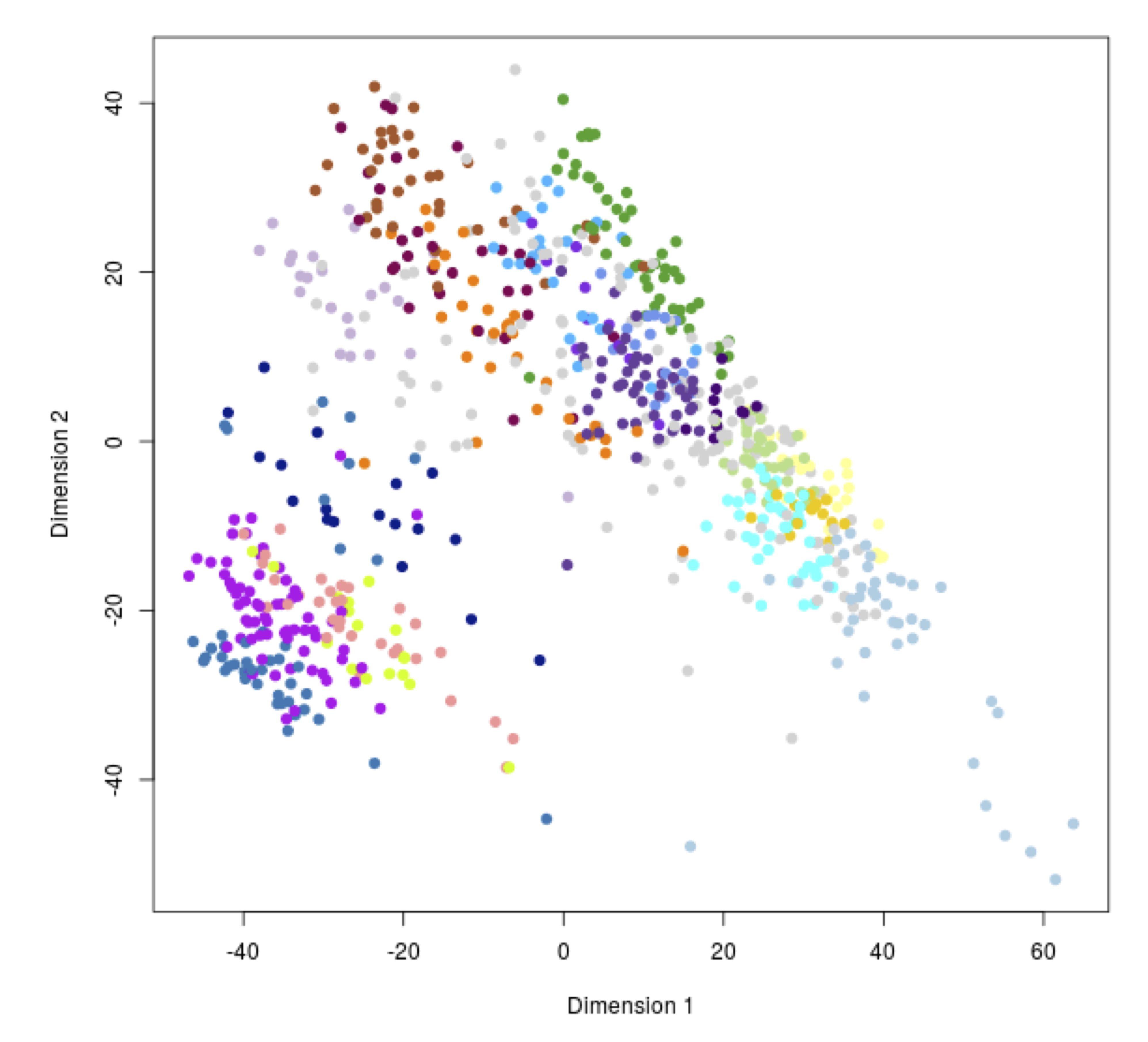

Supplement: S7 Fig — We demonstrate the use of plotReducedDims to show the clustering results of the makeConsensus step on the first two PCA dimensions, with the unassigned samples colored in grey. (TIF) [file pcbi.1006378.s008.tif]
