# Supplementary material for: clusterExperiment and RSEC: A Bioconductor package and framework for clustering of single-cell and other large gene expression datasets
Source: PLoS Comput Biol. 2018 Sep 4;14(9):e1006378. doi: 10.1371/journal.pcbi.1006378 (PMC6138422; doi:10.1371/journal.pcbi.1006378)
Supplement: S1 Code — We provide the source code for the clusterExperiment package, version 2.1.5, used to do the analyses provided in the paper for reproducibility. However, users should not use this source code, but rather follow the Bioconductor installation instructions at https://www.bioconductor.org/install/ for installation of the package. (GZ) [file pcbi.1006378.s009.gz › clusterExperiment/inst/doc/clusterExperimentTutorial.html]

clusterExperiment Vignette


# clusterExperiment Vignette

Elizabeth Purdom and Davide Risso

#### *2018-07-04*

# Contents

- 1 Introduction
  - 1.1 The RSEC clustering workflow
- 2 Quickstart
  - 2.1 The Data
  - 2.2 Filtering and normalization
  - 2.3 Clustering with RSEC
    - 2.3.1 The output
    - 2.3.2 Visualizing the output
  - 2.4 Rerunning RSEC with different parameters
  - 2.5 Finding Features related to the clusters
- 3 Overview of the clustering workflow
  - 3.1 Step 1: Clustering with `clusterMany`
  - 3.2 Step 2: Find a consensus with `makeConsensus`
  - 3.3 Step 3: Merge clusters together with `makeDendrogram` and `mergeClusters`
  - 3.4 RSEC
- 4 ClusterExperiment Objects
  - 4.1 Subsetting ClusterExperiment objects
  - 4.2 Samples not assigned to a cluster (Negative Valued Cluster Assignments)
  - 4.3 Dimensionality reduction and SingleCellExperiment Class
- 5 Visualizing the data
  - 5.1 Cluster Alignment plot with `plotClusters`
    - 5.1.1 Manipulations of colors
    - 5.1.2 Variant version: `plotClustersWorkflow`
  - 5.2 Heatmap including the clusters with `plotHeatmap`
    - 5.2.1 Displaying clustering or sample information
    - 5.2.2 Additional options for clustering/ordering samples
    - 5.2.3 Using separate input data for clustering and for visualization
    - 5.2.4 Setting the breaks
  - 5.3 Dendrogram of clusters with `plotDendrogram`
    - 5.3.1 Changing the leaf and plot type
    - 5.3.2 Changing the information plotted with dendrogram
- 6 The clustering workflow
  - 6.1 clusterMany
    - 6.1.1 Base clustering algorithms and the ClusterFunction class
    - 6.1.2 Internal clustering procedures
    - 6.1.3 Arguments of `clusterMany`
    - 6.1.4 Example changing the distance function
    - 6.1.5 Example using a user-defined clustering algorithm
    - 6.1.6 Dealing with large numbers of clusterings
  - 6.2 Create a unified cluster from many clusters with `makeConsensus`
    - 6.2.1 Consensus from subsets of clusterings
  - 6.3 Creating a Hierarchy of Clusters and Merging clusters
    - 6.3.1 makeDendrogram and plotDendrogram
    - 6.3.2 Merging clusters with little differential expression
  - 6.4 Keeping track of and rerunning elements of the workflow
    - 6.4.1 Designate a Final Clustering
  - 6.5 RSEC
- 7 Finding Features related to a Clustering
  - 7.1 Types of Significance Tests
    - 7.1.1 All Pairwise
    - 7.1.2 One Against All
    - 7.1.3 Dendrogram
  - 7.2 DE Analysis for count and other RNASeq data
  - 7.3 Piping into other DE routines
  - 7.4 Multiple Testing adjustments
- 8 Working with other `assays()`
- 9 Session Information
- References

# 1 Introduction

The goal of this package is to encourage the user to try many different clustering algorithms in one package structure, and we provide strategies for creating a unified clustering from these many clustering resutls. We give tools for running many different clusterings and choices of parameters. We also provide visualization to compare many different clusterings and algorithm tools to find common shared clustering patterns. We implement common post-processing steps unrelated to the specific clustering algorithm (e.g. subsampling the data for stability, finding cluster-specific markers via differential expression, etc).

The other main goal of this package is to implement strategies that we have developed in the RSEC algorithm (Resampling-based Sequential Ensemble Clustering) for finding a single robust clustering based on the many clusterings that the user might create by perturbing various parameters of a clustering algorithm. There are several steps to these strategies that we call our standard clustering workflow. The `RSEC` function is our preferred realization of this workflow that depends on subsampling and other ensemble methods to provide robust clusterings, particularly for single-cell sequencing experiments and other large mRNA-Seq experiments.

We also provide a class `ClusterExperiment` that inherits from `SummarizedExperiment` to store the many clusterings and related information, and a class `ClusterFunction` that encodes a clustering routine so that users can create customized clustering routines in a standardized way that can interact with our clustering workflow algorithms.

All of our methods also have a barebones version that allows input of matrices and greater control. This comes at the expense of the user having to manage and keep track of the clusters, input data, transformation of the data, etc. We do not discuss these barebone versions in this tutorial. Instead, we focus on using the `SummarizedExperiment` object as the input and working with the resulting `ClusterExperiment` object. See the help pages of each method for more on how to allow for matrix input.

Although this package was developed with (single-cell) RNA-seq data in mind, its use is not limited to RNA-seq or even to gene expression data. Any dataset characterized by high dimensionality could benefit from the methods implemented here.

## 1.1 The RSEC clustering workflow

The package encodes many common practices that are shared across clustering algorithms, like subsampling the data, computing silhouette width, sequential clustering procedures, and so forth. It also provides novel strategies that we developed as part of the RSEC algorithm (Resampling-based Sequential Ensemble Clustering) .

As mentioned above, RSEC is a specific algorithm for creating a clustering that follows these basic steps:

- Implement many different clusterings using different choices of parameters using the function `clusterMany`. This results in a large collection of clusterings, where each clustering is based on different parameters.
- Find a unifying clustering across these many clusterings using the `makeConsensus` function.
- Determine whether some clusters should be merged together into larger clusters. This involves two steps:
  - Find a hierarchical clustering of the clusters found by `makeConsensus` using `makeDendrogram`
  - Merge together clusters of this hierarchy based on the percentage of differential expression, using `mergeClusters`.

The basic premise of RSEC is to find small, robust clusters of samples, and then merge them into larger clusters as relevant. We find that many algorithmic methods for choosing the appropriate number of clusters for methods err on the side of too few clusters. However, we find in practice that we tend to prefer to err on finding many clusters and then merging them based on examining the data.

The `RSEC` function is a wrapper around these steps that makes many specific choices in this basic workflow. However, many steps of this workflow are useful for users separately and for this reason, the `clusterExperiment` package generalizes this workflow so that the user can follow this workflow with their own choices. We call this generalization the clustering workflow, as oppose to the specific choices set in `RSEC`.

Users can also run or add their own clusters to this workflow at different stages. Additional functionality for creating a single clustering is also available in the `clusterSingle` function, and the user should see the documentation in the help page of that function.

# 2 Quickstart

In this section we give a quick introduction to the package and the `RSEC` wrapper which creates the clustering. We will also demonstrate how to find features (biomarkers) that go along with the clusters.

## 2.1 The Data

We will make use of a single cell RNA sequencing experiment made available in the `scRNAseq` package.

```
set.seed(14456) ## for reproducibility, just in case
library(scRNAseq)
data("fluidigm")
```

We will use the `fluidigm` dataset (see `help("fluidigm")`). This dataset is stored as a SummarizedExperiment object. We can access the data with `assay` and metadata on the samples with `colData`.

```
assay(fluidigm)[1:5,1:10]
```

```
##          SRR1275356 SRR1274090 SRR1275251 SRR1275287 SRR1275364 SRR1275269
## A1BG              0          0          0          0          0          0
## A1BG-AS1          0          0          0          0          0          0
## A1CF              0          0          0          0          0          0
## A2M               0          0          0         31          0         46
## A2M-AS1           0          0          0          0          0          0
##          SRR1275263 SRR1275242 SRR1275338 SRR1274117
## A1BG              0          0          0          0
## A1BG-AS1          0          0          0          0
## A1CF              0          0          0          0
## A2M               0          0          0         29
## A2M-AS1           0          0          0        133
```

```
colData(fluidigm)[,1:5]
```

```
## DataFrame with 130 rows and 5 columns
##               NREADS  NALIGNED    RALIGN TOTAL_DUP    PRIMER
##            <numeric> <numeric> <numeric> <numeric> <numeric>
## SRR1275356  10554900   7555880   71.5862   58.4931 0.0217638
## SRR1274090    196162    182494   93.0323   14.5122 0.0366826
## SRR1275251   8524470   5858130   68.7213   65.0428 0.0351827
## SRR1275287   7229920   5891540   81.4884   49.7609 0.0208685
## SRR1275364   5403640   4482910   82.9609   66.5788 0.0298284
## ...              ...       ...       ...       ...       ...
## SRR1275259   5949930   4181040   70.2705   52.5975 0.0205253
## SRR1275253  10319900   7458710   72.2747   54.9637 0.0205342
## SRR1275285   5300270   4276650   80.6873   41.6394 0.0227383
## SRR1275366   7701320   6373600     82.76   68.9431 0.0266275
## SRR1275261  13425000   9554960   71.1727   62.0001 0.0200522
```

```
NCOL(fluidigm) #number of samples
```

```
## [1] 130
```

## 2.2 Filtering and normalization

While there are 130 samples, there are only 65 cells, because each cell is sequenced twice at different sequencing depth. We will limit the analysis to the samples corresponding to high sequencing depth.

```
se <- fluidigm[,colData(fluidigm)[,"Coverage_Type"]=="High"]
```

We also filter out lowly expressed genes: we retain only those genes with at least 10 reads in at least 10 cells.

```
wh_zero <- which(rowSums(assay(se))==0)
pass_filter <- apply(assay(se), 1, function(x) length(x[x >= 10]) >= 10)
se <- se[pass_filter,]
dim(se)
```

```
## [1] 7069   65
```

This removed 19186 genes out of 26255. We now have 7069 genes (or features) remaining. Notice that it is important to remove genes with zero counts in all samples (we had 9673 genes which were zero in all samples here). Otherwise, PCA dimensionality reductions and other implementations may have a problem.

Normalization is an important step in any RNA-seq data analysis and many different normalization methods have been proposed in the literature. Comparing normalization methods or finding the best performing normalization in this dataset is outside of the scope of this vignette. Instead, we will use a simple quantile normalization that will at least make our clustering reflect the biology rather than the difference in sequencing depth among the different samples.

```
fq <- round(limma::normalizeQuantiles(assay(se)))
assays(se) <- list(normalized_counts=fq)
```

As one last step, we are going to change the name of the columns “Cluster1” and “Cluster2” that some in the dataset and refer to published clustering results from the paper; we will use the terms “Published1” and “Published2” to better distinguish them in later plots from other clustering we will do.

```
wh<-which(colnames(colData(se)) %in% c("Cluster1","Cluster2"))
colnames(colData(se))[wh]<-c("Published1","Published2")
```

## 2.3 Clustering with RSEC

We will now run `RSEC` to find clusters of the cells using the default settings. We set `isCount=TRUE` to indicate that the data in `se` is count data, so that the log-transform and other count methods should be applied. We also choose the number of cores on which we want to run the operation in parallel via the parameter `ncores`. This is a relatively small number of samples, compared to most single-cell sequencing experiments, so we choose cluster on the top 10 PCAs of the data by setting `reduceMethod="PCA"` and `nReducedDims=10` (the default is 50). Finally, we set the minimum cluster size in our ensemble clustering to be 3 cells (`consensusMinSize=3`). We do this not for biological reasons, but for instructional purposes to allow us to get a larger number of clusters.

Because this procedure is slightly computationally intensive, depending on the user’s machine, we have set this code to not run so that the vignette will compile quickly upon installation. However, it doesn’t take very long (roughly 1-2 minutes) so we recommend users try it themselves. The following code is the example of a common run of RSEC:

```
library(clusterExperiment)
system.time(rsecFluidigm<-RSEC(se, isCount = TRUE, 
    reduceMethod="PCA", nReducedDims=10,
    ncores=1, random.seed=176201))
```

However, to exactly replicate the results here, we will set a large number of parameters to make sure that the vignette is back-compatible in case some defaults change. We will explain these parameters as we go along, but many of these parameters are the default.

```
library(clusterExperiment)
system.time(rsecFluidigm<-RSEC(se, 
  isCount = TRUE, 
    k0s = 4:15, 
  alphas=c(0.1, 0.2, 0.3), 
  betas = 0.9, 
  reduceMethod="PCA",
  nReducedDims=10,
  minSizes=1,
 clusterFunction="hierarchical01",
 consensusMinSize=3,
 consensusProportion=0.7,
 dendroReduce= "mad",
 dendroNDims=1000,
 mergeMethod="adjP",
 mergeCutoff=0.03,
 ncores=1,
 random.seed=176201))
```

Instead we have saved the results from this call as a data object in the package and will use the following code to load it into the vignette:

```
#don't call this routine if you ran the above code.
#it will overwrite the rsecFluidigm you made
library(clusterExperiment)
data("rsecFluidigm")
metadata(rsecFluidigm)$packageVersion
```

```
## [1] '2.1.3.9014'
```

### 2.3.1 The output

We can look at the object that was created.

```
rsecFluidigm
```

```
## class: ClusterExperiment 
## dim: 7069 65 
## reducedDimNames: PCA 
## filterStats: mad_makeConsensus 
## -----------
## Primary cluster type: mergeClusters 
## Primary cluster label: mergeClusters 
## Table of clusters (of primary clustering):
## -1 m1 m2 m3 m4 m5 
## 13 15 27  4  3  3 
## Total number of clusterings: 38 
## Dendrogram run on 'makeConsensus' (cluster index: 2)
## -----------
## Workflow progress:
## clusterMany run? Yes 
## makeConsensus run? Yes 
## makeDendrogram run? Yes 
## mergeClusters run? Yes
```

The print out tells us about the clustering(s) that were created (namely 38 clusterings) and which steps of the workflow have been called (all of them have because we used the wrapper `RSEC` that does the whole thing). Recall from our brief description above that RSEC clusters the data many times using different parameters before finding an consensus clustering. All of these intermediate clusterings are saved. Each of these intermediate clusterings used subsampling of the data and sequential clustering of the data to find the clustering, while the different clusterings represent the different parameters that were adjusted.

We can see that `rsecFluidigm` has a built in (S4) class called a `ClusterExperiment` object. This is a class built for this package and explained in the section on ClusterExperiment Objects. In the object `rsecFluidigm` the clusterings are stored along with corresponding information for each clustering. Furthermore, all of the information in the original `SummarizedExperiment` is retained. The print out also tells us information about the “primaryCluster” of `rsecFluidigm`. Each `ClusterExperiment` object has a “primaryCluster”, which is the default cluster that the many functions will use unless specified by the user. We are told that the “primaryCluster” for `rsecFluidigm` is has the label “mergeClusters” – which is the defaul label given to the last cluster of the `RSEC` function because the last call of the `RSEC` function is to `mergeClusters`.

There are many accessor functions that help you get at the information in a `ClusterExperiment` object and some of the most relevant are described in the section on ClusterExperiment Objects. (`ClusterExperiment` objects are S4 objects, and are not lists).

For right now we will only mention the most basic such function that retrieves the actual cluster assignments. The final clustering created by `RSEC` is saved as the `primary` clustering of the object.

```
head(primaryCluster(rsecFluidigm),20)
```

```
##  [1] -1 -1  2  2 -1 -1 -1  1  1  1  2  2 -1  2  3  5 -1  1  3 -1
```

```
tableClusters(rsecFluidigm)
```

```
## 
## -1 m1 m2 m3 m4 m5 
## 13 15 27  4  3  3
```

The clusters are encoded by consecutive integers. Notice that some of the samples are assigned the value of `-1`. `-1` is the value assigned in this package for samples that are not assigned to any cluster. Why certain samples are not clustered depends on the underlying choices of the clustering routine and we won’t get into here until we learn a bit more about RSEC. Another special value is `-2` discussed in the section on ClusterExperiment objects

This final result of RSEC is the result of running many clusterings and finding the ensembl consensus between them. All of the these intermediate clusterings are saved in `rsecFluidigm` object. They can be accessed by the `clusterMatrix` function, that returns a matrix where the columns are the different clusterings and the rows are samples. We show a subset of this matrix here:

```
head(clusterMatrix(rsecFluidigm)[,1:4])
```

```
##            mergeClusters makeConsensus k0=4,alpha=0.1 k0=5,alpha=0.1
## SRR1275356            -1            -1             -1             -1
## SRR1275251            -1            -1             -1             -1
## SRR1275287             2             5              3              3
## SRR1275364             2             3             -1             -1
## SRR1275269            -1            -1             -1             -1
## SRR1275263            -1            -1             -1             -1
```

The “mergeClusters” clustering is the final clustering from RSEC and matches the primary clustering that we saw above. The “makeConsensus” clustering is the result of the initial ensembl concensus among all of the many clusterings that were run, while “mergeClusters” is the result of merging smaller clusters together that did not show enough signs of differences between clusters. The remaining clusters are the result of changing the parameters, and a couple of such clusterings a shown in the above printout of the cluster matrix.

The column names are the `clusterLabels` for each clustering and can be accessed (and assigned new values!) via the `clusterLabels` function.

```
head(clusterLabels(rsecFluidigm))
```

```
## [1] "mergeClusters"  "makeConsensus"  "k0=4,alpha=0.1" "k0=5,alpha=0.1"
## [5] "k0=6,alpha=0.1" "k0=7,alpha=0.1"
```

We can see the names of more clusterings, and see that the different parameter values tried in each clustering are saved in the names of the clustering. We can also see the different parameter combinations that went into the consensus clustering by using `getClusterManyParams` (here only 2 different parameters).

```
head(getClusterManyParams(rsecFluidigm))
```

```
##                clusteringIndex k alpha
## k0=4,alpha=0.1               3 4   0.1
## k0=5,alpha=0.1               4 5   0.1
## k0=6,alpha=0.1               5 6   0.1
## k0=7,alpha=0.1               6 7   0.1
## k0=8,alpha=0.1               7 8   0.1
## k0=9,alpha=0.1               8 9   0.1
```

### 2.3.2 Visualizing the output

`clusterExperiment` also provides many ways to visualize the output of RSEC (or any set of clusterings run in `clusterExperiment`, as we’ll show below).

#### 2.3.2.1 Visualizing many clusterings

The first such useful visualization is a plot of all of the clusterings together using the `plotClusters` command. For this visualization, it is useful to change the amount of space on the left of the plot to allow for the labels of the clusterings, so we will reset the `mar` option in `par`. We also decrease the `axisLine` argument that decides the amount of space between the axis and the labels to give more space to the labels (`axisLine` is passed internally to the `line` option in `axis`).

```
defaultMar<-par("mar")
plotCMar<-c(1.1,8.1,4.1,1.1)
par(mar=plotCMar)
plotClusters(rsecFluidigm,main="Clusters from RSEC", whichClusters="workflow", colData=c("Biological_Condition","Published2"), axisLine=-1)
```

This plot shows the samples in the columns, and different clusterings on the rows. Each sample is color coded based on its clustering for that row, where the colors have been chosen to try to match up clusters across different clusterings that show large overlap. Moreover, the samples have been ordered so that each subsequent clustering (starting at the top and going down) will try to order the samples to keep the clusters together, without rearranging the clustering blocks of the previous clustering/row.

We also added a `colData` argument in our call, indicating that we also want to visualize some information about the samples saved in the `colData` slot (inherited from our original `fluidigm` object). We chose the columns “Biological\_Condition” and “Published2” from `colData`, which correspond to the original biological condition of the experiment, and the clusters reported in the original paper, respectively. The data from `colData` (when requested) are always shown at the bottom of the plot.

Notice that some samples are white. This indicates that they have the value -1, meaning they were not clustered. In fact, for many clusterings, there is a large amount of white here. This is likely do to the fact that there are only 65 cells here, and the default parameters of RSEC are better suited for a large number of cells, such as seen in more modern single-cell sequencing experiments. The sequential clustering may be problematic for small numbers of cells.

We can use an alternative version of `plotClusters` called `plotClustersWorkflow` that will better emphasize the more final clusterings from the ensemble/concensus step and merging steps (it currently does not allow for showing the `colData` as well, however – only clustering results).

```
par(mar=plotCMar)
plotClustersWorkflow(rsecFluidigm)
```

#### 2.3.2.2 Barplots & contingency tables

We can examine size distribution of a single clustering with the function `plotBarplot`. By default, the cluster picked will be the primary cluster.

```
plotBarplot(rsecFluidigm,main=paste("Distribution of samples of",primaryClusterLabel(rsecFluidigm)))
```

We can also pick a particular intermediate clustering, say our intial consensus clustering before merging.

```
plotBarplot(rsecFluidigm,whichClusters=c("makeConsensus" ))
```

We can also compare two specific clusters with a simple barplot using `plotBarplot`. Here we compare the “makeConsensus” and the “mergeClusters” clusterings.

```
plotBarplot(rsecFluidigm,whichClusters=c("mergeClusters" ,"makeConsensus"))
```

Since “makeConsensus” is a partition of “mergeClusters”, there is perfect subsetting within the clusters of “mergeClusters”.

A related plot is to plot a heatmap of the contingency table between two clusterings provided by `plotClustersTable`. This function plots a heatmap of the results of `tableClusters`, optionally converting them to proportions using `prop.table` function based on the parameter `margin`. Here, we’ll set `margin=1`, meaning we will show each row (corresponding to a cluster of the `mergeCluster` clustering), as a proportion – i.e. how the samples in that row’s cluster are distributed across the clusters of the other clustering, `makeConsensus`

```
plotClustersTable(rsecFluidigm,whichClusters=c("mergeClusters" ,"makeConsensus"), margin=1)
```

Again, since `makeConsensus` clusters are all completely contained in `mergeClusters`, this plot has less information than if we were comparing competing clusterings (e.g. different results from `mergingClusters`, see below). For example, there is nothing on the off-diagonal. But we can still see about how the smaller `makeConsensus` make up the `mergeClusters`.

#### 2.3.2.3 Co-Clustering

We can also visualize the proportion of times samples were together in the individual clusterings (i.e. before the consensus clustering):

```
plotCoClustering(rsecFluidigm,whichClusters=c("mergeClusters","makeConsensus"))
```

Note that this is not the result from any particular subsampling (which was done repeatedly for each clustering, and those many matrices are not stored), but rather the proportion of times across the final results of the clusterings we ran. The initial consensus clustering in `makeConsensus` was made based on these proportions and a particular cutoff of the required proportion of times the samples needed to be together.

#### 2.3.2.4 Plot of Hierarchy of Clusters

We can visualize how the initial ensembl cluster in `makeConsensus` was clustered into a hierarchy and merged to give us the final clustering in `mergeClusters`:

```
plotDMar<-c(8.1,1.1,5.1,8.1)
par(mar=plotDMar)
plotDendrogram(rsecFluidigm,whichClusters=c("makeConsensus","mergeClusters"))
```

As shown in this plot, the individual clusters of the `makeConsensus` ensembl clustering were hierarchically clustered (hence the note that the dendrogram was made from the `makeConsensus` clustering), and similar sister clusters were merged if there were not enough gene differences between them.

#### 2.3.2.5 2D plot of clusters

Finally, we can plot a 2-dimensional representation of the data with PCA and color code the samples to get a sense of how the data cluster.

```
plotReducedDims(rsecFluidigm)
```

We can also look at a higher number of dimensions (or different dimensions) by changing the parameter ‘whichDims’.

```
plotReducedDims(rsecFluidigm,whichDims=c(1:4))
```

In this case we can see that higher dimensions show us a greater amount of separation between the clusters than in just 2 dimensions.

## 2.4 Rerunning RSEC with different parameters

In the next section, we will describe more about the options we could adjust in `RSEC`. As an example of a few options, we might, for example, want to change the proportion of co-clustering we required in making our `makeConsensus` clustering (which used the default of 0.7), or change the proportion of genes that must show differences in order to not merge clusters or the method of deciding. We can call `RSEC` again on our object `rsecFluidigm` and it will not redo the many individual clustering steps which are time intensive (unless we request it to rerun it by including the argument `rerunClusterMany=TRUE`). We demonstrate this in our next command where we change these choices in the following ways:

- set the proportion of co-clustering required by the argument `consensusProportion=0.6`,
- make the merge cutoff `mergeCutoff=0.01`
- decide to use a different method of estimating the proportion differential for merge by setting `mergeMethod="Storey"` instead of the default (“adjP”).
- no longer adjust the minimum cluster size and use the default (`consensusMinSize=5`).

These are the main parameters we might frequently want to tweak in `RSEC`.

```
rsecFluidigm<-RSEC(rsecFluidigm,isCount=TRUE,consensusProportion=0.6,mergeMethod="JC",mergeCutoff=0.05)
```

Notice that we save the output over our original object. This is the standard way to work with the `ClusterExperiment` objects, since the package’s commands just continues to add the clusterings, without deleting anything from before. In this way, we do not duplicate the actual data in our workspace (which is often large).

We can compare the results of our changes with the `whichClusters` command to explicitly choose the clusterings we want to plot:

```
defaultMar<-par("mar")
plotCMar<-c(1.1,8.1,4.1,1.1)
par(mar=plotCMar)
plotClusters(rsecFluidigm,main="Clusters from RSEC", whichClusters=c("mergeClusters.1","makeConsensus.1","mergeClusters","makeConsensus"), colData=c("Biological_Condition","Published2"), axisLine=-1)
```

The clusterings with the `.1` appended to the labels are the previous `makeConsensus` and `mergeClusters` clusterings from the default setting (see Rerunning to see how different versions are labeled and stored internally). We can see that we lost several clusters with these options.

In what follows, we’ll go back to the original (default) RSEC settings by rerunning RSEC (the original clusters are saved in the `rsecFluidigm` object, but there is useful information about the merging that is overwritten by our latest call so we will just rerun it to recreate the clustering). Again, we will set a lot of parameters to keep the object the same as before:

```
rsecFluidigm<-RSEC(rsecFluidigm,
    isCount=TRUE,
 consensusMinSize=3,
 consensusProportion=0.7,
 dendroReduce= "mad",
 dendroNDims=1000,
 mergeMethod="adjP",
 mergeCutoff=0.03
 )
par(mar=plotCMar)
plotClusters(rsecFluidigm,main="Clusters from RSEC", whichClusters=c("mergeClusters","makeConsensus"), colData=c("Biological_Condition","Published2"), axisLine=-1)
```

In practice, it can be useful to interactively make choices about these parameters by rerunning each the individual steps of the workflow separately and visualizing the changes before moving to the next step, as we do below during our overview of the steps.

## 2.5 Finding Features related to the clusters

A common practice after determining a set of clusters is to perform differential gene expression analysis in order to find genes that show the greatest differences amongst the clusters. We would stress that this is purely an exploratory technique, and any p-values that result from this analysis are not valid, in the sense that they are likely to be inflated. This is because the same data was used to define the clusters and to perform differential expression analysis.

Since this is a common task, we provide the function `getBestFeatures` to perform various kinds of differential expression analysis between the clusters. A common F-statistic between groups can be chosen. However, we find that it is far more informative to do pairwise comparisons between clusters, or one cluster against all, in order to find genes that are specific to a particular cluster. An option for all of these choices is provided in the `getBestFeatures` function.

The `getBestFeatures` function uses the DE analysis provided by the `limma` package (Smyth 2004, Ritchie et al. (2015)) or `edgeR` package (Robinson, Mccarthy, and Smyth 2010). In addition, the `getBestFeatures` function provides an option to do use the “voom” correction in the `limma` package (Law et al. 2014) to account for the mean-variance relationship that is common in count data. The tests performed by `getBestFeatures` are specific contrasts between clustering groups; these contrasts can be retrieved without performing the tests using `clusterContrasts`, including in a format appropriate for the `MAST` algorithm.

As mentioned above, there are several types of tests that can be performed to identify features that are different between the clusters which we describe in the section entitled Finding Features related to a Clustering. Here we simply perform all pairwise tests between the clusters.

```
pairsAllRSEC<-getBestFeatures(rsecFluidigm,contrastType="Pairs",p.value=0.05,
                          number=nrow(rsecFluidigm),DEMethod="edgeR")
head(pairsAllRSEC)
```

```
##   IndexInOriginal  Contrast Feature     logFC   logCPM        LR
## 1            1465 Cl01-Cl02    DLK1  13.90356 8.804668 172.58983
## 2            5044 Cl01-Cl02  RPS4Y1  12.51876 7.424298 119.76806
## 3            2291 Cl01-Cl02    GPC3  12.21939 7.126492 110.84635
## 4            3788 Cl01-Cl02    NNAT -13.34129 8.751824 110.37000
## 5            3985 Cl01-Cl02    OTX2  11.80412 6.714252  87.07782
## 6            1640 Cl01-Cl02   EFNA5  11.35424 8.430487  84.19065
##        P.Value    adj.P.Val
## 1 2.011624e-39 1.422017e-34
## 2 7.110712e-28 2.513281e-23
## 3 6.393907e-26 9.579242e-22
## 4 8.130634e-26 9.579242e-22
## 5 1.043343e-20 9.219237e-17
## 6 4.492896e-20 3.528920e-16
```

We can visualize only these significantly different pair-wise features with `plotHeatmap` by using the column “IndexInOriginal” in the result of `getBestFeatures` to quickly identify the genes to be used in the heatmap. Notice that the same genes can be replicated across different contrasts, so we will not always have unique genes:

```
length(pairsAllRSEC$Feature)==length(unique(pairsAllRSEC$Feature))
```

```
## [1] FALSE
```

In this case they are not unique because the same gene can be significant for different pairs tests. Hence, we will make sure we take only unique gene values so that they are not plotted multiple times in our heatmap. (This is a good practice even if in a particular case the genes are unique).

```
plotHeatmap(rsecFluidigm, whichClusters=c("makeConsensus","mergeClusters"),clusterSamplesData="dendrogramValue",
            clusterFeaturesData=unique(pairsAllRSEC[,"IndexInOriginal"]),
            main="Heatmap of features w/ significant pairwise differences",
            breaks=.99)
```

Notice that the samples clustered into the `-1` cluster (i.e. not assigned) are clustered as an outgroup. This is a choice that is made when the dendrogram (described below). These samples can also be mixed into the dendrogram (see makeDendrogram)

We can identify the genes corresponding to the different contrasts with the `plotContrastHeatmap` function where the genes (rows) are organized by what contrast for which they are significant. The option `nBlankLines` controls the space between the groups of genes from each contrast. We also give the argument `whichCluster="primary"` to indicate that the contrasts were created with the primary clustering – this means that the legend will put in the names of the clusters rather than their (internal) numeric id.

```
plotContrastHeatmap(rsecFluidigm, signif=pairsAllRSEC,nBlankLines=40,whichCluster="primary")
```

# 3 Overview of the clustering workflow

We give an overview here of the steps used by the `RSEC` wrapper and more generally in the `clusterExperiment` package. The section The clustering workflow goes over these steps and the possible arguments in greater details.

The standard clustering workflow steps are the following:

- `clusterMany` – run desired clusterings
- `makeConsensus` – get a unified clustering
- `makeDendrogram` – get a hierarchical relationship between the clusters
- `mergeClusters` – merge together clusters with little DE between their genes.

These clustering steps are done in one function call by `RSEC`, described above, which is most straightforward usage. However, to understand the parameters available in `RSEC` it is useful to go through the steps individually. Furthermore `RSEC` has streamlined the workflow to concentrate on using the workflow with subsampling and sequential strategies, but going through the individual steps demonstrates how the user can make different choices.

Therefore in this section, we will go through these steps, but instead of using the parameters of `RSEC` that involve subsampling and are more computationally intensive, we will run through the same steps, only using just basic PAM clustering with no subsampling or sequential clustering. This is simply for the purpose of briefly understanding the intermediate steps that `RSEC` follows. Later sections will go through these steps in more detail and discuss the particular choices embedded in `RSEC`.

## 3.1 Step 1: Clustering with `clusterMany`

`clusterMany` lets the user quickly pick between many clustering options and run all of the clusterings in one single command. In the quick start we pick a simple set of clusterings based on varying the dimensionality reduction options. The way to designate which options to vary is to give multiple values to an argument.

Here is our call to `clusterMany`:

```
ce<-clusterMany(se, clusterFunction="pam",ks=5:10, minSizes=5,
      isCount=TRUE,reduceMethod=c("PCA","var"),nFilterDims=c(100,500,1000),
      nReducedDims=c(5,15,50),run=TRUE)
```

In this call to `clusterMany` we made the follow choices about what to vary:

- set `reduceMethod=c("PCA", "var")` meaning run the clustering algorithm trying two different methods for dimensionality reduction: the top principal components and filtering to the top most variable genes
- For PCA reduction, choose 5,15, and 50 principal components for the reduced data set (set `nReducedDims=c(5,15,50)`)
- For most variable genes reduction, we choose 100, 500, and 1000 most variable genes (set `nFilterDims=c(100,500,1000)`)
- For the number of clusters, vary from \(k=5,\ldots,10\) (set `ks=5:10`)

By giving only a single value to the relative argument, we keep the other possible options fixed, for example:

- we used ‘pam’ for all clustering (`clusterFunction="pam"`)
- we set `minSizes=5`. This is an argument that allows the user to set a minimum required size and clusters of size less than that value will be ignored and samples assigned to them given the unassigned value of -1. The default of `1` would mean that this option is not used.

We also set `isCount=TRUE` to indicate that our input data are counts. This means that operations for clustering and visualizations will internally transform the data as \(log\_2(x+1)\) (We could have alternatively explicitly set a transformation by giving a function to the `transFun` argument, for example if we wanted \(\sqrt(x)\) or \(log(x+\epsilon)\) or just `identity`).

We can visualize the resulting clusterings using the `plotClusters` command as we did for the `RSEC` results.

```
defaultMar<-par("mar")
par(mar=plotCMar)
plotClusters(ce,main="Clusters from clusterMany", whichClusters="workflow", colData=c("Biological_Condition","Published2"), axisLine=-1)
```

```
ce<-clusterMany(se, clusterFunction="pam",ks=5:10, minSizes=5,
      isCount=TRUE,reduceMethod=c("PCA","var"),nFilterDims=c(100,500,1000),
      nReducedDims=c(5,15,50),run=TRUE)
test<-getClusterManyParams(ce)
ord<-order(test[,"k"],test[,"nFilterDims"],test[,"nReducedDims"])
plotClusters(ce,main="Clusters from clusterMany", whichClusters=test$clusteringIndex[ord], colData=c("Biological_Condition","Published2"), axisLine=-1)
```

We can see that some clusters are fairly stable across different choices of dimensions while others can vary dramatically.

Notice that again some samples are white (i.e the value -1, meaning they were not clustered). This is from our choices to require at least 5 samples to make a cluster.

We have set `whichClusters="workflow"` to only plot clusters created from the workflow. Right now that’s all there are anyway, but as commands get rerun with different options, other clusterings can build up in the object (see discussion in this section about how multiple calls to workflow are stored). So setting `whichClusters="workflow"` means that we are only going to see our most recent calls to the workflow (so far we only have the 1 step, which is the `clusterMany` step). We seen already that `whichClusters` can be set to limit to specific clusterings or specific steps in the workflow .

We cal also give to the `whichClusters` an argument indices of clusters stored in the `ClusterExperiment` object, which can allow us to show the clusters in a different order. Here we’ll pick an order which corresponds to varying the number of dimensions, rather than k. We can find the parameters for each clustering using the `getClusterManyParams`

```
cmParams<-getClusterManyParams(ce)
head(cmParams)
```

```
##                                                       clusteringIndex
## reduceMethod=PCA,nReducedDims=5,nFilterDims=NA,k=5                  1
## reduceMethod=var,nReducedDims=NA,nFilterDims=100,k=5                2
## reduceMethod=PCA,nReducedDims=15,nFilterDims=NA,k=5                 3
## reduceMethod=PCA,nReducedDims=50,nFilterDims=NA,k=5                 4
## reduceMethod=var,nReducedDims=NA,nFilterDims=500,k=5                5
## reduceMethod=var,nReducedDims=NA,nFilterDims=1000,k=5               6
##                                                       reduceMethod
## reduceMethod=PCA,nReducedDims=5,nFilterDims=NA,k=5             PCA
## reduceMethod=var,nReducedDims=NA,nFilterDims=100,k=5           var
## reduceMethod=PCA,nReducedDims=15,nFilterDims=NA,k=5            PCA
## reduceMethod=PCA,nReducedDims=50,nFilterDims=NA,k=5            PCA
## reduceMethod=var,nReducedDims=NA,nFilterDims=500,k=5           var
## reduceMethod=var,nReducedDims=NA,nFilterDims=1000,k=5          var
##                                                       nReducedDims
## reduceMethod=PCA,nReducedDims=5,nFilterDims=NA,k=5               5
## reduceMethod=var,nReducedDims=NA,nFilterDims=100,k=5            NA
## reduceMethod=PCA,nReducedDims=15,nFilterDims=NA,k=5             15
## reduceMethod=PCA,nReducedDims=50,nFilterDims=NA,k=5             50
## reduceMethod=var,nReducedDims=NA,nFilterDims=500,k=5            NA
## reduceMethod=var,nReducedDims=NA,nFilterDims=1000,k=5           NA
##                                                       nFilterDims k
## reduceMethod=PCA,nReducedDims=5,nFilterDims=NA,k=5             NA 5
## reduceMethod=var,nReducedDims=NA,nFilterDims=100,k=5          100 5
## reduceMethod=PCA,nReducedDims=15,nFilterDims=NA,k=5            NA 5
## reduceMethod=PCA,nReducedDims=50,nFilterDims=NA,k=5            NA 5
## reduceMethod=var,nReducedDims=NA,nFilterDims=500,k=5          500 5
## reduceMethod=var,nReducedDims=NA,nFilterDims=1000,k=5        1000 5
```

`getClusterManyParams` returns the parameter values, as well as the index of the corresponding clustering (i.e. what column it is in the matrix of clusterings). It is important to note that the index will change if we add additional clusterings, as we will do later.

We will set an order with first the PCA, ordered by number of dimensions, then the Var, ordered by number of diminsions

```
ord<-order(cmParams[,"reduceMethod"],cmParams[,"nReducedDims"])
ind<-cmParams[ord,"clusteringIndex"]
par(mar=plotCMar)
plotClusters(ce,main="Clusters from clusterMany", whichClusters=ind, colData=c("Biological_Condition","Published2"), axisLine=-1)
```

We see that the order in which the clusters are given to `plotClusters` changes the plot greatly. The labels shown are those given automatically by `clusterMany` but can be a bit much for plotting. We choose to remove “Features” as being too wordy:

```
clOrig<-clusterLabels(ce)
clOrig<-gsub("Features","",clOrig)
par(mar=plotCMar)
plotClusters(ce,main="Clusters from clusterMany", whichClusters=ind, clusterLabels=clOrig[ind], colData=c("Biological_Condition","Published2"), axisLine=-1)
```

We could also permanently assign new labels in our `ClusterExperiment` object if we prefer, for example to be more succinct, by changing the `clusterLabels` of the object.

There are many different options for how to run `plotClusters` discussed in in the detailed section on plotClusters, but for now, this plot is good enough for a quick visualization.

## 3.2 Step 2: Find a consensus with `makeConsensus`

To find a consensus clustering across the many different clusterings created by `clusterMany` the function `makeConsensus` can be used next.

```
ce<-makeConsensus(ce,proportion=1)
```

The `proportion` argument indicates the minimum proportion of times samples should be with other samples in the cluster they are assigned to in order to be clustered together in the final assignment. Notice we get a warning that we did not specify any clusters to combine, so it is using the default – those from the `clusterMany` call.

If we look at the `clusterMatrix` of the returned `ce` object, we see that the new cluster from `makeConsensus` has been added to the existing clusterings. This is the basic strategy of all of these functions in this package. Any clustering function that is applied to an existing `ClusterExperiment` object adds the new clustering to the set of existing clusterings, so the user does not need to keep track of past clusterings and can easily compare what has changed.

We can again run `plotClusters`, which will now also show the result of `makeConsensus`. Instead, we’ll use `plotClustersWorkflow` which is nicer for looking specifically at the results of `makeConsensus`

```
head(clusterMatrix(ce)[,1:3])
```

```
##            makeConsensus reduceMethod=PCA,nReducedDims=5,nFilterDims=NA,k=5
## SRR1275356            -1                                                  4
## SRR1275251            -1                                                  5
## SRR1275287            -1                                                  1
## SRR1275364            -1                                                  4
## SRR1275269            -1                                                  1
## SRR1275263            -1                                                  5
##            reduceMethod=var,nReducedDims=NA,nFilterDims=100,k=5
## SRR1275356                                                    1
## SRR1275251                                                    3
## SRR1275287                                                    4
## SRR1275364                                                    1
## SRR1275269                                                    2
## SRR1275263                                                    1
```

```
par(mar=plotCMar)
plotClustersWorkflow(ce)
```

The choices of `proportion=1` in `makeConsensus` is not usually a great choice, and certainly isn’t helpful here. The clustering from the default `makeConsensus` leaves most samples unassigned (white in the above plot). This is because we requires samples to be in the same cluster in *every clustering* in order to be assigned to a cluster together. This is quite stringent. We can vary this by setting the `proportion` argument to be lower. Explicit details on how `makeConsensus` makes these clusters are discussed in the section on makeConsensus.

So let’s label the one we found as “makeConsensus,1” and then create a new one. (Making or changing the label to an informative label will make it easier to keep track of this particular clustering later, particularly if we make multiple calls to the workflow).

```
wh<-which(clusterLabels(ce)=="makeConsensus")
if(length(wh)!=1) stop() else clusterLabels(ce)[wh]<-"makeConsensus,1"
```

Now we’ll rerun `makeConsensus` with `proportion=0.7`. This time, we will give it an informative label upfront in our call to `makeConsensus`.

```
ce<-makeConsensus(ce,proportion=0.7,clusterLabel="makeConsensus,0.7")
par(mar=plotCMar)
plotClustersWorkflow(ce)
```

We see that more clusters are detected. Those that are still not assigned a cluster from `makeConsensus` clearly vary across the clusterings as to whether the samples are clustered together or not. Varying the `proportion` argument will adjust whether some of the unclustered samples get added to a cluster. There is also a `minSize` parameter for `makeConsensus`, with the default of `minSize=5`. We could reduce that requirement as well and more of the unclustered samples would be grouped into a cluster. Here, we reduce it to `minSize=3` (we’ll call this “makeConsensus,final”). We’ll also choose to show all of the different makeConsensus results in our call to `plotClustersWorkflow`:

```
ce<-makeConsensus(ce,proportion=0.7,minSize=3,clusterLabel="makeConsensus,final")
par(mar=plotCMar)
plotClustersWorkflow(ce,whichClusters=c("makeConsensus,final","makeConsensus,0.7","makeConsensus,1"),main="Min. Size=3")
```

As we did before for `RSEC` results, we can also visualize the proportion of times these clusters were together across these clusterings (this information was made and stored in the ClusterExperiment object when we called makeConsensus provided that proportion argument is <1):

```
plotCoClustering(ce)
```

This visualization can help in determining whether to change the value of `proportion` (though see makeConsensus for how -1 assignments affect `makeConsensus`).

## 3.3 Step 3: Merge clusters together with `makeDendrogram` and `mergeClusters`

Once you start varying the parameters, is not uncommon in practice to create forty or more clusterings with `clusterMany`. In which case the results of `makeConsensus` can often result in too many small clusters. We might wonder if they are necessary or could be logically combined together. We could change the value of `proportion` in our call to `makeConsensus`. But we have found that it is often after looking at the clusters, for example with a heatmap, and how different they look on individual genes that we best make this determination, rather than the proportion of times they are together in different clustering routines.

For this reason, we often find the need for an additional clustering step that merges clusters together that are not different, based on running tests of differential expression between the clusters found in `makeConsensus`. This is done by the function `mergeClusters`. We often display and use both sets of clusters side-by-side (that from `makeConsensus` and that from `mergeClusters`).

`mergeClusters` needs a hierarchical clustering of the clusters in order to merge clusters; it then goes progressively up that hierarchy, deciding whether two adjacent clusters can be merged. The function `makeDendrogram` makes such a hierarchy between clusters (by applying `hclust` to the medoids of the clusters). Because the results of `mergeClusters` are so dependent on that hierarchy, we require the user to call `makeDendrogram` rather than calling it automatically internally. This is because different options in `makeDendrogram` can affect how the clusters are hierarchically ordered, and we want to encourage the user make these choices.

As an example, here we use the 500 most variable genes to make the cluster hierarchy (note we can make different choices here than we did in the clustering).

```
ce<-makeDendrogram(ce,reduceMethod="var",nDims=500)
plotDendrogram(ce)
```

Notice that the relative sizes of the clusters are shown as well.

We can see that clusters 1 and 3 are most closely related, at least in the top 500 most variable genes.

Notice I don’t need to make the dendrogram again, because it’s saved in `ce`. If we look at the summary of `ce`, it now has ‘makeDendrogram’ marked as ‘Yes’.

```
ce
```

```
## class: ClusterExperiment 
## dim: 7069 65 
## reducedDimNames: PCA 
## filterStats: var var_makeConsensus.final 
## -----------
## Primary cluster type: makeConsensus 
## Primary cluster label: makeConsensus,final 
## Table of clusters (of primary clustering):
## -1 c1 c2 c3 c4 c5 c6 c7 
##  6 15 14  9  8  5  4  4 
## Total number of clusterings: 39 
## Dendrogram run on 'makeConsensus,final' (cluster index: 1)
## -----------
## Workflow progress:
## clusterMany run? Yes 
## makeConsensus run? Yes 
## makeDendrogram run? Yes 
## mergeClusters run? No
```

Now we are ready to actually merge clusters together. We run `mergeClusters` that will go up this hierarchy and compare the level of differential expression (DE) in each pair. In other words, if we focus on the left side of the tree, DE tests are run, between 1 and 3, and between 6 and 8. If there is not enough DE between each of these (based on a cutoff that can be set by the user), then clusters 1 and 3 and/or 6 and 8 will be merged. And so on up the tree.

If the dataset it not too large, is can be useful to first run `mergeClusters` without actually saving the results so as to preview what the final clustering will be (and perhaps to help in setting the cutoff).

```
mergeClusters(ce,mergeMethod="adjP",DEMethod="edgeR",plotInfo="mergeMethod")
```

Notice that unlike our `RSEC` calls, we have to explicitly choose the DE method that is used in our call to `mergeClusters`. `RSEC` chooses the method by default based on the value of `isCount` argument (but the user can set it in `RSEC` with `mergeDEMethod` argument). Since our data is counts, we choose the DE method to be `edgeR` (which is also what `RSEC` chooses by default since we set `isCount=TRUE`).

The default cutoff is `cutoff=0.1`, meaning those nodes with less than 10% of genes estimated to be differentially expressed between its two children groupings of samples are merged. This is pretty stringent, and as we see it results in no clusterings being kept.

However, the plot tells us the estimate of that proportion for each node. We can decide on a better cutoff using that information. We choose instead `cutoff=0.01`:

```
ce<-mergeClusters(ce,mergeMethod="adjP",DEMethod="edgeR",cutoff=0.05)
```

Notice that now the plot has given the estimates from *all* of the methods (the default set by `plotInfo` argument), not just the `adjP` method. But the dotted lines of the dendrogram indicate the merging performed by the actual choices in merging made in the command (`mergeMethod="adjP"` and `cutoff=0.01`).

It can be interesting to visualize the clusterings both with the `plotClustersWorkflow` and the co-Proportion plot (a heatmap of the proportion of times the samples co-clustered):

```
par(mar=plotCMar)
plotClustersWorkflow(ce,whichClusters="workflow") 
plotCoClustering(ce,whichClusters=c("mergeClusters","makeConsensus"),
                 colData=c("Biological_Condition","Published2"),annLegend=FALSE)
```

Notice that `mergeClusters` combines clusters based on the actual values of the features, while the `coClustering` plot shows how often the samples clustered together. It is not uncommon that `mergeClusters` will merge clusters that don’t look “close” on the `coClustering` plot. This can be due to just the choices of the hierarchical clustering between the clusters, but can also be because the two merged clusters are not often confused for each other across the clustering algorithms, yet still don’t have strong differences on individual genes. This can be the case especially when the clustering is done on reduced PCA space, where an accumulation of small differences might consistently separate the samples (so that comparatively few clusterings are “confused” as to the samples). But because the differences are not strong on individual genes, `mergeClusters` combines them. These are ultimately different criteria.

Finally, we can do a heatmap visualizing this final step of clustering.

```
plotHeatmap(ce,clusterSamplesData="dendrogramValue",breaks=.99,
            colData=c("Biological_Condition", "Published1", "Published2"))
```

By choosing “dendrogramValue” for the clustering of the samples, we will be showing the clusters according to the hierarchical ordering of the clusters found by `makeDendrogram`. The argument `breaks=0.99` means that the last color of the heatmap spectrum will be forced to be the top 1% of the data (rather than evenly spaced through the entire range of values). This can be helpful in making sure that rare extreme values in the upper range do not absorb too much space in the color spectrum. There are many more options for `plotHeatmap`, some of which are discussed in the section on plotHeatmap.

## 3.4 RSEC

The above explanation follows the simple example of PAM. The original RSEC result called `RSEC` which calls these steps internally. Many of the options described above can be set through a call to RSEC, but some are restricted for simplicity. A detail explanation of the differences can be found in the section RSEC{#RSEC} below. But briefly, the following RSEC command, which uses most of the arguments of `RSEC`:

```
rsecOut<-RSEC(se, isCount=TRUE, reduceMethod="PCA", nReducedDims=c(50,10), k0s=4:15,
        alphas=c(0.1,0.2,0.3),betas=c(0.8,0.9), minSizes=c(1,5), clusterFunction="hierarchical01",
        consensusProportion=0.7, consensusMinSize=5,
        dendroReduce="mad",dendroNDims=500,
        mergeMethod="adjP",mergeCutoff=0.05,
        )
```

would be equivalent to the following individual steps:

```
ce<-clusterMany(se,ks=4:15,alphas=c(0.1,0.2,0.3),betas=c(0.8,0.9),minSizes=c(1,5),
    clusterFunction="hierarchical01", sequential=TRUE,subsample=TRUE,
         reduceMethod="PCA",nFilterDims=c(50,10),isCount=TRUE)
ce<-makeConsensus(ce, proportion=0.7, minSize=5)
ce<-makeDendrogram(ce,reduceMethod="mad",nDims=500)
ce<-mergeClusters(ce,mergeMethod="adjP",DEMethod="edgeR",cutoff=0.05,plot=FALSE)
```

Note that this mean the `RSEC` function always calls sequential and subsampling.

# 4 ClusterExperiment Objects

The `ce` object that we created by calling `clusterMany` is a `ClusterExperiment` object. The `ClusterExperiment` class is used by this package to keep the data and the clusterings together. It inherits from `SingleCellExperiment` class (which inherits from `SummarizedExperiment`) which means the data and `colData` and other information orginally in the `fluidigm` object are retained and can be accessed with the same functions as before. The `ClusterExperiment` object additionally stores clusterings and information about the clusterings along side the data. This helps keep everything together, and like the original `SummarizedExperiment` object we started with, allows for simple things like subsetting to a reduced set of subjects and being confident that the corresponding clusterings, colData, and so forth are similarly subset.

Typing the name at the control prompt results in a quick summary of the object.

```
ce
```

```
## class: ClusterExperiment 
## dim: 7069 65 
## reducedDimNames: PCA 
## filterStats: var var_makeConsensus.final 
## -----------
## Primary cluster type: mergeClusters 
## Primary cluster label: mergeClusters 
## Table of clusters (of primary clustering):
## -1 m1 m2 m3 m4 
##  6 15 14 13 17 
## Total number of clusterings: 40 
## Dendrogram run on 'makeConsensus,final' (cluster index: 2)
## -----------
## Workflow progress:
## clusterMany run? Yes 
## makeConsensus run? Yes 
## makeDendrogram run? Yes 
## mergeClusters run? Yes
```

This summary tells us the total number of clusterings (40), and gives some indication as to what parts of the standard workflow have been completed and stored in this object. It also gives information regarding the `primaryCluster` of the object. The `primaryCluster` is just one of the clusterings that has been chosen to be the “primary” clustering, meaning that by default various functions will turn to this clustering as the desired clustering to use. `clusterMany` arbitrarily sets the ‘primaryCluster’ to the first one, and each later step of the workflow sets the primary index to the most recent, but the user can set a specific clustering to be the primaryCluster with `primaryClusterIndex`. Often, if a function is not given a specific clustering (usually via an option `whichCluster` or `whichClusters`) the “primary” cluster is taken by default.

There are also additional commands to access the clusterings and their related information (type `help("ClusterExperiment-methods")` for more).

The cluster assignments are stored in the `clusterMatrix` slot of `ce`, with samples on the rows and different clusterings on the columns. We saw that we can look at the cluster matrix and the primary cluster with the commands `clusterMatrix` and `primaryCluster`

```
head(clusterMatrix(ce))[,1:5]
```

```
##            mergeClusters makeConsensus,final makeConsensus,0.7
## SRR1275356            -1                  -1                -1
## SRR1275251            -1                  -1                -1
## SRR1275287             3                   6                -1
## SRR1275364             4                   4                 4
## SRR1275269             3                   6                -1
## SRR1275263             4                   5                 5
##            makeConsensus,1
## SRR1275356              -1
## SRR1275251              -1
## SRR1275287              -1
## SRR1275364              -1
## SRR1275269              -1
## SRR1275263              -1
##            reduceMethod=PCA,nReducedDims=5,nFilterDims=NA,k=5
## SRR1275356                                                  4
## SRR1275251                                                  5
## SRR1275287                                                  1
## SRR1275364                                                  4
## SRR1275269                                                  1
## SRR1275263                                                  5
```

```
primaryCluster(ce)
```

```
##  [1] -1 -1  3  4  3  4  3  1  1  1  2  2  4  2  3  4  3  1  3  3  2  2  2  1
## [25] -1  1  1  1  4  2  2  4  4  3  3 -1  3  2 -1  4  3  1  1  4  1  1  4 -1
## [49]  3  2  2  2  1  2  3  4  4  1  1  4  4  4  2  4  4
```

Remember that we made multiple calls to `makeConsensus`: only the last such call will be shown when we use `whichClusters="workflow"` in our plotting (see this section for a discussion of how these repeated calls are handled.)

`clusterLabels` gives the column names of the `clusterMatrix`; `clusterMany` has given column names based on the parameter choices, and later steps in the workflow also give a name (or allow the user to set them).

```
head(clusterLabels(ce),10)
```

```
##  [1] "mergeClusters"                                        
##  [2] "makeConsensus,final"                                  
##  [3] "makeConsensus,0.7"                                    
##  [4] "makeConsensus,1"                                      
##  [5] "reduceMethod=PCA,nReducedDims=5,nFilterDims=NA,k=5"   
##  [6] "reduceMethod=var,nReducedDims=NA,nFilterDims=100,k=5" 
##  [7] "reduceMethod=PCA,nReducedDims=15,nFilterDims=NA,k=5"  
##  [8] "reduceMethod=PCA,nReducedDims=50,nFilterDims=NA,k=5"  
##  [9] "reduceMethod=var,nReducedDims=NA,nFilterDims=500,k=5" 
## [10] "reduceMethod=var,nReducedDims=NA,nFilterDims=1000,k=5"
```

As we’ve seen, the user can also change these labels.

`clusterTypes` on the other hand indicates what call made the clustering. Unlike the labels, it is wise to not change the values of `clusterTypes` unless you are sure of what you are doing because these values are used to identify clusterings from different steps of the workflow.

```
head(clusterTypes(ce),10)
```

```
##  [1] "mergeClusters"   "makeConsensus"   "makeConsensus.2" "makeConsensus.1"
##  [5] "clusterMany"     "clusterMany"     "clusterMany"     "clusterMany"    
##  [9] "clusterMany"     "clusterMany"
```

The information that was in the original `fluidigm` object has also been preserved, like `colData` that contains information on each sample.

```
colData(ce)[,1:5]
```

```
## DataFrame with 65 rows and 5 columns
##               NREADS  NALIGNED    RALIGN TOTAL_DUP    PRIMER
##            <numeric> <numeric> <numeric> <numeric> <numeric>
## SRR1275356  10554900   7555880   71.5862   58.4931 0.0217638
## SRR1275251   8524470   5858130   68.7213   65.0428 0.0351827
## SRR1275287   7229920   5891540   81.4884   49.7609 0.0208685
## SRR1275364   5403640   4482910   82.9609   66.5788 0.0298284
## SRR1275269  10729700   7806230   72.7536   50.4285 0.0204349
## ...              ...       ...       ...       ...       ...
## SRR1275259   5949930   4181040   70.2705   52.5975 0.0205253
## SRR1275253  10319900   7458710   72.2747   54.9637 0.0205342
## SRR1275285   5300270   4276650   80.6873   41.6394 0.0227383
## SRR1275366   7701320   6373600     82.76   68.9431 0.0266275
## SRR1275261  13425000   9554960   71.1727   62.0001 0.0200522
```

Another important slot in the `ClusterExperiment` object is the `clusterLegend` slot. This consists of a list, one element per column (or clustering) of `clusterMatrix`, that gives colors and names to each cluster within a clustering.

```
length(clusterLegend(ce))
```

```
## [1] 40
```

```
clusterLegend(ce)[1:2]
```

```
## $mergeClusters
##      clusterIds color     name
## [1,] "-1"       "white"   "-1"
## [2,] "1"        "#1F78B4" "m1"
## [3,] "2"        "#33A02C" "m2"
## [4,] "3"        "#FF7F00" "m3"
## [5,] "4"        "#6A3D9A" "m4"
## 
## $`makeConsensus,final`
##      clusterIds color     name
## [1,] "-1"       "white"   "-1"
## [2,] "1"        "#1F78B4" "c1"
## [3,] "2"        "#33A02C" "c2"
## [4,] "3"        "#FF7F00" "c3"
## [5,] "4"        "#6A3D9A" "c4"
## [6,] "5"        "#B15928" "c5"
## [7,] "6"        "#A6CEE3" "c6"
## [8,] "7"        "#bd18ea" "c7"
```

We can see that each element of `clusterLegend` consists of a matrix, with number of rows equal to the number of clusters in the clustering. The columns store information about that cluster. `clusterIds` is the internal id (integer) used in `clusterMatrix` to identify the cluster, `name` is a name for the cluster, and `color` is a color for that cluster. `color` is used in plotting and visualizing the clusters, and `name` is an arbitrary character string for a cluster. They are automatically given default values when the `ClusterExperiment` object is created, but we will see under the description of visualization methods how the user might want to manipulate these for better plotting results.

We can assign new values with a simple assignment operator, but we also provide the functions `renameClusters` and `recolorClusters` to help do this. Here we change the internal cluster names of the first clustering from lowercase to uppercase “M” using the function `renameClusters`:

```
newName<-gsub("m","M",clusterLegend(ce)[[1]][,"name"])
renameClusters(ce,whichCluster=1,value=newName)
```

```
## class: ClusterExperiment 
## dim: 7069 65 
## reducedDimNames: PCA 
## filterStats: var var_makeConsensus.final 
## -----------
## Primary cluster type: mergeClusters 
## Primary cluster label: mergeClusters 
## Table of clusters (of primary clustering):
## -1 M1 M2 M3 M4 
##  6 15 14 13 17 
## Total number of clusterings: 40 
## Dendrogram run on 'makeConsensus,final' (cluster index: 2)
## -----------
## Workflow progress:
## clusterMany run? Yes 
## makeConsensus run? Yes 
## makeDendrogram run? Yes 
## mergeClusters run? Yes
```

```
print(ce)
```

```
## class: ClusterExperiment 
## dim: 7069 65 
## reducedDimNames: PCA 
## filterStats: var var_makeConsensus.final 
## -----------
## Primary cluster type: mergeClusters 
## Primary cluster label: mergeClusters 
## Table of clusters (of primary clustering):
## -1 m1 m2 m3 m4 
##  6 15 14 13 17 
## Total number of clusterings: 40 
## Dendrogram run on 'makeConsensus,final' (cluster index: 2)
## -----------
## Workflow progress:
## clusterMany run? Yes 
## makeConsensus run? Yes 
## makeDendrogram run? Yes 
## mergeClusters run? Yes
```

Note that if you choose to not use these functions and instead replace the whole matrix (e.g. `clusterLegend(ce)[[1]]<- ...`) you should be careful not assign new values to `clusterIds` column, as the entries must exactly correspond to the internal ids of the clustering stored in the clustering matrix.

## 4.1 Subsetting ClusterExperiment objects

Like `SummarizedExperiment` or `SingleCellExperiment` classes, standard subsetting of a `ClusterExeriment` object will result in a new `ClusterExperiment` object with all of the relevant parts of the data similarly subsetted.

```
smallCe<-ce[1:5,1:10]
smallCe
```

```
## class: ClusterExperiment 
## dim: 5 10 
## reducedDimNames: PCA 
## filterStats: var var_makeConsensus.final 
## -----------
## Primary cluster type: mergeClusters 
## Primary cluster label: mergeClusters 
## Table of clusters (of primary clustering):
## -1 m1 m3 m4 
##  2  3  3  2 
## Total number of clusterings: 40 
## No dendrogram present
## -----------
## Workflow progress:
## clusterMany run? Yes 
## makeConsensus run? Yes 
## makeDendrogram run? No 
## mergeClusters run? Yes
```

Notice from looking at the `clusterMatrix` below that the clustering results have been subset and that after subsetting, the internal cluster ids may change (because they are required to be consecutive).

```
clusterMatrix(smallCe)[,1:4]
```

```
##            mergeClusters makeConsensus,final makeConsensus,0.7
## SRR1275356            -1                  -1                -1
## SRR1275251            -1                  -1                -1
## SRR1275287             2                   5                -1
## SRR1275364             3                   3                 3
## SRR1275269             2                   5                -1
## SRR1275263             3                   4                 4
## SRR1275338             2                   2                 2
## SRR1274117             1                   1                 1
## SRR1274089             1                   1                 1
## SRR1274125             1                   1                 1
##            makeConsensus,1
## SRR1275356              -1
## SRR1275251              -1
## SRR1275287              -1
## SRR1275364              -1
## SRR1275269              -1
## SRR1275263              -1
## SRR1275338              -1
## SRR1274117               1
## SRR1274089               1
## SRR1274125               1
```

```
clusterMatrix(ce)[1:10,1:4]
```

```
##            mergeClusters makeConsensus,final makeConsensus,0.7
## SRR1275356            -1                  -1                -1
## SRR1275251            -1                  -1                -1
## SRR1275287             3                   6                -1
## SRR1275364             4                   4                 4
## SRR1275269             3                   6                -1
## SRR1275263             4                   5                 5
## SRR1275338             3                   3                 3
## SRR1274117             1                   1                 1
## SRR1274089             1                   1                 1
## SRR1274125             1                   1                 1
##            makeConsensus,1
## SRR1275356              -1
## SRR1275251              -1
## SRR1275287              -1
## SRR1275364              -1
## SRR1275269              -1
## SRR1275263              -1
## SRR1275338              -1
## SRR1274117               1
## SRR1274089               1
## SRR1274125               1
```

However, the names (and colors) of each cluster should stay the same, which we can see by looking at the `clusterLegend` information

```
clusterLegend(smallCe)[["mergeClusters"]]
```

```
##      clusterIds color     name
## [1,] "-1"       "white"   "-1"
## [2,] "1"        "#1F78B4" "m1"
## [3,] "2"        "#FF7F00" "m3"
## [4,] "3"        "#6A3D9A" "m4"
```

```
clusterLegend(ce)[["mergeClusters"]]
```

```
##      clusterIds color     name
## [1,] "-1"       "white"   "-1"
## [2,] "1"        "#1F78B4" "m1"
## [3,] "2"        "#33A02C" "m2"
## [4,] "3"        "#FF7F00" "m3"
## [5,] "4"        "#6A3D9A" "m4"
```

However subsetting will lose some saved information. In particular, the hierarchy of the clusters that you created with `makeDendrogram` will be deleted in the new object, as will any saved information about the merging in the `mergeClusters` step (since that depended on the dendrogram which is now gone).

```
nodeMergeInfo(ce)
```

```
##    Node                     Contrast isMerged mergeClusterId    Storey
## 1 Node1 (X2+X5+X4+X7)/4-(X1+X3+X6)/3    FALSE             NA 0.4400905
## 2 Node2              X2-(X5+X4+X7)/3    FALSE             NA 0.4058566
## 3 Node3                 X1-(X3+X6)/2    FALSE             NA 0.3526666
## 4 Node4                        X3-X6     TRUE              3 0.2477012
## 5 Node5                 X5-(X4+X7)/2     TRUE              4 0.4115151
## 6 Node6                        X4-X7     TRUE             NA 0.2295940
##          PC       adjP locfdr        MB JC
## 1 0.3780116 0.12137502     NA 0.4041590 NA
## 2 0.3512809 0.09478003     NA 0.3702080 NA
## 3 0.2905322 0.05658509     NA 0.3131985 NA
## 4 0.1886942 0.01938039     NA 0.2138916 NA
## 5 0.3429098 0.03437544     NA 0.3760079 NA
## 6 0.2254791 0.02447305     NA 0.2837742 NA
```

```
nodeMergeInfo(smallCe)
```

```
## NULL
```

The actual clustering created in the `mergeClusters` step, however, are retained as we’ve seen above.

Another useful type of subsetting can be to subset to only samples contained in a set of particular clusters within a clustering. This can be useful, for example, if you want to visualize the data in only those clusters. The function `subsetByCluster` allows you to do this, and it returns a new `ClusterExperiment` object with only those samples. The required input is to identify the values of the clusters you want to keep (by default matching to the clusters’ names)

```
subCe<-subsetByCluster(ce,whichCluster="mergeClusters",clusterValue=c("m1","m2"))
subCe
```

```
## class: ClusterExperiment 
## dim: 7069 29 
## reducedDimNames: PCA 
## filterStats: var var_makeConsensus.final 
## -----------
## Primary cluster type: mergeClusters 
## Primary cluster label: mergeClusters 
## Table of clusters (of primary clustering):
## m1 m2 
## 15 14 
## Total number of clusterings: 40 
## No dendrogram present
## -----------
## Workflow progress:
## clusterMany run? Yes 
## makeConsensus run? Yes 
## makeDendrogram run? No 
## mergeClusters run? Yes
```

The object `subCe` now can be used for visualizing, or any other analysis.

This kind of subsetting can also be useful in comparing clusterings, where the user might want to subset to all of the samples assigned to Cluster 1 in one clustering and then see what clusters that corresponds to in the other clusterings.

## 4.2 Samples not assigned to a cluster (Negative Valued Cluster Assignments)

The different clusters are stored as consecutive integers, with ‘-1’ and ‘-2’ having special meaning.

**Unassigned Samples (-1)** ‘-1’ refers to samples that were not clustered by the clustering algorithm. In our example, we removed clusters that didn’t meet specific size criterion, so they were assigned ‘-1’.

**Missing from Clustering Run (-2)** ‘-2’ is for samples that were not included in the original input to the clustering. This is useful if, for example, you cluster on a subset of the samples, and then want to store this clustering with the clusterings done on all the data. You can create a vector of clusterings that give ‘-2’ to the samples not originally used and then add these clusterings to the `ce` object manually with `addClusters`.

We can also wish to go back and assign these samples to the best cluster possible. This can be done with the `assignUnassigned` function. This function will assign the samples with negative-valued cluster ids to the cluster to which the sample is the closest. Closest is determined by the euclidean distance between an unassigned sample and the median value of the samples assigned to the cluster. The data used by the function to determine the euclidean distances and medians of clusters can be determine by arguments like we see in `RSEC` and other functions.

```
ce<-assignUnassigned(ce,whichCluster="mergeClusters",reduceMethod="PCA",nDim=50,makePrimary=FALSE, filterIgnoresUnassigned=TRUE,clusterLabel="mergeClusters_AssignToCluster")
tableClusters(ce,whichCluster="mergeClusters_AssignToCluster")
```

```
## 
##  1  2  3  4 
## 15 15 15 20
```

Note that we chose `makePrimary=FALSE` (not the default) so that our original `mergeClusters` clustering remains the primary one, and doesn’t affect our future calls.

```
plotClusters(ce,whichCluster=c("mergeClusters_AssignToCluster","mergeClusters"))
```

You can also create a new object where all of the samples that are not assigned are removed with the `removeUnassigned` function. This is just a special case of `subsetByCluster` (above) for the special case of subsetting down to those samples assigned to any cluster.

## 4.3 Dimensionality reduction and SingleCellExperiment Class

There are two varieties of dimensionality reduction supported in `clusterExperiment` package.

1. reducing to a subset of features/genes based on the values of a filter statistic calculated for each gene or
2. creation of a smaller number of new features that are functions of the original features, i.e. *not* a simple selection of existing variables, but rather create new variables to represent the data

For simplicity, we’ll refer to the first as filtering of the data and second as a dimensionality reduction. This is because in the first case, the reduced data set can be quickly recreated by subseting the original data so long as the per-gene statistics have been saved. This means only a single vector of the length of the number of genes needs to be stored for the first type of dimensionality reduction (filtering) while the second kind requires saving a matrix with a value for each observation for each new variable.

`ClusterExperiment` inherits from the standard Bioconductor `SingleCellExperiment` class. Briefly, the `SingleCellExperiment` class extends the `SummarizedExperiment` class to give a structure for saving the reduced matrices from dimensionality reductions as we described above. They are saved in the slot `reducedDims`, which is a `SimpleList` of datasets that have the same number of observations as the original data in the assay slot, but reduced features (`?SingleCellExperiment`). This gives a unified way to save the results of applying a dimensionality reduction method of the second type; the package also gives helper functions to access them, etc. Multiple such dimensionality reductions can be stored since it is a list, and the user gives them names, e.g. “PCA” or “tSNE”.

`ClusterExperiment` uses the slot `reducedDims` to both save the results of dimensionality reductions if they are calculated and and also to reuse them if the necessary ones have already been created. This allows `clusterExperiment` to make use of any dimensionality reduction method so long as the user saves it in the appropriate slot in a `SingleCellExperiment` object. The user can also choose like before to have the function (like `clusterMany`) do the dimensionality reduction (i.e. PCA) internally. The difference is that now the results of the PCA will be stored in the appropriate slot so that they will not need to be recalculated in the future.

We also added in `clusterExperiment` package a similar procedure for storing the filtering statistics (i.e. statistics calculated on each gene). An example is the the variance across observations, calculated for every gene. `clusterExperiment` when calculating statistics (like `var` or `mad`) will add the per-gene value of the statistic as a column of the `rowData` of the `ClusterExperiment` object. Similarly, if the user has already calculated a per-gene statistic and saved it as a column in the `rowData` slot, this user-defined statistic can be used for filtering. This means that the user is not limited to the built-in filtering functions provided in `clusterExperiment`.

The functions `makeReducedDims` and `makeFilterStats` calculate the dimensionality reduction and filtering statistics, respectively, provided by `clusterExperiment` and store them in the appropriate slot. To see the current list of built-in functions:

```
listBuiltInReducedDims()
```

```
## [1] "PCA"
```

```
listBuiltInFilterStats()
```

```
## [1] "var"    "abscv"  "mad"    "mean"   "iqr"    "median"
```

# 5 Visualizing the data

In the quick start (above)[#visualsummary] we ran through many plots available for visualizing the data in conjunction with the clusterings. In this section we do not go through all of these plots, but just highlight the details of some plots which are more complicated.

## 5.1 Cluster Alignment plot with `plotClusters`

We demonstrated during the quick start that we can visualize the alignment of multiple clusterings via a Cluster Alignment plot implemented in `plotClusters` or `plotClustersWorkflow` command. Since `plotClustersWorkflow` calls the `plotClusters` command and passes the arguments of `plotClusters` onward, we will focus mainly on the main `plotClusters` command, with the understanding that most of these arguments work for both.

Here is our basic call to `plotClusters`:

```
par(mar=plotCMar)
plotClusters(ce,main="Clusters from clusterMany", whichClusters="workflow", 
             axisLine=-1)
```

We have seen that we can get very different plots depending on how we order the clusterings, and what clusterings are included. The argument `whichClusters` allows the user to choose different clusterings or provide an explicit ordering of the clusterings. For `plotClustersWorkflow`, `whichClusters` indicates the clusterings that are “highlighted” by being drawn separately from the results of `clusterMany`.

`whichClusters` can take either a single character value, or a vector of either characters or indices. If `whichClusters` matches either “all” or “workflow”, then the clusterings chosen are either all, or only those from the most recent calls to the workflow functions. Choosing “workflow” removes from the visualization both user-defined clusterings and also previous calls to the workflow that have since been rerun. Setting `whichClusters="workflow"` can be a useful if you have called a method like `makeConsensus` several times, as we did, only with different parameters. All of those runs are saved (unless `eraseOld=TRUE`), but you may not want to plot them.

If `whichClusters` is a character that is not one of these designated values, the entries should match a “clusterType” value (like “clusterMany”) or a “clusterLabel” value (with exact matching). Alternatively, the user can specify numeric indices corresponding to the columns of `clusterMatrix` that provide the order of the clusters.

```
par(mar=plotCMar)
plotClusters(ce,whichClusters="clusterMany",
               main="Only Clusters from clusterMany",axisLine=-1)
```

We can also add to our plot (categorical) information on each of our subjects from the `colData` of our SummarizedExperiment object (which is also retained in our ClusterExperiment object). This can be helpful to see if the clusters correspond to other features of the samples, such as sample batches. Here we add the values from the columns “Biological\_Condition” and “Published2” that were present in the `fluidigm` object and given with the published data.

```
par(mar=plotCMar)
plotClusters(ce,whichClusters="workflow", colData=c("Biological_Condition","Published2"), 
               main="Workflow clusters plus other data",axisLine=-1)
```

This options of plotting the data in the `colData`, however, is not currently available with `plotClustersWorkflow` which only plots clusterings.

### 5.1.1 Manipulations of colors

In this section we will talk about a number of related ways to manipulate the colors assigned to clusters in conjunction with the `plotClusters` command.

We will turn off the plotting of the cluster labels to make the plot less cluttered using the option `plot=FALSE`.

#### 5.1.1.1 Saving Assignment of colors from `plotClusters`

`plotClusters` invisibly returns a `ClusterExperiment` object. In our earlier calls to `plotCluster`, this would be the same as the input object and so there is no reason to save it. However, the alignment and color assignments created by `plotClusters` can be requested to be saved *into* the appropriate slots of the `ClusterExperiment` object in order to save the color and alignments of samples. This is done via the `resetNames`, `resetColors` and `resetOrderSamples` arguments. If any of these are set to TRUE, then the object returned will be different than those of the input.

Specifically, if `resetColors=TRUE` the `colorLegend` of the returned object will be changed so that the colors assigned to each cluster will be as were shown in the plot (and indeed this is done automatically by `mergeClusters` so that the `makeConsensus` and `mergeClusters` steps will have aligned color assignments). Similarly, if `resetNames=TRUE` the *names* of the clusters will be changed to be integer values, but now those integers will be aligned to try to be the same across clusters (and therefore will not be consecutive integers, which is why these are saved as *names* for the clusters and not the internal `clusterIds`). If `resetOrderSamples=TRUE`, then the order of the samples shown in the plot will be similarly saved in the slot `orderSamples`.

As an example, we will make a call to `plotClusters`, but now ask to reset everything to match this clusterAlignment.

First let’s look at what the object’s default colors are for the first two clusterings, accessed by `clusterLegend` function:

```
clusterLegend(ce)[c("mergeClusters","makeConsensus,final")]
```

```
## $mergeClusters
##      clusterIds color     name
## [1,] "-1"       "white"   "-1"
## [2,] "1"        "#1F78B4" "m1"
## [3,] "2"        "#33A02C" "m2"
## [4,] "3"        "#FF7F00" "m3"
## [5,] "4"        "#6A3D9A" "m4"
## 
## $`makeConsensus,final`
##      clusterIds color     name
## [1,] "-1"       "white"   "-1"
## [2,] "1"        "#1F78B4" "c1"
## [3,] "2"        "#33A02C" "c2"
## [4,] "3"        "#FF7F00" "c3"
## [5,] "4"        "#6A3D9A" "c4"
## [6,] "5"        "#B15928" "c5"
## [7,] "6"        "#A6CEE3" "c6"
## [8,] "7"        "#bd18ea" "c7"
```

The `plotClusterLegend` creates a quick legend plot of this information for a single clustering:

```
plotClusterLegend(ce,whichCluster="makeConsensus,final")
```

Now, we’ll run `plotClusters` and save the new colors. We’ll save this as a different object so that this is not a permanent change for the rest of the vignette, though in practice we would usually overwrite the existing `ce` to save memory on our computer and not have many versions floating around.

```
ce_temp<-plotClusters(ce,whichClusters="workflow", colData=c("Biological_Condition","Published2"), 
               main="Cluster Alignment of Workflow Clusters",clusterLabels=FALSE, axisLine=-1, resetNames=TRUE,resetColors=TRUE,resetOrderSamples=TRUE)
```

Now, the `clusterLegend` slot of the object no longer has the default color/name assignments, but it has names and colors that match across the clusters. Notice, that this means the prefix “m” or “c” that was previously given to distinguish the `makeConsensus` result from the `mergeClusters` result is now gone (the user could manually add them by changing the values in the clusterLegend). Instead, there are names that “match up” the clusters across the different clusterings.

```
clusterLegend(ce_temp)[c("mergeClusters","makeConsensus,final")]
```

```
## $mergeClusters
##      clusterIds color     name
## [1,] "-1"       "white"   "-1"
## [2,] "1"        "#E31A1C" "1" 
## [3,] "2"        "#1F78B4" "2" 
## [4,] "3"        "#33A02C" "3" 
## [5,] "4"        "#FF7F00" "4" 
## 
## $`makeConsensus,final`
##      clusterIds color     name
## [1,] "-1"       "white"   "-1"
## [2,] "1"        "#E31A1C" "1" 
## [3,] "2"        "#1F78B4" "2" 
## [4,] "3"        "#33A02C" "3" 
## [5,] "4"        "#FF7F00" "4" 
## [6,] "5"        "#B15928" "6" 
## [7,] "6"        "#A6CEE3" "7" 
## [8,] "7"        "#bd18ea" "8"
```

#### 5.1.1.2 Manual Assignment of colors

A similar setting can be that we have colors we want to manually set for a particular cluster, but we want to have the other clusterings get aligned to the colors of that clustering. We can use `plotClusters` to assign colors to the other clusterings so that the other clusterings inherit the colors of the cluster of interest.

Let’s manually set the colors for the “mergeClusters” clustering. We’ll again create a (new) `ce_temp` object so again we don’t overwrite the previous colors for the rest of the vignette. Again, the color information is accessed with the `clusterLegend` command:

```
ce_temp<-ce
clusterLegend(ce_temp)[["mergeClusters"]]
```

```
##      clusterIds color     name
## [1,] "-1"       "white"   "-1"
## [2,] "1"        "#1F78B4" "m1"
## [3,] "2"        "#33A02C" "m2"
## [4,] "3"        "#FF7F00" "m3"
## [5,] "4"        "#6A3D9A" "m4"
```

We will just assign new colors to the `color` column with the `recolorClusters` function. We can also give them new names too with `renameClusters`.

```
newColors<-c("white","blue","green","cyan","purple")
newNames<-c("Not assigned","Cluster1","Cluster2","Cluster3","Cluster4")
#note we make sure they are assigned to the right cluster Ids by giving the 
#clusterIds as names to the new vectors
names(newColors)<-names(newNames)<-clusterLegend(ce_temp)[["mergeClusters"]][,"name"]
renameClusters(ce_temp,whichCluster="mergeClusters",value=newNames)
```

```
## class: ClusterExperiment 
## dim: 7069 65 
## reducedDimNames: PCA 
## filterStats: var var_makeConsensus.final 
## -----------
## Primary cluster type: mergeClusters 
## Primary cluster label: mergeClusters 
## Table of clusters (of primary clustering):
##     Cluster1     Cluster2     Cluster3     Cluster4 Not assigned 
##           15           14           13           17            6 
## Total number of clusterings: 41 
## Dendrogram run on 'makeConsensus,final' (cluster index: 2)
## -----------
## Workflow progress:
## clusterMany run? Yes 
## makeConsensus run? Yes 
## makeDendrogram run? Yes 
## mergeClusters run? Yes
```

```
recolorClusters(ce_temp,whichCluster="mergeClusters",value=newColors)
```

```
## class: ClusterExperiment 
## dim: 7069 65 
## reducedDimNames: PCA 
## filterStats: var var_makeConsensus.final 
## -----------
## Primary cluster type: mergeClusters 
## Primary cluster label: mergeClusters 
## Table of clusters (of primary clustering):
## -1 m1 m2 m3 m4 
##  6 15 14 13 17 
## Total number of clusterings: 41 
## Dendrogram run on 'makeConsensus,final' (cluster index: 2)
## -----------
## Workflow progress:
## clusterMany run? Yes 
## makeConsensus run? Yes 
## makeDendrogram run? Yes 
## mergeClusters run? Yes
```

We use the `plotClusterLegend` to check that we assigned them as we expected:

```
plotClusterLegend(ce_temp,whichCluster="mergeClusters")
```

Now we will run the `plotClusters` alignment plot, but we will direct the alignment to use the cluster colors we just gave for the “mergeClusters” cluster. We do this by using the argument `existingColors="firstOnly"` – but as the argument option indicates it only works if the “mergeClusters” clustering is the first clustering in the plot.

```
plotClusters(ce_temp,whichClusters="workflow", colData=c("Biological_Condition","Published2"), clusterLabels=FALSE,
               main="Clusters from clusterMany, different order",axisLine=-1,existingColors="firstOnly")
```

This just created the visualization. We can also save the results of this as we did before with `resetColors=TRUE`, so that now all of our future clusters make use of this color information. We won’t reset the names, however. Note we can avoid making the plot again with the argument `plot=FALSE`.

```
ce_temp<-plotClusters(ce_temp,whichClusters="workflow", colData=c("Biological_Condition","Published2"), resetColors=TRUE,
               main="Clusters from clusterMany, different order",axisLine=-1, clusterLabels=FALSE, existingColors="firstOnly",plot=FALSE)
```

#### 5.1.1.3 Using only the assigned colors

Once we start manually changing the colors, we will sometimes want to align our clusters, but *use the existing cluster colors* that are saved in the object. We can force `plotClusters` to use all the existing color assignments, rather than create its own, with the argument `existingColors="all"`. This makes particular sense if you want to have continuity between plots – i.e. be sure that a particular cluster always has a certain color – but would like to do different variations of plotClusters to get a sense of how similar the clusters are.

For example, we set the colors above based on the cluster alignment produced in the above `plotClusters` where the clusterings were ordered according to the workflow and making use of the colors we manually assigned to “mergeClusters”. But now we want to plot only the clusters from `clusterMany`, yet keep the same colors that we just saved so we can compare them. We do this by setting the argument `existingColors="all"`, meaning use all of the existing colors.

```
par(mfrow=c(1,2))
plotClusters(ce_temp, colData=c("Biological_Condition","Published2"),
             whichClusters="workflow", existingColors="all", clusterLabels=FALSE,
             main="All Workflow Clusters, use existing colors",axisLine=-1)

plotClusters(ce_temp, colData=c("Biological_Condition","Published2"), 
             existingColors="all", whichClusters="clusterMany", clusterLabels=FALSE,
             main="clusterMany Clusters, use existing colors",
             axisLine=-1)
```

We see that while the order of the samples has changed (because I have a different set of clusters to align) the *colors* assigned to each cluster have stayed the same, so I can more easily compare the plots.

Note that the use of `existingColors="firstOnly"` and `existingColors="all"` will not give the same color assignments, *even if the colors have been previously aligned to be the same* unless its the exact same set of clusterings in the same order. This is because with each set of ordered clusterings, the realignment between clusters changes and so the assignment of colors changes. Here we will make a ClusterAlignment plot for each of these three options of the argument to demonstrate the difference.

```
par(mfrow=c(2,2))
plotClusters(ce_temp, colData=c("Biological_Condition","Published2"), 
             existingColors="all", whichClusters="clusterMany", clusterLabels=FALSE,
             main="clusterMany Clusters, use existing colors",
             axisLine=-1)
plotClusters(ce_temp, colData=c("Biological_Condition","Published2"), 
               existingColors="firstOnly", whichClusters="clusterMany", clusterLabels=FALSE,
               main="clusterMany Clusters, use existing of first row only",
               axisLine=-1)
plotClusters(ce_temp, colData=c("Biological_Condition","Published2"), 
            existingColors="ignore", whichClusters="clusterMany", clusterLabels=FALSE,
            main="clusterMany Clusters, default\n(ignoring assigned colors)",
            axisLine=-1)
```

#### 5.1.1.4 Choosing a different set of colors

`plotClusters` uses the set of colors defined in the variable `massivePalette`. This is a set of 484 colors. `plotClusters` draws from this set of colors sequentially as it goes down the clusterings each time it needs a new color. This is obviously a very large list of colors; the reason for such a long list is that `plotClusters` will error out if there are not enough colors, so we want to have a large list. If we are running RSEC with many clusterings, and each clustering has many clusters, you can quickly run through many. The first 56 colors are a smaller subset of colors saved as a variable `bigPalette`, and these have been hand-chosen so that the colors are vibrant and not too similar to each other; the order has also been chosen so that more similar colors are not next to each other. This are the colors that are most frequently seen. `massivePalette` consists of these colors, plus all of the non-grey colors in `colors()` that are not already contained in `bigPalette`

We can examine the colors in `bigPalette` with the command `showPalette`:

```
showPalette()
```

This plot shows us both the name of the color (on the top) and the index of the color in `bigPalette`.

We can show a smaller subset or give an arbitrary set of colors to show with `showPalette`

```
showPalette(which=1:10)
```

```
showPalette(palette())
```

We can even show the entire `massivePalette` (notice for list of colors > 100 in length, the index is no longer plotted)

```
showPalette(massivePalette,cex=0.5)
```

We can use this information to help change our color choices. For example, suppose I want the unassigned samples to be given the color black instead of white. Then I would like to not use the black in `bigPalette` to assign to a clustering. I will use the `colPalette` argument to give a new set of colors that does not include “black”. And I will set the unassigned samples using the option `unassignedColor="black"` (there is a similar argument `missingColor` to set the color assigned to those clusters with the identification of “-2”, meaning they were not included in the clustering at all).

```
plotClusters(ce,whichClusters="workflow", colData=c("Biological_Condition","Published2"), unassignedColor="black",colPalette=bigPalette[-grep("black",bigPalette)],
               main="Setting unassigned color",axisLine=-1)
```

### 5.1.2 Variant version: `plotClustersWorkflow`

`plotClusters` is a generic function for showing any clusterings. In following our workflow, however, there can often be many clusterings from `clusterMany` and the important clusters from `makeConsensus` and `mergeMany` can get lost from view. The function `plotClustersWorkflow` implements `plotClusters` but pulls out specific clusterings designated by the user and puts them apart from the results of `clusterMany`.

```
plotClustersWorkflow(ce, axisLine= -1)
```

By default, the “makeConsensus” and “mergeClusters” clusterings are highlighted. We can choose other clusterings to be the result, rather than the default ones using the argument `whichClusters`. We can also sort the clusterings differently so that we sort based on the results of `clusterMany` first, rather than the highlighted results first and/or choose to have the highlighted clusters on the bottom or the top (independently of the sorting choices). In the following code, we choose to show the different versions of `makeConsensus` as our highlighted clusters.

```
par(mar=plotCMar)
plotClustersWorkflow(ce,whichClusters=c("makeConsensus,final","makeConsensus,0.7","makeConsensus,1"),main="Different choices of proportion in makeConsensus",sortBy="clusterMany",highlightOnTop=FALSE, axisLine= -1)
```

There are no limits as to which clusterings can be shown with the `whichClusters` command, but the non-highlighted clusterings must always be of `clusterType` “clusterMany”. The user can select a subset or different ordering of the “clusterMany” clusterings with the argument `whichClusterMany`, which must be a numeric vector giving the indices of the clusters to be chosen.

## 5.2 Heatmap including the clusters with `plotHeatmap`

There is also a default heatmap command for a `ClusterExperiment` object that we used in the Quick Start. By default it clusters on the most variable features (after transforming the data) and shows the `primaryCluster` alongside the data. The `primaryCluster`, now that we’ve run the workflow, has been set as that from the last mergeClusters step.

```
par(mfrow=c(1,1))
par(mar=defaultMar)
plotHeatmap(ce,main="Heatmap with clusterMany")
```

The `plotHeatmap` command has numerous options, in addition to those of `aheatmap`. `plotHeatmap` mainly provides additional functionality in the following areas:

- Easy inclusion of clustering information or sample information, based on the ClusterExperiment object.
- Additional methods for ordering/clustering the samples that makes use of the clustering information.
- Use of separate input data for clustering and for visualization.
- Setting the breaks for better visualization

### 5.2.1 Displaying clustering or sample information

Like `plotClusters`, `plotHeatmap` has a `whichClusters` option that behaves similarly to that of `plotClusters`. In addition to the options “all” and “workflow” that we saw with `plotClusters`, `plotHeatmap` also takes the option “none”" (no clusters shown) and “primary” (only the primaryCluster). The user can also request a subset of the clusters by giving specific indices to `whichClusters` like in `plotClusters`.

Here we create a heatmap that shows the clusters from the workflow. Notice that we choose only the last 2 – from `makeConsensus` and `mergeClusters`. If we chose all “workflow” clusters it would be too many.

```
whClusterPlot<-1:2
plotHeatmap(ce,whichClusters=whClusterPlot, annLegend=FALSE)
```

Notice we also passed the option ‘annLegend=FALSE’ to the underlying `aheatmap` command (with many clusterings shown, it is often not useful to have a legend for all the clusters because the legend doesn’t fit on the page!). The many detailed commands of `aheatmap` that are not set internally by `plotHeatmap` can be passed along as well.

Like `plotClusters`, `plotHeatmap` takes an argument `colData`, which refers to columns of the `colData` of that object and can be included.

### 5.2.2 Additional options for clustering/ordering samples

We can choose to not cluster the samples, but order the samples by cluster. This time we’ll just show the primary cluster (the `mergeCluster` result) by setting `whichClusters="primaryCluster"`:

```
plotHeatmap(ce,clusterSamplesData="primaryCluster",
            whichClusters="primaryCluster",
            main="Heatmap with clusterMany",annLegend=FALSE)
```

As an improvement upon this, we can cluster the clusters into a dendrogram so that the most similar clusters will be near each other. We already did this before with our call to `makeDendrogram`. We haven’t done anything to change that, so the dendrogram from that call is still stored in the object. We can check this in the information shown in our object:

```
show(ce)
```

```
## class: ClusterExperiment 
## dim: 7069 65 
## reducedDimNames: PCA 
## filterStats: var var_makeConsensus.final 
## -----------
## Primary cluster type: mergeClusters 
## Primary cluster label: mergeClusters 
## Table of clusters (of primary clustering):
## -1 m1 m2 m3 m4 
##  6 15 14 13 17 
## Total number of clusterings: 41 
## Dendrogram run on 'makeConsensus,final' (cluster index: 2)
## -----------
## Workflow progress:
## clusterMany run? Yes 
## makeConsensus run? Yes 
## makeDendrogram run? Yes 
## mergeClusters run? Yes
```

We can see, as we expected, that the dendrogram we made from “makeConsensus,final” is still the active dendrogram saved in the `ce` object. Now we will call `plotHeatmap` choosing to display the “mergeClusters” and “makeConsensus,final” clustering, and ordering the samples by the dendrogram in `ce`. We will also display some data about the samples from the `colData` slot using the `colData` argument. Notice that instead of giving the index, we can also give the clusterLabels of the clusters we want to show.

```
plotHeatmap(ce,clusterSamplesData="dendrogramValue",
            whichClusters=c("mergeClusters","makeConsensus"),
            main="Heatmap with clusterMany",
            colData=c("Biological_Condition","Published2"),annLegend=FALSE)
```

If there is not a dendrogram stored, `plotHeatmap` will call `makeDendrogram` based on the primary cluster (with the default settings of `makeDendrogram`); calling `makeDendrogram` on `ce` is preferred so that the user can control the choices in how it is done (which we will discuss below). For visualization purposes, the dendrogram for the `makeConsensus` cluster is preferred to that of the `mergeCluster` cluster, since “makeConsensus,final” is just a finer partition of the “mergeClusters” clustering.

### 5.2.3 Using separate input data for clustering and for visualization

While count data is a common type of data, it is also common that the input data in the SummarizedExperiment object might be normalized data from a normalization package such as `RUVSeq`. In this case, the clustering and all numerical calculations should be done on the normalized data (which may or may not need a log transform). However, these normalized data might not be on a logical count scale (for example, in `RUVSeq`, the normalize data are residuals after subtracting out gene-specific batch effects).

In this case, it can be convenient to have the *visualization* of the data (i.e. the color scale), be based on a count scale that is interpretable, even while the clustering is done based on the normalized data. This is possible by giving a new matrix of values to the argument `visualizeData`. In this case, the color scale (and clustering of the features) is based on the input `visualizeData` matrix, but all clustering of the samples is done on the internal data in the `ClusterExperiment` object.

### 5.2.4 Setting the breaks

Usually, the breaks that determine the colors of the heatmap are evenly spaced across the range of the data in the entire matrix. When there are a few outlier samples or genes, they can dominate the color and make it impossible to visualize the bulk of the data.

For this reason, the argument `breaks` in `plotHeatmap` allows for a value between 0 and 1, to indicate that the range of colors should be chosen as equally spaced between certain quantiles of the data. For example, if `breaks=0.99`, the range of equally spaced breaks will stop at the top 0.99 quantile of the data and anything above that value gets assigned the single extreme color. If there is negative data in the matrix, then it also will use the lower quantile of the data to stop the range of equally spaced breaks (see `?setBreaks`)

Here

```
plotHeatmap(ce,clusterSamplesData="primaryCluster",
            whichClusters="primaryCluster", breaks=0.99,
            main="Heatmap with clusterMany, breaks=0.99",annLegend=FALSE)
```

```
plotHeatmap(ce,clusterSamplesData="primaryCluster",
            whichClusters="primaryCluster", breaks=0.95,
            main="Heatmap with clusterMany, breaks=0.95",annLegend=FALSE)
```

The function `setBreaks` which is called internally by `plotHeatmap` is also a stand-alone function that the user can call directly to have greater flexibility in getting breaks for the heatmap. For example it allows the user to specify that the breaks should be symmetric around 0. We also provide some default color spectrum that can be better for different settings or symmetric data around 0 – see `?showHeatmapPalettes`

## 5.3 Dendrogram of clusters with `plotDendrogram`

We saw above that we can quickly see the hierarchies of the clusters using the `plotDendrogram` function.

```
plotDendrogram(ce)
```

This shows us the dendrogram stored in the object (created from the clustering resulting from the `makeConsensus` step). The relative sizes of each cluster are shown, along with a legend of the clusters.

### 5.3.1 Changing the leaf and plot type

Alternatively, we can just make a simpler plot with `plotDendrogram` by use of the `leafType` and `plotType` arguments. The above plot uses the defaults: `leafType="samples"`, meaning plot the individual samples at the leaves of the tree, and `plotType="colorblock"` meaning to display the leaves’ values as blocks of colors. Here we change these options

```
par(mfrow=c(1,2))
plotDendrogram(ce,leafType="clusters",plotType="colorblock")
plotDendrogram(ce,leafType="clusters",plotType="name")
```

If we have a small number of samples, we can even choose `leafType="samples"` and `plotType="name"` to show the names of the individual samples, but this is usually not the case.

### 5.3.2 Changing the information plotted with dendrogram

#### 5.3.2.1 Additional clusters

If the option is `plotType="colorblock"` and `leafType="samples"` (i.e. the default) we can also vary the sample information that is plotted with the dendrogram, much like with `plotHeatmap`. In particular, we can show multiple clusters using the option `whichClusters`.

```
par(mar=plotDMar)
plotDendrogram(ce,whichClusters=c("makeConsensus","mergeClusters"))
```

#### 5.3.2.2 Merge Information

We can also plot the merging information, with `merge="mergeMethod"`, i.e. the method actually used to merge the data. We could alternatively plot any merge method that has been run from a call to `mergeClusters`.

```
par(mar=plotDMar)
plotDendrogram(ce,whichClusters=c("makeConsensus","mergeClusters"),merge="mergeMethod")
```

#### 5.3.2.3 Sample data

We can also include data stored in `colData` slot of the object, so long as it is discrete (i.e. a factor or character vector).

```
plotDendrogram(ce,whichClusters=c("makeConsensus","mergeClusters"),colData=c("Biological_Condition","Published1","Published2"),merge="mergeMethod")
```

#### 5.3.2.4 Node information

We can pass options to `plot.phylo` to change the output of the dendrogram. For example, we can print the node names or colors For example, to see the node names, we can set `show.node.label = TRUE` (we have to turn off plotting the merge information, since the merge information is plotted as node labels too.)

```
par(mar=plotDMar)
plotDendrogram(ce,whichClusters=c("makeConsensus","mergeClusters"),show.node.label=TRUE)
```

We can also make the nodes colors (useful for linking up with colors from `plotContrastHeatmap` if using hierarchical contrasts, see below). Here we give own argument `nodeColors` (not that from `plot.phylo`) to make it easier; the argument takes a vector of colors, where the names of the vectors match the node names. Notice, we can plot the merge information with node colors.

```
nodeNames<-paste("Node",1:6,sep="")
nodeColors<-massivePalette[1:length(nodeNames)]
names(nodeColors)<-nodeNames
par(mar=plotDMar)
plotDendrogram(ce,whichClusters=c("makeConsensus","mergeClusters"),merge="mergeMethod",nodeColors=nodeColors)
```

# 6 The clustering workflow

We will now go into more detail about important options for the main parts of the clustering workflow.

## 6.1 clusterMany

In the quick start section we picked some simple and familiar clustering options that would run quickly and needed little explanation. However, our workflow generally assumes more complex options and more parameter variations are tried. Before getting into the specific options of `clusterMany`, let us first describe some of these more complicated setups, since many of the arguments of `clusterMany` depend on understanding them.

### 6.1.1 Base clustering algorithms and the ClusterFunction class

This package is meant to be able to use and compare different clustering routines. However, the required input, arguments, etc. of different clustering algorithms varies greatly. We create the `ClusterFunction` class so that we can ensure that the necessary information to fit into our workflow is well defined, and otherwise the other details of the algorithm can be ignored by the workflow. In general, the user will not need to know the details of this class if they want to use the built in functions provided by the package, which can be accessed by character values. To see the set of character values that correspond to built-in functions,

```
listBuiltInFunctions()
```

```
## [1] "pam"            "clara"          "kmeans"         "hierarchical01"
## [5] "hierarchicalK"  "tight"          "spectral"
```

If you are interested in implementing your own `ClusterFunction` object, after reading this section look at our (example)[#customAlgorithm] below.

There are some important features of any clustering algorithm that are encoded in the `ClusterFunction` object for which it is important to understand because they affect which algorithms can be used when at different parts of the workflow.

#### 6.1.1.1 `inputType`

The type of input the algorithm expects, which can be either an \(p x n\) matrix of features, in which case the argument `x` gives that data, or a \(n x n\) matrix of dissimilarities, in which case the argument `diss`. Some algorithms can accept either type. To determine the `inputType` of an algorithm(s),

```
inputType(c("kmeans","pam","hierarchicalK"))
```

```
##        kmeans           pam hierarchicalK 
##           "X"      "either"        "diss"
```

#### 6.1.1.2 `algorithmType`

We group together algorithms that cluster based on common strategies that affect how we can use them in our workflow. Currently there are two “types” of algorithms we consider, which we call type “K” and “01”. We can determine the type of a builtin function by the following:

```
algorithmType(c("kmeans","hierarchicalK","hierarchical01"))
```

```
##         kmeans  hierarchicalK hierarchical01 
##            "K"            "K"           "01"
```

The “K” algorithms are so called because their main parameter requirement is that the user specifies the number of clusters (\(K\)) to be created and require an input of `k` to the clustering function. Built in ‘K’ algorithms are:

```
listBuiltInTypeK()
```

```
## [1] "pam"           "clara"         "kmeans"        "hierarchicalK"
## [5] "spectral"
```

The “01” algorithms are so named because the algorithm assumes that the input is a *disimilarities* between samples and that the similarities encoded in \(D\) are on a scale of 0-1. The clustering functions should use this fact to make the primary user-specified parameter be not the number of final clusters, but a measure \(\alpha\) of how dissimilar samples in the same cluster can be (on a scale of 0-1). Given \(\alpha\), the algorithm then implements a method to then determine the clusters (so \(\alpha\) implicitly determines \(K\)). These methods rely on the assumption that because the 0-1 scale has special significance, the user will be able to make an determination more easily as to the level of dissimilarity allowed in a true cluster, rather than predetermine the number of clusters \(K\). The current 01 methods are:

```
listBuiltInType01()
```

```
## [1] "hierarchical01" "tight"
```

#### 6.1.1.3 `requiredArgs`

The different algorithm types correspond to requiring different input types (`k` versus `alpha`). This is usually sorted out by `clusterMany`, which will only dispatch the appropriate one. Clustering functions can also have additional required arguments. See below for more discussion about how these arguments can be passed along to `clusterMany` or `RSEC`.

To see all of the required arguments of a function,

```
requiredArgs(c("hierarchical01","hierarchicalK"))
```

```
## $hierarchical01
## [1] "alpha"
## 
## $hierarchicalK
## [1] "k"
```

### 6.1.2 Internal clustering procedures

`clusterMany` iteratively calls a function `clusterSingle` over the collection of parameters. `clusterSingle` is the clustering workhorse, and may be used by the user who wants more fine-grained control, see documentation of `clusterSingle`.

Within each call of `clusterSingle`, there are three possible steps, depending on the value of the arguments `subsample` and `sequential`:

1. Subsampling (`subsampleClustering`) – if `subsample=TRUE`
2. Main Clustering (`mainClustering`)
3. Sequential (`seqCluster`) – if `sequntial=TRUE`

If both `sequential` and `subsample` are `FALSE`, then step 1 and step 3 are skipped and `clusterSingle` just calls the `mainClustering` (step 2), resulting in a basic clustering routine applied to the input data. If `subsample=TRUE`, then step 1 (`subsampleClustering`) is called which subsamples the input data and clusters each subsample to calculate a co-occurance matrix. That co-occurance matrix is used as the input for `mainClustering` (step 2). If `sequential=TRUE` this process (step 1 then step 2 if `subsample=TRUE` or just step 2 if `subsample=FALSE`) is iterated over and over again to iteratively select the best clusters (see `?seqCluster` for a detailed description). Each of these steps has a function that goes with it, noted above, but they should not generally be called directly by the user. However, the documentation of these functions can be useful.

In particular, arguments to these three functions that are not set by `clusterMany` can be passed via *named* lists to the arguments: `subsampleArgs`, `mainClusterArgs`, and `seqArgs`. Some of the arguments to these functions can be varied in `clusterMany`, but more esoteric ones should be sent as part of the named list of parameters given to `clusterMany`; those named lists will be fixed for all parameter combinations tried in `clusterMany`.

#### 6.1.2.1 Main Clustering (Step 2): `mainClustering`

The main clustering (step 2) described above is done by the function `mainClustering`. In addition to the basic clustering algorithms on the input data, we also implement many other common cluster processing steps that are relevant to the result of the clustering. We have already seen such an example with dimensionality reduction, where the input \(D\) is determined based on different input data. Many of the arguments to `mainClustering` are arguments to `clusterMany` as well so that `mainClusterArgs` is usually not needed. The main exception would be to send more esoteric arguments to the underlying clustering function called in the main clustering step. The syntax for this would be to give a nested list to the argument `mainClusterArgs`

```
clusterMany(x,clusterFunction="hierarchicalK", ... , mainClusterArgs=list(clusterArgs=list(method="single") ))
```

Here we change the argument `method` in the clustering function `hclust` called by the `hierarchicalK` function to `single`.

#### 6.1.2.2 Subsampling (step 1) `subsampleClustering`

A more significant process that can be coupled with any clustering algorithm is to continually by subsample the data and cluster the subsampled data. This creates a \(n x n\) matrix \(S\) that is a matrix of co-clustering percentages – how many times two samples co-clustered together over the subsamples (there are slight variations as how this can be calculated, see help pages of `subsampleClustering` ). This does not itself give a clustering, but the resulting \(S\) matrix can then form the basis for clustering the samples. Specifically, the matrix \(D=1-S\) is then given as input to the main clustering step described above. The subsampling option is computationally expensive, and when coupled with comparing many parameters, does result in a lengthy evaluation of `clusterMany`. However, we recommend it if the number of samples is not too large as one of the most useful methods for getting stable clustering results.

Note that the juxtaposition of step 1 then step 2 (the subsampling and then feeding the results to the main clustering function) implies there actually two different possible clustering algorithms (and sets of corresponding parameters) – one for the clustering on the subsampled data, and one for the clustering of the resulting \(D\) based on the percentage of coClustering of samples. This brings up a restriction on the clustering function in the main clustering step – it needs to be able to handle input that is a dissimilarity (`inputType` is either `diss` or `either`).

Furthermore, the user might want to set clustering function and corresponding parameters separately for step 1 and step 2. The way that `clusterMany` handles this is that the main arguments of `clusterMany` focus on varying the parameters related to step 2 (the main clustering step, i.e. the clustering of \(D\) after subsampling). For this reason, the argument `clusterFunction` in `clusterMany` varies the clustering function used by the main clustering (step 2), not the subsampling step. The clustering function of the subsampling (step 1) can be specified by the user via `subsampleArgs`, but in this case it is set for *all* calls of `clusterMany` and does not vary. Alternatively, if the user doesn’t specify the `clusterFunction` in `subsampleArgs` then the default is to use `clusterFunction` of the main clustering step along with any required arguments given by the user for that function (there are some cases where using the `clusterFunction` of the main step is not possible for the subsampling step, in which case the default is to use “pam”).

More generally, since few of the arguments to `subsampleClustering` are allowed to be varied by the direct arguments to `clusterMany`, it is also more common to want to change these arguments via the argument `subsampleArgs`. Examples might be `resamp.num` (the number of subsamples to draw) or `samp.p` (the proportion of samples to draw in each subsample) – see `?subsampleClustering` for a full documentation of the possible arguments. In addition, there are arguments to be passed to the underlying clustering function; like for `mainClustering`, these arguments would be a nested list to the argument `subsampleArgs`.

An example of a syntax that sets the arguments for `subsampleClustering` would be:

```
clusterMany(x,..., subsampleArgs=list(resamp.num=100,samp.p=0.5,clusterFunction="hiearchicalK", clusterArgs=list(method="single") ))
```

#### 6.1.2.3 Sequential Detection of Clusters (Step 3): `seqCluster`

Another complicated addition that can be added to the main clustering step is the implementation of sequential clustering. This refers to clustering of the data, then removing the “best” cluster, and then re-clustering the remaining samples, and then continuing this iteration until all samples are clustered (or the algorithm in some other way calls a stop). Such sequential clustering can often be convenient when there is very dominant cluster, for example, that is far away from the other mass of data. Removing samples in these clusters and resampling can sometimes be more productive and result in a clustering more robust to the choice of samples. A particular implementation of such a sequential method, based upon (Tseng and Wong 2005), is implemented in the `clusterExperiment` package when the option `sequential=TRUE` is chosen (see `?seqCluster` for documentation of how the iteration is done). Sequential clustering can also be quite computationally expensive, particularly when paired with subsampling to determine \(D\) at each step of the iteration.

Because of the iterative nature of the sequential step, there are many possible parameters (see `?seqCluster`). Like subsample clustering, `clusterMany` does not allow variation of very many of these parameters, but they can be set via passing arguments in a named list to `seqArgs`. An example of a syntax that sets the arguments for `seqCluster` would be:

```
clusterMany(x,..., seqArgs=list( remain.n=10))
```

This code changes the `remain.n` option of the sequential step, which governs when the sequential step stops because there are not enough samples remaining.

### 6.1.3 Arguments of `clusterMany`

Now that we’ve explained the underlying architecture of the clustering provided in the package, and how to set the arguments that can’t be varied, we discuss the parameters that *can* be varied in `clusterMany`. (There are a few additional arguments available for `clusterMany` that govern how `clusterMany` works, but right now we focus on only the ones that can be given multiple options).

Recall that arguments in `clusterMany` that take on multiple values mean that the combinations of all the multiple valued arguments will be given as input for a clustering routine. These arguments are:

- `sequential` This parameter consists of logical values, TRUE and/or FALSE, indicating whether the sequential strategy should be implemented or not.
- `subsample` This parameter consists of logical values, TRUE and/or FALSE, indicating whether the subsampling strategy for determining \(D\) should be implemented or not.
- `clusterFunction` The clustering functions to be tried in the *main clustering step*. Recall if `subsample=TRUE` is part of the combination, then `clusterFunction` the method that will be used on the matrix \(D\) created from subsampling the data. Otherwise, `clusterFunction` is the clustering method that will be used directly on the data.
- `ks` The argument ‘ks’ is interpreted differently for different choices of the other parameters *and can differ from between parameter combinations!*. If `sequential=TRUE` is part of the parameter combination, `ks` defines the argument `k0` of sequential clustering (see `?seqCluster`), which is approximately like the initial starting point for the number of clusters in the sequential process. Otherwise, `ks` is passed to set `k` of both the main clustering step (and by default that of the subsampled data), and is only relevant if `clusterFunction` is of type “K”. When/if `findBestK=TRUE` is part of the combination, `ks` also defines the range of values to search for the best k (see the details in the documentation of `clusterMany` for more).
- `reduceMethod` These are character strings indicating what choices of dimensionality reduction should be tried. These can indicate any combination of either filtering statistics or dimensionality reductions. The character strings can either refer to built-in methods, meaning `clusterMany` will do the necessary calculations and save the results as an initial step, OR the vector can refer to filtering statistics/dimensionality reductions that have already been calculated and saved in the object (see (above)[#dimReduce] for more information). The vector cannot be a combination of these two. If either a dimensionality reduction or a filtering statistic are chosen, the following parameters can also be varied to indicate the number of such features to be used (with a vector of values meaning all will be tried):
  - `nFilterDims`
  - `nReducedDims`
- `distFunction` These are character values giving functions that provide a distance matrix between the samples, when applied to the data. These functions should be accessible in the global environment (`clusterMany` applies `get` to the global environment to access these functions). To make them compatible with the standard R function `dist`, these functions should assume the samples are in the rows, i.e. they should work when applied to t(assay(ce)). We give an example in the next subsection below.
- `minSizes` these are integer values determining the minimum size required for a cluster (passed to the `mainClustering` part of clustering).
- `alphas` These are the \(\alpha\) parameters for “01” clustering techniques; these values are only relevant if one of the `clusterFunction` values is a “01” clustering algorithm. The values given to `alphas` should be between 0 and 1, with smaller values indicating greater similarity required between the clusters.
- `betas` These are the \(\beta\) parameters for sequential clustering; these values are only relevant if `sequential=TRUE` and determine the level of stability required between changes in the parameters to determine that a cluster is stable.
- `findBestK` This option is for “K” clustering techniques, and indicates that \(K\) should be chosen automatically as the \(K\) that gives the largest silhouette distance between clusters.
- `removeSil` A logical value as to whether samples with small silhouette distance to their assigned cluster are “removed”, in the sense that they are not given their original cluster assignment but instead assigned -1. This option is for “K” clustering techniques as a method of removing poorly clustered samples.
- `silCutoff` If `removeSil` is TRUE, then `silCutoff` determines the cutoff on silhouette distance for unassigning the sample.

`clusterMany` tries to have generally simple interface, and for this reason makes choices about what is meant by certain combinations of parameters. For example, in combinations where `findBestK=TRUE`, `ks=2:10` is taken to mean that the clustering should find the best \(k\) out of the range of 2-10. However, in other parameter combinations where `findBestK=FALSE` the same `ks` might indicate the specific number of clusters, \(K\), that should be found. To see the parameter choices that will be run, the user can set `run=FALSE` and the output will be a matrix of the parameter values indicated by the choices of the user. For parameter combinations that are not what is desired, the user should consider making direct calls to `clusterSingle` where all of these options combinations (and many more) can be explicitly called.

Other parameters for the clustering are kept fixed. As described above, there are many more possible parameters in play than are considered in `clusterMany`. These parameters can be set via the arguments `mainClusterArgs`, `subsampleArgs` and `seqArgs`. These arguments correspond to the different processes described above (the main clustering step, the creation of \(D\) to be clustered via subsampling, and the sequential clustering process, respectively). These arguments take a list of arguments that are sent directly to `clusterSingle`. However, these arguments may be overridden by the interpretation of `clusterMany` of how different combinations interact; again for complete control direct calls to `clusterSingle` are necessary.

| Argument | Dependencies | Passed to | Argument passed to |
| --- | --- | --- | --- |
| ks | sequential=TRUE | seqCluster | k0 |
| - | sequential=FALSE, findBestK=FALSE, clusterFunction of type ‘K’ | mainClustering | k |
| - | sequential=FALSE, findBestK=FALSE, subsample=TRUE | subsampleClustering | k |
| - | sequential=FALSE, findBestK=TRUE, clusterFunction of type ‘K’ | mainClustering | kRange |
| reduceMethod | none | transform | reduceMethod |
| nFilterDims | reduceMethod in ‘mad’,‘cv’,‘var’ | transform | nFilterDims |
| nReducedDims | reduceMethod=‘PCA’ | transform | nReducedDims |
| clusterFunction | none | mainClustering | clusterFunction |
| minSizes | none | mainClustering | minSize |
| distFunction | subsample=FALSE | mainClustering | distFunction |
| alphas | clusterFunction of type ‘01’ | mainClustering | alpha |
| findBestK | clusterFunction of type ‘K’ | mainClustering | findBestK |
| removeSil | clusterFunction of type ‘K’ | mainClustering | removeSil |
| silCutoff | clusterFunction of type ‘K’ | mainClustering | silCutoff |
| betas | sequential=TRUE | seqCluster | beta |

### 6.1.4 Example changing the distance function

Providing different distance functions is slightly more involved than the other parameters, so we give an example here.

First we define distances that we would like to compare. We are going to define two distances that take values between 0-1 based on different choices of correlation.

```
corDist<-function(x){(1-cor(t(x),method="pearson"))/2}
spearDist<-function(x){(1-cor(t(x),method="spearman"))/2}
```

These distances are defined so as to give distance of 0 between samples with correlation 1, and distance of 1 for correlation -1.

We will also compare using different algorithms for clustering. Currently, `clusterMany` requires that the distances work with all of the `clusterFunction` choices given. Since some of the `clusterFunction` algorithms require a distance matrix between 0-1, this means we can only compare all of the algorithms when the distance is a 0-1 distance. (Future versions may try to create a work around so that the algorithm just skips algorithms that don’t match the distance). Since the distances we defined are between 0-1, however, we can use any algorithm that takes dissimilarities as input.

**Note on 0-1 clustering when `subsample=FALSE`** We would note that the default values of \(\alpha\) in `clusterMany` and `RSEC` for the 0-1 clustering were set with the distance \(D\) the result of subsampling or other concensus summary in mind. In generally, subsampling creates a \(D\) matrix with high similarity for many samples who share a cluster (the proportion of times samples are seen together for well clustered samples can easily be in the .8-.95 range, or even exactly 1). For this reason the default \(\alpha\) is 0.1 which requires distances between samples in the 0.1 range or less (i.e. a similarity in the range of 0.9 or more).

To illustrate this point, we show an example of the \(D\) matrix from subsampling. To do this we make use of the `clusterSingle` which is the workhorse mentioned above that runs a single clustering command directly; it gives the output \(D\) from the sampling in the “coClustering” slot of `ce` when we set `replaceCoCluster=TRUE` (and therefore we save it as a separate object, so that it doesn’t write over the existing “coClustering” slot in `ce`). Note that the result is \(1-p\_{ij}\) where \(p\_{ij}\) is the proportion of times sample \(i\) and \(j\) clustered together.

```
ceSub<-clusterSingle(ce,reduceMethod="mad",nDims=1000,subsample=TRUE,subsampleArgs=list(clusterFunction="pam",clusterArgs=list(k=8)),clusterLabel="subsamplingCluster",mainClusterArgs=list(clusterFunction="hierarchical01",clusterArgs=list(alpha=0.1),minSize=5), replaceCoClustering=TRUE)
plotCoClustering(ceSub,colorScale=rev(seqPal5))
```

We see even here, the default of \(\alpha=0.1\) was perhaps too conservative since only two clusters came out (at leastwith size greater than 5).

However, the distances based on correlation calculated directly on the data, such as we created above, are also often used for clustering expression data directly (i.e. without the subsampling step). But they are unlikely to have dissimilarities as low as seen in subsampling, even for well clustered samples. Here’s a visualization of the correlation distance matrix we defined above (using Spearman’s correlation) on the top 1000 most variable features:

```
dSp<-spearDist(t(transformData(ce,reduceMethod="mad",nFilterDims=1000)))
plotHeatmap(dSp,isSymmetric=TRUE,colorScale=rev(seqPal5))
```

We can see that the choice of \(\alpha\) must be much higher (and we are likely to be more sensitive to it).

Notice to calculate the distance in the above plot, we made use of the `transform` function applied to our `ce` object to get the results of dimensionality reduction. The `transform` function gave us a data matrix back that has been transformed, and also reduced in dimensions, like would be done in our clustering routines. `transform` has similar parameters as seen in `clusterMany`,`makeDendrogram` or `clusterSingle` and is useful when you want to manually apply something to transformed and/or dimensionality reduced data; and you can be sure you are getting the same matrix of data back that the clustering algorithms are using.

**Comparing distance functions with `clusterMany`** Now that we have defined the distances we want to compare in our global environment, we can give these to the argument “distFunction” in `clusterMany`. They should be given as character strings giving the names of the functions. For computational ease for this vignette, we will just choose the dimensionality reduction to be the top 1000 features based on MAD and set K=8 or \(\alpha=0.45\).

Since we haven’t yet calculated “mad” on this object, it hasn’t been calculated yet. `clusterMany` does not let you mix and match between uncalculated and stored filters (or dimensionality reductions), so our first step is to store the mad results. We will save these results as a separate object so as to not disrupt the earlier workflow.

```
ceDist<-makeFilterStats(ce,filterStats="mad")
ceDist
```

```
## class: ClusterExperiment 
## dim: 7069 65 
## reducedDimNames: PCA 
## filterStats: var var_makeConsensus.final mad 
## -----------
## Primary cluster type: mergeClusters 
## Primary cluster label: mergeClusters 
## Table of clusters (of primary clustering):
## -1 m1 m2 m3 m4 
##  6 15 14 13 17 
## Total number of clusterings: 41 
## Dendrogram run on 'makeConsensus,final' (cluster index: 2)
## -----------
## Workflow progress:
## clusterMany run? Yes 
## makeConsensus run? Yes 
## makeDendrogram run? Yes 
## mergeClusters run? Yes
```

```
ceDist<-clusterMany(ceDist, k=7:9, alpha=c(0.35,0.4,0.45), 
     clusterFunction=c("tight","hierarchical01","pam","hierarchicalK"),
     findBestK=FALSE,removeSil=c(FALSE),dist=c("corDist","spearDist"),
          reduceMethod=c("mad"),nFilterDims=1000,run=TRUE)
clusterLabels(ceDist)<-gsub("clusterFunction","alg",clusterLabels(ceDist))
clusterLabels(ceDist)<-gsub("Dist","",clusterLabels(ceDist))
clusterLabels(ceDist)<-gsub("distFunction","dist",clusterLabels(ceDist))
clusterLabels(ceDist)<-gsub("hierarchical","hier",clusterLabels(ceDist))
par(mar=c(1.1,15.1,1.1,1.1))
plotClusters(ceDist,axisLine=-2,colData=c("Biological_Condition"))
```

Notice that using the “tight” methods did not give relevant results (no samples were clustered)

### 6.1.5 Example using a user-defined clustering algorithm

Here, we show how to use a user-defined clustering algorithm in `clusterSingle`. Our clustering algorithm will be a simple nearest-neighbor clustering.

To do so, we need to create a `ClusterFunction` object that defines our algorithm. `ClusterFunction` objects recognize clustering algorithms of two different types, based on the required input from the user: `01` or `K` (see (ClusterFunction)[#ClusterFunction] section above for more). Type `K` refers to a clustering algorithm where the user must specify the number of clusters as input parameter, and this is the type of algorithm we will implement (though as we’ll see, in fact our clustering algorithm doesn’t have the user specify the number of clusters…).

First, we need to define a wrapper function that performs the clustering. Here, we define a simple shared nearest-neighbor clustering using functions from the `scran` and the `igraph` packages.

```
library(scran)
library(igraph)
SNN_wrap <- function(x, k, steps = 4, ...) {
  snn <- buildSNNGraph(x, k = k, d = NA, transposed = FALSE) ##scran package
  res <- cluster_walktrap(snn, steps = steps) #igraph package
  return(res$membership)
}
```

Here the argument `k` defines the number of nearest-neighbors to use in constructing the nearest neighbor graph.

To create a type `K` algorithm, the wrapper must have two required arguments:

1. an argument for the input data. This can be `x` if the input is a matrix of \(N\) samples on the columns and features on the rows, or `diss` if the input is expected to be a \(NxN\) dissimilarity matrix. Both `x` and `diss` can be given as parameters if the algorithm handles either one)
2. a parameter `k` specifying the number of clusters (or any other integer-valued parameter that the clustering relies on)

Our `k` value for `SNN_wrap` will not in fact specify the number of clusters, but that is not actually required anywhere. But setting it up as type `K` mainly distinguishes it from the `01` type (which expects a dissimilarity matrix taking values between 0 and 1 in its dissimilarity entries, as input). Also setting it up as type `K` allows us to use the `findBestK` option, where a range of `k` values is tried and those with the best results (in silhouette width) is reported.

Our wrapper function should return a *integer* vector corresponding to the cluster assignments of each sample (see `?ClusterFunction` for information about other types of output available).

`clusterExperiment` provides the function `internalFunctionCheck` that validates user-defined cluster functions. Among other things, it checks that the input and output are compatible with the `clusterExperiment` workflow (see `?internalFunctionCheck` for details). The call to `internalFunctionCheck` contains, in addition to the function definition, arguments specifying information about the type of input, the type of algorithm, and type of output expected by the function. This information is passed to `clusterMany` and `clusterSingle` so that they know what to pass and what to expect from the user-defined method.

```
internalFunctionCheck(SNN_wrap, inputType = "X", algorithmType = "K",
                      outputType="vector")
```

```
## [1] TRUE
```

If it passes all checks (returns `TRUE`), we can then create an object of the S4 class `ClusterFunction` to be used within the package using this same set of arguments. If it fails, it will return a character string giving the error. Among the checks is running the function on a small randomly generated set of data, so the errors may not be about the format of the function, but also whether the series of code runs.

Since we passed the checks, we are ready to define our `ClusterFunction` object.

```
SNN <- ClusterFunction(SNN_wrap, inputType = "X", algorithmType = "K",
                     outputType="vector")
```

Now that we have our object `SNN`, we can treat our custom method as a base clustering routine to be used in `clusterMany`, similarly to how we used `kmeans` and `pam` earlier. However, unlike before, you should pass the actual object `SNN`, and not a quoted version (i.e. not `"SNN"`).

In this example, we use `clusterSingle` to implement a subsample clustering based on SNN. `clusterSingle` is useful if you just want to create a single clustering.

We give `SNN` to the `subsampleArgs` that will be passed to the subsampling. (Note that to create a consensus clustering from the different subsamplings we use a different function, `"hierarchical01"`, that is passed to `mainClusterArgs`),

```
ceCustom <- clusterSingle(
  ce, reduceMethod="PCA", nDims=50,
  subsample = TRUE, sequential = FALSE,
  mainClusterArgs = list(clusterFunction = "hierarchical01",
                         clusterArgs = list(alpha = 0.3),
                         minSize = 1),
  subsampleArgs = list(resamp.num=100,
                       samp.p = 0.7,
                       clusterFunction = SNN,
                       clusterArgs = list(k = 10),
                       ncores = 1)
)
ceCustom
```

```
## class: ClusterExperiment 
## dim: 7069 65 
## reducedDimNames: PCA 
## filterStats: var var_makeConsensus.final 
## -----------
## Primary cluster type: clusterSingle 
## Primary cluster label: clusterSingle 
## Table of clusters (of primary clustering):
##  1  2  3  4  5 
## 30 20 13  1  1 
## Total number of clusterings: 42 
## Dendrogram run on 'makeConsensus,final' (cluster index: 2)
## -----------
## Workflow progress:
## clusterMany run? Yes 
## makeConsensus run? Yes 
## makeDendrogram run? Yes 
## mergeClusters run? Yes
```

Similarly, we can use `clusterMany` to compute clusters using many different methods, including built-in and custom functions. To mix and match built-in functions, you need to get the actual `ClusterFunction` objects that match their names, using the `getBuiltInFunction` function.

```
clFuns<-getBuiltInFunction(c("pam","kmeans"))
```

Then we will add our function to the list of functions. Note that it is important we give a name to every element of the list, including our new function!

```
## currently does not work
clFuns<-c(clFuns, "SNN"=SNN)
```

Now we can give this list of functions to `clusterMany`

```
ceCustom <- clusterMany(ce, dimReduce="PCA",nPCADims=50,
                        clusterFunction=clFuns,
                        ks=4:15, findBestK=FALSE)
```

Note that if I call `getBuiltInFunction` for only one cluster, it returns the actual `ClusterFunction` object, not a list of length 1. To combine it with other functions you need to make it into a list.

### 6.1.6 Dealing with large numbers of clusterings

A good first check before running `clusterMany` is to determine how many clusterings you are asking for. `clusterMany` has some limited internal checks to not do unnecessary duplicates (e.g. `removeSil` only works with some clusterFunctions so `clusterMany` would detect that), but generally takes all combinations. This can take a while for more complicated clustering techniques, so it is a good idea to check what you are getting into. You can do this by running `clusterMany` with `run=FALSE`.

In the following we consider expanding our original clustering choices to consider individual choices of \(K\) (rather than just `findBestK=TRUE`).

```
checkParam<-clusterMany(se, clusterFunction="pam", ks=2:10,
                        removeSil=c(TRUE,FALSE), isCount=TRUE,
                        reduceMethod=c("PCA","var"),
                        nFilterDims=c(100,500,1000),nReducedDims=c(5,15,50),run=FALSE)
dim(checkParam$paramMatrix) #number of rows is the number of clusterings
```

```
## [1] 108  14
```

Each row of the matrix `checkParam$paramMatrix` is a requested clustering (the columns indicate the value of a possible parameter). Our selections indicate 108 different clusterings (!).

We can set `ncores` argument to have these clusterings done in parallel. If `ncores>1`, the parallelization is done via `mclapply` and should not be done in the Rgui interface (see help pages for `mclapply`).

## 6.2 Create a unified cluster from many clusters with `makeConsensus`

After creating many clusterings, `makeConsensus` finds a single cluster based on what samples were in the same clusters throughout the many clusters found by `clusterMany`. While subsampling the data helps be robust to outlying samples, combining across many clustering parameters can help be robust to choice in parameters, particularly when the parameters give roughly similar numbers of clusters.

As mentioned in the Quick Start section, the default option for `makeConsensus` is to only define a cluster when *all* of the samples are in the same clusters across *all* clusterings. However, this is generally too conservative and just results in most samples not being assigned to a cluster.

Instead `makeConsensus` has a parameter `proportion` that governs in what proportion of clusterings the samples should be together. Internally, `makeConsensus` makes a coClustering matrix \(D\). Like the \(D\) created by subsampling in `clusterMany`, the coClustering matrix takes on values 0-1 for the proportion of times the samples are together in the clustering. This \(D\) matrix is saved in the `ce` object and can be visualized with `plotCoClustering` (which is just a call to `plotHeatmap`). Recall the one we last made in the QuickStart, with our last call to `makeConsensus` (`proportion=0.7` and `minSize=3`).

```
plotCoClustering(ce)
```

`makeConsensus` performs the clustering by running a “01” clustering algorithm on the \(D\) matrix of percentage co-clustering (the default being “hierarchical01”). The `alpha` argument to the 01 clustering is `1-proportion`. Also passed to the clustering algorithm is the parameter `minSize` which sets the minimum size of a cluster.

**Treatment of Unclustered assignments** -1 values are treated separately in the calculation. In particular, they are not considered in the calculation of percentage co-clustering – the percent co-clustering is taken only with respect to those clusterings where both samples were assigned. However, a post-processing is done to the clusters found from running the clustering on the \(D\) matrix. For each sample, the percentage of times that they were marked -1 in the clusterings is calculated. If this percentage is greater than the argument `propUnassigned` then the sample is marked as -1 in the clustering returned by `makeConsensus`.

**Good scenarios for running `makeConsensus`** Varying certain parameters result in clusterings better for `makeConsensus` than other sets of parameters. In particular, if there are huge discrepancies in the set of clusterings given to `makeConsensus`, the results will be a shattering of the samples into many small clusters. Similarly, if the number of clusters \(K\) is very different, the end result will likely be like that of the large \(K\), and how much value that is (rather than just picking the clustering with the largest \(K\)), is debatable. However, for “01” clustering algorithms or clusterings using the sequential algorithm, varying the underlying parameters \(\alpha\) or \(k\_0\) often results in roughly similar clusterings across the parameters so that creating a consensus across them is highly informative.

### 6.2.1 Consensus from subsets of clusterings

A call to `clusterMany` or to `RSEC` can generate many clusterings that result from changing the underlying parameters of a given method (e.g., the number of centers in k-means), the dimensionality reduction, the distance function, or the base algorithm used for the clustering (e.g., PAM vs. k-means).

To highlight interesting structure in the data, it may be useful to understand whether a set of samples tends to cluster together across paramter choices of the same method or across very different methods.

`clusterExperiment` makes it easy to extract a subset of clusterings and to compute a consensus clustering for any given subset, to help addressing this type of questions, as we have already seen in the section on `makeConsensus`.

As an example, assume that we want to explore the role of PCA in the clustering results. We can separately calculate a consensus of those clusterings that used 15 or 50 principal components of the data.

First, we use the `getClusterManyParams` function to extract the information on the clusterings performed.

```
params <- getClusterManyParams(ce)
head(params)
```

```
##                                                       clusteringIndex
## reduceMethod=PCA,nReducedDims=5,nFilterDims=NA,k=5                  5
## reduceMethod=var,nReducedDims=NA,nFilterDims=100,k=5                6
## reduceMethod=PCA,nReducedDims=15,nFilterDims=NA,k=5                 7
## reduceMethod=PCA,nReducedDims=50,nFilterDims=NA,k=5                 8
## reduceMethod=var,nReducedDims=NA,nFilterDims=500,k=5                9
## reduceMethod=var,nReducedDims=NA,nFilterDims=1000,k=5              10
##                                                       reduceMethod
## reduceMethod=PCA,nReducedDims=5,nFilterDims=NA,k=5             PCA
## reduceMethod=var,nReducedDims=NA,nFilterDims=100,k=5           var
## reduceMethod=PCA,nReducedDims=15,nFilterDims=NA,k=5            PCA
## reduceMethod=PCA,nReducedDims=50,nFilterDims=NA,k=5            PCA
## reduceMethod=var,nReducedDims=NA,nFilterDims=500,k=5           var
## reduceMethod=var,nReducedDims=NA,nFilterDims=1000,k=5          var
##                                                       nReducedDims
## reduceMethod=PCA,nReducedDims=5,nFilterDims=NA,k=5               5
## reduceMethod=var,nReducedDims=NA,nFilterDims=100,k=5            NA
## reduceMethod=PCA,nReducedDims=15,nFilterDims=NA,k=5             15
## reduceMethod=PCA,nReducedDims=50,nFilterDims=NA,k=5             50
## reduceMethod=var,nReducedDims=NA,nFilterDims=500,k=5            NA
## reduceMethod=var,nReducedDims=NA,nFilterDims=1000,k=5           NA
##                                                       nFilterDims k
## reduceMethod=PCA,nReducedDims=5,nFilterDims=NA,k=5             NA 5
## reduceMethod=var,nReducedDims=NA,nFilterDims=100,k=5          100 5
## reduceMethod=PCA,nReducedDims=15,nFilterDims=NA,k=5            NA 5
## reduceMethod=PCA,nReducedDims=50,nFilterDims=NA,k=5            NA 5
## reduceMethod=var,nReducedDims=NA,nFilterDims=500,k=5          500 5
## reduceMethod=var,nReducedDims=NA,nFilterDims=1000,k=5        1000 5
```

We can select only the clusterings in which we are interested in and pass them to `makeConsensus` using the `whichClusters` argument.

```
clusterIndices15 <- params$clusteringIndex[which(params$nReducedDims == 15)]
clusterIndices50 <- params$clusteringIndex[which(params$nReducedDims == 50)]
#note, the indices will change as we add clusterings!
clusterNames15 <- clusterLabels(ce)[clusterIndices15]
clusterNames50 <- clusterLabels(ce)[clusterIndices50]
shortNames15<-gsub("reduceMethod=PCA,nReducedDims=15,nFilterDims=NA,","",clusterNames15)
shortNames50<-gsub("reduceMethod=PCA,nReducedDims=50,nFilterDims=NA,","",clusterNames50)

ce <- makeConsensus(ce, whichClusters = clusterNames15, proportion = 0.7,
                       clusterLabel = "consensusPCA15")
ce <- makeConsensus(ce, whichClusters = clusterNames50, proportion = 0.7,
                       clusterLabel = "consensusPCA50")
```

Analogously, we’ve seen that many visualization functions have a `whichClusters` argument that can be used to visually inspect the similarities and differences between subsets of clusterings.

Here we show using this features with `plotClustersWorkflow` for the two different consensus clusterings we made.

```
par(mar=plotCMar,mfrow=c(1,2))
plotClustersWorkflow(ce, whichClusters ="consensusPCA15",clusterLabel="Consensus",whichClusterMany=match(clusterNames15,clusterLabels(ceSub)),clusterManyLabels=c(shortNames15),axisLine=-1,nBlankLines=1,main="15 PCs")
plotClustersWorkflow(ce, whichClusters = c("consensusPCA50"),clusterLabel="Consensus",clusterManyLabels=shortNames50, whichClusterMany=match(clusterNames50,clusterLabels(ceSub)),nBlankLines=1,main="50 PCs")
```

We can also choose a subset and vary the parameters

```
wh<-getClusterManyParams(ce)$clusteringIndex[getClusterManyParams(ce)$reduceMethod=="var"]
ce<-makeConsensus(ce,whichCluster=wh,proportion=0.7,minSize=3,
                clusterLabel="makeConsensus,nVAR")
plotCoClustering(ce)
```

We can compare to all of our other versions of `makeConsensus`. While they do not all have `clusterTypes` equal to “makeConsensus” (only the most recent call has clusterType exactly equal to “makeConsensus”), they all have “makeConsensus” as part of their clusterType, even though they have different clusterLabels (and now we’ll see that it was useful to give them different labels!)

```
wh<-grep("makeConsensus",clusterTypes(ce))
par(mar=plotCMar)
plotClusters(ce,whichClusters=rev(wh),axisLine=-1)
```

## 6.3 Creating a Hierarchy of Clusters and Merging clusters

As mentioned above, we find that merging clusters together based on the extent of differential expression between the features to be a useful method for combining many small clusters.

We provide a method for doing this that consists of two steps. Making a hierarchy between the clusterings and then estimating the amount of differential expression at each branch of the hierarchy.

### 6.3.1 makeDendrogram and plotDendrogram

`makeDendrogram` creates a hierarchical clustering of the clusters as determined by the primaryCluster of the `ClusterExperiment` object. In addition to being used for merging clusters, the dendrograms created by `makeDendrogram` are also useful for ordering the clusters in `plotHeatmap` as has been shown above.

`makeDendrogam` performs hierarchical clustering of the cluster medoids (after transformation of the data) and provides a dendrogram that will order the samples according to this clustering of the clusters. The hierarchical ordering of the dendrograms is saved internally in the `ClusterExperiment` object.

Like clustering, the dendrogram can depend on what features are included from the data. The same options for clustering are available for the hierarchical clustering of the clusters, namely choices of dimensionality reduction via `reduceMethod` and the number of dimensions via `nDims`.

```
ce<-makeDendrogram(ce,reduceMethod="var",nDims=500)
plotDendrogram(ce)
```

Notice that the plot of the dendrogram shows the hierarchy of the clusters (and color codes them according to the colors stored in colorLegend slot).

Recall that the most recent clustering made is from our call to `makeConsensus`, where we experimented with using on some of the clusterings from `clusterMany`, so that is our current primaryCluster:

```
show(ce)
```

```
## class: ClusterExperiment 
## dim: 7069 65 
## reducedDimNames: PCA 
## filterStats: var var_makeConsensus.final var_makeConsensus.nVAR 
## -----------
## Primary cluster type: makeConsensus 
## Primary cluster label: makeConsensus,nVAR 
## Table of clusters (of primary clustering):
## -1 c1 c2 c3 c4 c5 c6 c7 
## 10 15 13  9  7  5  3  3 
## Total number of clusterings: 44 
## Dendrogram run on 'makeConsensus,nVAR' (cluster index: 1)
## -----------
## Workflow progress:
## clusterMany run? Yes 
## makeConsensus run? Yes 
## makeDendrogram run? Yes 
## mergeClusters run? No
```

This is the clustering from combining only the clusterings from `clusterMany` that use the top most variable genes. Because it is the primaryCluster, it was the clustering that was used by default to make the dendrogram.

We might prefer to get back to the dendrogram based on our `makeConsensus` in quick start (the “makeConsensus, final” clustering). We’ve lost that dendrogram when we called `makeDendrogram` again. However, we can rerun `makeDendrogram` and choose a different clustering from which to make the dendrogram.

```
ce<-makeDendrogram(ce,reduceMethod="var",nDims=500,
                   whichCluster="makeConsensus,final")
```

We will visualize the dendrogram with `plotDendrogram`. The default setting plots the dendrogram where there are color blocks equal to the size of the clusters (i.e number of samples in each cluster).

```
plotDendrogram(ce,leafType="sample",plotType="colorblock")
```

We can actually use `plotDendrogram` to compare clusterings too, like `plotClusters` using the `whichClusters` argument to identfy which clusters to show. For example, lets compare our different `makeConsensus` results

```
par(mar=plotDMar)
whCM<-grep("makeConsensus",clusterTypes(ce))
plotDendrogram(ce,whichClusters=whCM,leafType="sample",plotType="colorblock")
```

Unlike `plotClusters`, however, there is no aligning of samples to make samples with the same cluster group together.

#### 6.3.1.1 Designating among many clustering results to use for downstream analysis

Note that if we look at the clusterType of the “makeConsensus,final” cluster, it is not of type “makeConsensus”, but “makeConsensus.x”. This is because we’ve run additional `makeConsensus` steps on this data, and the clustering “makeConsensus,final” is not the most current one. So to distinguish old iterations from the most recent run, the “.x” is added to the “clusterType” where “x” indicates what iteration it was (it would have added “.x” to the clusterLabel as well as if we had assigned it our own label)

```
clusterTypes(ce)[which(clusterLabels(ce)=="makeConsensus,final")]
```

```
## [1] "makeConsensus.3"
```

We can choose reset this past call to `makeConsensus` to be the current ‘makeConsensus’ output (which will also set this clustering to be the primaryCluster).

```
ce<-setToCurrent(ce,whichCluster="makeConsensus,final")
show(ce)
```

```
## class: ClusterExperiment 
## dim: 7069 65 
## reducedDimNames: PCA 
## filterStats: var var_makeConsensus.final var_makeConsensus.nVAR 
## -----------
## Primary cluster type: makeConsensus 
## Primary cluster label: makeConsensus,final 
## Table of clusters (of primary clustering):
## -1 c1 c2 c3 c4 c5 c6 c7 
##  6 15 14  9  8  5  4  4 
## Total number of clusterings: 44 
## Dendrogram run on 'makeConsensus,final' (cluster index: 5)
## -----------
## Workflow progress:
## clusterMany run? Yes 
## makeConsensus run? Yes 
## makeDendrogram run? Yes 
## mergeClusters run? No
```

We don’t need to recall `makeDendrogram`, since we already used this clustering to make the dendrogram by our explicit call of the argument `whichCluster`.

### 6.3.2 Merging clusters with little differential expression

We then can use this hierarchy of clusters to merge clusters that show little difference in expression. We do this by testing, for each node of the dendrogram, for which features is the mean of the set of clusters to the right split of the node is equal to the mean on the left split. This is done via the `getBestFeatures` (see section on getBestFeatures), where the `type` argument is set to “Dendro”.

Starting at the bottom of the tree, those clusters that have the percentage of features with differential expression below a certain value (determined by the argument `cutoff`) are merged into a larger cluster. This testing of differences and merging continues until the estimated percentage of non-null DE features is above `cutoff`. This means lower values of `cutoff` result in less merging of clusters. There are multiple methods of estimation of the percentage of non-null features implemented. The option `mergeMethod="adjP"` which we showed earlier is the simplest: the proportion found significant by calculating the proportion of DE genes a given False Discovery Rate threshold of 0.05 (using the Benjamini-Hochberg procedure). However, other more sophisticated methods are also implemented (see `?mergeClusters`).

Notice that `mergeClusters` will always run based on the clustering that made the currently existing dendrogram. So it is always good to check that it is what we expect.

```
ce
```

```
## class: ClusterExperiment 
## dim: 7069 65 
## reducedDimNames: PCA 
## filterStats: var var_makeConsensus.final var_makeConsensus.nVAR 
## -----------
## Primary cluster type: makeConsensus 
## Primary cluster label: makeConsensus,final 
## Table of clusters (of primary clustering):
## -1 c1 c2 c3 c4 c5 c6 c7 
##  6 15 14  9  8  5  4  4 
## Total number of clusterings: 44 
## Dendrogram run on 'makeConsensus,final' (cluster index: 5)
## -----------
## Workflow progress:
## clusterMany run? Yes 
## makeConsensus run? Yes 
## makeDendrogram run? Yes 
## mergeClusters run? No
```

We see in the summary “Dendrogram run on ‘makeConsensus,final’”, showing us that this is the clustering that will be used (and also showing us the value of giving our own labels to the results of `makeConsensus` if we are going to try different strategies).

We will run `mergeClusters` with the option `mergeMethod="adjP"`. We will also set `plotInfo="adjP"` meaning that we would like the `mergeClusters` command to also produce a plot showing the dendrogram and the estimates from the `adjP` method for each node. We also set `calculateAll=FALSE` for illustration purposes, meaning the function will only calculate the estimates for the methods we request, but as we explain below, that is not necessarily the best option if you are going to be trying out different cutoffs.

```
ce<-mergeClusters(ce,mergeMethod="adjP",plotInfo=c("adjP"),calculateAll=FALSE)
```

The info about the merge is saved in the `ce` object.

```
mergeMethod(ce)
```

```
## [1] "adjP"
```

```
mergeCutoff(ce)
```

```
## [1] 0.05
```

```
nodeMergeInfo(ce)
```

```
##    Node                     Contrast isMerged mergeClusterId Storey PC
## 1 Node1 (X2+X5+X4+X7)/4-(X1+X3+X6)/3    FALSE             NA     NA NA
## 2 Node2              X2-(X5+X4+X7)/3    FALSE             NA     NA NA
## 3 Node3                 X1-(X3+X6)/2    FALSE             NA     NA NA
## 4 Node4                        X3-X6     TRUE              3     NA NA
## 5 Node5                 X5-(X4+X7)/2     TRUE              4     NA NA
## 6 Node6                        X4-X7     TRUE             NA     NA NA
##         adjP locfdr MB JC
## 1 0.12137502     NA NA NA
## 2 0.09478003     NA NA NA
## 3 0.05658509     NA NA NA
## 4 0.01938039     NA NA NA
## 5 0.03437544     NA NA NA
## 6 0.02447305     NA NA NA
```

Notice that `nodeMergeInfo` gives for each node the proportion estimated to be differentially expressed at each node (as displayed in the plot that we requested), as well as whether that node was merged together in the `mergeClusters` call (the `isMerged` column). Because we set `calculateAll=FALSE` only the methods needed for our command were calculated (`adjP`). The others have `NA` values. The column `mergeClusterId` tells us which nodes in the tree are now equivalent to a cluster; this is different than the `isMerged` column, since some nodes can be merged but if their parent nodes were also merged, then that node will not be equivalent to a cluster in the “mergeClusters” clustering.

`mergeClusters` can also be run without merging the cluster, and simply drawing a plot showing the dendrogram along with the estimates of the percentage of non-null features to aid in deciding a cutoff and method. By setting `plotInfo="all"`, all of the estimates of the different methods are displayed simultaneously, while before we only showed the values for the specific `mergeMethod` we requested.

```
ce<-mergeClusters(ce,mergeMethod="none",plotInfo="all")
```

Notice that now if we call `nodeMergeInfo`, all of the methods now have estimates.

```
nodeMergeInfo(ce)
```

```
##    Node                     Contrast isMerged mergeClusterId    Storey
## 1 Node1 (X2+X5+X4+X7)/4-(X1+X3+X6)/3       NA             NA 0.4400905
## 2 Node2              X2-(X5+X4+X7)/3       NA             NA 0.4058566
## 3 Node3                 X1-(X3+X6)/2       NA             NA 0.3526666
## 4 Node4                        X3-X6       NA             NA 0.2477012
## 5 Node5                 X5-(X4+X7)/2       NA             NA 0.4115151
## 6 Node6                        X4-X7       NA             NA 0.2295940
##          PC       adjP locfdr        MB JC
## 1 0.3780116 0.12137502     NA 0.4041590 NA
## 2 0.3512809 0.09478003     NA 0.3702080 NA
## 3 0.2905322 0.05658509     NA 0.3131985 NA
## 4 0.1886942 0.01938039     NA 0.2138916 NA
## 5 0.3429098 0.03437544     NA 0.3760079 NA
## 6 0.2254791 0.02447305     NA 0.2837742 NA
```

This means in any future calls to `mergeClusters` there will be no more need for calculations of per-gene significance, which will speed up the calls if you just want to change the cutoff (all of the methods used the same input of per-gene p-values, so recalculating them each time is computationally inefficient). In practice, the default is `calculateAll=TRUE`, meaning all methods are calculated unless the user specifically requests otherwise.

Now we can pick a cutoff and rerun `mergeClusters`. We’ll give it a label to keep it separate from the previous merge clusters run we had made. Note, we can turn off plotting completely by setting `plot=FALSE`.

```
ce<-mergeClusters(ce,cutoff=0.05,mergeMethod="adjP",clusterLabel="mergeClusters,v2",plot=FALSE)
ce
```

```
## class: ClusterExperiment 
## dim: 7069 65 
## reducedDimNames: PCA 
## filterStats: var var_makeConsensus.final var_makeConsensus.nVAR 
## -----------
## Primary cluster type: mergeClusters 
## Primary cluster label: mergeClusters,v2 
## Table of clusters (of primary clustering):
## -1 m1 m2 m3 m4 
##  6 15 14 13 17 
## Total number of clusterings: 46 
## Dendrogram run on 'makeConsensus,final' (cluster index: 7)
## -----------
## Workflow progress:
## clusterMany run? Yes 
## makeConsensus run? Yes 
## makeDendrogram run? Yes 
## mergeClusters run? Yes
```

Notice that the `nodeMergeInfo` has changed, since different nodes were merged, but the estimates per node stay the same.

```
nodeMergeInfo(ce)
```

```
##    Node                     Contrast isMerged mergeClusterId    Storey
## 1 Node1 (X2+X5+X4+X7)/4-(X1+X3+X6)/3    FALSE             NA 0.4400905
## 2 Node2              X2-(X5+X4+X7)/3    FALSE             NA 0.4058566
## 3 Node3                 X1-(X3+X6)/2    FALSE             NA 0.3526666
## 4 Node4                        X3-X6     TRUE              3 0.2477012
## 5 Node5                 X5-(X4+X7)/2     TRUE              4 0.4115151
## 6 Node6                        X4-X7     TRUE             NA 0.2295940
##          PC       adjP locfdr        MB JC
## 1 0.3780116 0.12137502     NA 0.4041590 NA
## 2 0.3512809 0.09478003     NA 0.3702080 NA
## 3 0.2905322 0.05658509     NA 0.3131985 NA
## 4 0.1886942 0.01938039     NA 0.2138916 NA
## 5 0.3429098 0.03437544     NA 0.3760079 NA
## 6 0.2254791 0.02447305     NA 0.2837742 NA
```

If we want to rerun `mergeClusters` with a different method, we can do that instead.

```
ce<-mergeClusters(ce,cutoff=0.15,mergeMethod="MB",
                  clusterLabel="mergeClusters,v3",plot=FALSE)
ce
```

```
## class: ClusterExperiment 
## dim: 7069 65 
## reducedDimNames: PCA 
## filterStats: var var_makeConsensus.final var_makeConsensus.nVAR 
## -----------
## Primary cluster type: mergeClusters 
## Primary cluster label: mergeClusters,v3 
## Table of clusters (of primary clustering):
## -1 m1 m2 m3 m4 m5 m6 m7 
##  6 15 14  9  8  5  4  4 
## Total number of clusterings: 47 
## Dendrogram run on 'makeConsensus,final' (cluster index: 8)
## -----------
## Workflow progress:
## clusterMany run? Yes 
## makeConsensus run? Yes 
## makeDendrogram run? Yes 
## mergeClusters run? Yes
```

We can use `plotDendrogram` to compare the results. Notice that `plotDendrogram` can recreate the above plots that were created in the calls to `mergeClusters` via the argument `mergeInfo` (of course, this only works *after* `mergeClusters` has actually been called so that the information is saved in the `ce` object).

```
par(mar=c(1.1,1.1,6.1,2.1))
plotDendrogram(ce,whichClusters=c("mergeClusters,v3","mergeClusters,v2"),mergeInfo="mergeMethod")
```

#### 6.3.2.1 Requiring a certain log-fold change

With a large number of cells, it can be overly easy to get significant results, even if the size of the differences is small. Another reasonable constraint is to require the difference between the contrasts to be at least of a certain fold-change for the gene to be counted as different. We allow this option for the merge method `adjP`. Namely, the proportion significant calculated at each node requires both an adjusted p-value less than 0.05 but also that the estimated \(log\_2\) fold-change have an absolute value greater than an amount specified by the argument `logFCCutoff`.

```
ce<-mergeClusters(ce,cutoff=0.05,mergeMethod="adjP", logFCcutoff=2,
                  clusterLabel="mergeClusters,FC1",plot=FALSE)
ce
```

```
## class: ClusterExperiment 
## dim: 7069 65 
## reducedDimNames: PCA 
## filterStats: var var_makeConsensus.final var_makeConsensus.nVAR 
## -----------
## Primary cluster type: mergeClusters 
## Primary cluster label: mergeClusters,FC1 
## Table of clusters (of primary clustering):
## -1 m1 m2 m3 m4 
##  6 15 14 13 17 
## Total number of clusterings: 48 
## Dendrogram run on 'makeConsensus,final' (cluster index: 9)
## -----------
## Workflow progress:
## clusterMany run? Yes 
## makeConsensus run? Yes 
## makeDendrogram run? Yes 
## mergeClusters run? Yes
```

In this case, we can see that it did not make a difference in the merging.

```
par(mar=c(1.1,1.1,6.1,2.1))
plotDendrogram(ce,whichClusters=c("mergeClusters,FC1","mergeClusters,v3","mergeClusters,v2"),mergeInfo="mergeMethod")
```

## 6.4 Keeping track of and rerunning elements of the workflow

The commands we have shown above show a workflow which continually saves the results over the previous object, so that additional information just gets added to the existing object.

What happens if some parts of the clustering workflow are re-run? For example, in the above we reran parts of the workflow when we talked about them in more detail, or to experiment with parameter settings.

The workflow commands check for existing clusters of the workflow (based on the `clusterTypes` of the clusterings). If there exist clusterings from previous runs *and* such clusterings came from calls that are “downstream” of the requested clustering, then the method will change their clusterTypes value by adding a “.i”, where \(i\) is a numerical index keeping track of replicate calls.

For example, suppose we rerun ‘makeConsensus’, say with a different parameter choice of the proportion similarity to require. Then `makeConsensus` searches the existing clusterings in the input object. Any existing `makeConsensus` results will have their `clusterTypes` changed from `makeConsensus` to `makeConsensus.x`, where \(x\) is the largest such number needed to be greater than any existing `makeConsensus.x` (after all, you might do this many times!). Their labels will also be updated if they just have the default label, but if the user has given different labels to the clusters those will be preserved.

Moreover, since `mergeClusters` is downstream of `makeConsensus` in the workflow, currently existing `mergeClusters` will also get bumped to `mergeClusters.x`. However, `clusterMany` is upstream of `makeConsensus` (i.e. you expect there to be existing `clusterMany` before you run `makeConsensus`) so nothing will happen to `clusterMany`.

This is handled internally, and may never be apparent to the user unless they choose `whichClusters="all"` in a plotting command. Indeed this is one reason to always pick `whichClusters="workflow"`, so that these saved previous versions are not displayed.

However, if the user wants to “go back” to previous versions and make them the current iteration, we have seen that the `setToCurrent` command will do this. `setToCurrent` follows the same process as described above, only with an existing cluster set to the current part of the pipeline.

Note that there is nothing that governs or protects the `clusterTypes` values to be of a certain kind. This means that if the user decides to name a clusterTypes of a clustering one of these protected names, that is allowed. However, it could clearly create some havoc if done poorly.

**Erasing old clusters** You can also choose to have **all** old versions erased by choosing the options `eraseOld=TRUE` in the call to `clusterMany`, `makeConsensus`,`mergeClusters` and/or `setToCurrent`. `eraseOld=TRUE` in any of these functions will delete ALL past workflow results except for those that are both in the current workflow *and* “upstream” of the requested command. You can also manually remove clusters with `removeClusters`.

**Finding workflow iterations** Sometimes which numbered iteration a particular call is in will not be obvious if there are many calls to the workflow. You may have a `mergeClusters.2` cluster but no `mergeClusters.1` because of an upstream workflow call in the middle that bumped the iteration value up to 2 without ever making a `mergeClusters.1`. If you really want to, you can see more about the existing iterations and where they are in the `clusterMatrix`. “0” refers to the current iteration; otherwise the smaller the iteration number, the earlier it was run.

```
workflowClusterTable(ce)
```

```
##                Iteration
## Type             0  1  2  3  4  5  6  7  8
##   final          0  0  0  0  0  0  0  0  0
##   mergeClusters  1  0  0  1  0  0  1  1  1
##   makeConsensus  1  1  1  0  1  1  1  0  0
##   clusterMany   36  0  0  0  0  0  0  0  0
```

Explicit details about every workflow cluster and their index in `clusterMatrix` is given by `workflowClusterDetails`:

```
head(workflowClusterDetails(ce),8)
```

```
##   index          type iteration              label
## 1     1 mergeClusters         0  mergeClusters,FC1
## 2     2 mergeClusters         8   mergeClusters,v3
## 3     3 mergeClusters         7   mergeClusters,v2
## 4     4 mergeClusters         6    mergeClusters.6
## 5     5 makeConsensus         6 makeConsensus,nVAR
## 6     6 makeConsensus         5     consensusPCA50
## 7     7 makeConsensus         4     consensusPCA15
## 8     8 mergeClusters         3    mergeClusters.3
```

**A note on the `whichCluster` argument** Many functions take the `whichCluster` argument for identifying a clustering or clusterings on which to perform an action. These arguments all act similarly across functions, and allow the user to give character arguments. As described above, these can be shortcuts like “workflow”, or they can match either clusterTypes or clusterLabels of the object. It is important to note that matching is first done to clusterTypes, and then if not successful to clusterLabels. Since neither clusterTypes nor clusterLabels is guaranteed to be unique, the user should be careful in how they make the call. And, of course, `whichCluster` arguments can also take explicit numeric integers that identify the column(s) of the clusterMatrix that should be used.

### 6.4.1 Designate a Final Clustering

A final protected clusterTypes is “final”. This is not created by any method, but can be set to be the clusterType of a clustering by the user (via the `clusterTypes` command). Any clustering marked `final` will be considered one of the workflow values for commands like `whichClusters="workflow"`. However, they will NOT be renamed with “.x” or removed if `eraseOld=TRUE`. This is a way for a user to ‘save’ a clustering as important/final so it will not be changed internally by any method, yet still have it show up with the “workflow” clustering results. There is no limit to the number of such clusters that are so marked, but the utility of doing so will drop if too many such clusters are chosen.

For best functionality, particularly if a user has determined a single final clustering after completing clustering, a user will probably want to set the primaryClusterIndex to be that of the final cluster and rerun makeDendrogram. This will help in plotting and visualizing. The `setToFinal` command does this.

Here we will demonstrate marking a cluster as final. We go back to our previous mergeClusters that we found with `cutoff=0.05` and mark it as our final clustering. First we need to find which cluster it is. We see from our above call to the workflow functions above, that it is clusterType equal to “mergeClusters.4” and label equal to “mergeClusters,v2”. In our call to `setToFinal` we will decide to change it’s label as well.

```
ce<-setToFinal(ce,whichCluster="mergeClusters,v2",
               clusterLabel="Final Clustering")
par(mar=plotCMar)
plotClusters(ce,whichClusters="workflow")
```

Note that because it is labeled as “final” it shows up automatically in “workflow” clusters in our `plotClusters` plot. It has also been set as our primaryCluster and has the new clusterLabel we gave it in the call to `setToFinal`.

This didn’t get rid of our undesired `mergeClusters` result that is most recent. It still shows up as “the” mergeClusters result. This might be undesired. We could remove that “mergeClusters” result with `removeClusters`. Alternatively, we could manually change the clusterTypes to `mergeClusters.x` so that it doesn’t show up as current. A cleaner way to do this would have been to first set the desired cluster (“mergeClusters.4”) to the most current iteration with `setToCurrent`, which would have bumped up the existing `mergeClusters` result to be no longer current.

## 6.5 RSEC

`RSEC` is a single function that follows the entire workflow described above, but makes the choices to set `subsample=TRUE` and `sequential=TRUE` to provide more robust clusterings. This removes a number of options from clusterMany, making for a slightly reduced set of arguments. `RSEC` also implements the `makeConsensus`, `makeDendrogram` and `mergeClusters` steps, again with not all the arguments available to those function to be set by the user, only the most common. Furthermore, the defaults set in `RSEC` are those we choose for our algorithm, and occassionally vary from stand-alone method. The final output is a `ClusterExperiment` object as you would get from following the workflow.

We give the following correspondence to help see what arguments of each component are fixed by RSEC, and which are allowed to be set by the user (as well as their correspondence to arguments in the workflow functions).

|  | Arguments in original function internally fixed | Arguments in RSEC for the user |  |  |
| --- | --- | --- | --- | --- |
|  | *Name of Argument in original function (if different)* | *Notes* |
| *clusterMany* | sequential=TRUE | k0s | ks | RSEC only sets ‘k0’, no other k |
| - | distFunction=NA | clusterFunction |  |
| - | removeSil=FALSE | reduceMethod |  |
| - | subsample=TRUE | nFilterDims |  |
| - | silCutoff=0 | nReducedDims |  |
| - |  | alphas |  |
| - |  | betas |  |
| - |  | minSizes |  |
| - |  | mainClusterArgs |  |
| - |  | subsampleArgs |  |
| - |  | seqArgs |  |
| - |  | run |  |
| - |  | ncores |  |
| - |  | random.seed |  |
| - |  | isCount |  |
| - |  | transFun |  |
| - |  | isCount |  |
| *makeConsensus* | propUnassigned = *(default)* | consensusProportion | proportion |
| - | consensusMinSize | minSize |  |
| *makeDendrogram* | filterIgnoresUnassigned=TRUE | dendroReduce | reduceMethod |
| - | unassignedSamples= *(default)* | dendroNDims | nDims |
| *mergeClusters* | plot=FALSE | mergeMethod |  |
| - |  | mergeCutoff | cutoff |
| - |  | mergeDEMethod | DEMethod |
| - |  | mergeLogFCcutoff | logFCcutoff |

# 7 Finding Features related to a Clustering

The function `getBestFeatures` finds features in the data that are strongly differentiated between the clusters of a given clustering. Finding the best features is generally the last step in the workflow, once a final clustering has been decided upon, though as we have seen it is also called internally in `mergeClusters` to decide between which clusters to merge together.

The function `getBestFeatures` calls either `limma` (Smyth 2004, Ritchie et al. (2015)) or `edgeR` (Robinson, Mccarthy, and Smyth 2010) on input data to determine the gene features most associated with a particular clustering. `getBestFeatures` picks the `primaryCluster` of a `ClusterExperiment` object as the clustering to use to find features. If the standard workflow is followed, this will be the last completed step (usually the result of `mergeClusters` or manually choosing a final cluster via `setToFinal`). The primaryCluster can of course be changed by setting `primaryClusterIndex` to point to a different clustering.

The basic implementation of these functions fits a linear model per feature and tests for the significance of parameters of that linear model, with appropriate adjustment to a negative binomial model in the case of `edgeR`. The main contribution of `getBestFeatures` is to interface with `limma` or `edgeR` so as to pick appropriate parameters or tests for comparing clusters. Naturally, `getBestFeatures` also seamlessly works with `ClusterExperiment` objects to minimize the burden on the user. The output is in the form of `topTable` or `topTags` in `limma` or `edgeR` respectively, i.e. a data.frame giving the relevant features, the p-value, etc.

Note that `getBestFeatures` will remove all samples unassigned to a cluster (i.e. `-1` or `-2`), so that these samples will not in anyway influence the DE analysis (see [#unassigned] for more information about unassigned samples).

## 7.1 Types of Significance Tests

There are several choices of what is the most appropriate test to determine whether a feature is differentially expressed across the clusterings. All of these methods first fit a linear model where the clusters categories of the clustering is the explanatory factor in the model (samples with -1 or -2 are ignored). The methods differ only in what significance tests they then perform, which is controlled by the argument `type`. By default, `getBestFeatures` finds significant genes based on a F-test between the clusters (`type="F"`). This is a very standard test to compare clusters, which is why it is the default, however it may not be the one that gives the best or most specific results. Indeed, in our “Quick Start”, we did not use the \(F\) test, but rather all pair-wise comparisons between the clusters.

The \(F\) test is a test for whether there are *any* differences in expression between the clusters for a feature. Three other options are available that try to detect instead specific kinds of differences between clusters that might be of greater interest. Specifically, these differences are encoded as “contrasts”, meaning specific types of differences between the means of clusters.

Note that for all of these contrasts, we are making use of all of the data, not just the samples in the particular cluster pairs being compared. This means the variance is estimated with all the samples. Indeed, the same linear model is being used for all of these comparisons.

### 7.1.1 All Pairwise

The option `type="Pairs"`, which we saw earlier, performs all pair-wise tests between the clusters for each feature, testing for each pair of clusters whether the mean of the feature is different between the two clusters. Here is the example from above using all pairwise comparisons on the results of rsec:

```
pairsAllTop<-getBestFeatures(rsecFluidigm,contrastType="Pairs",DEMethod="edgeR",p.value=0.05)
dim(pairsAllTop)
```

```
## [1] 100   8
```

```
head(pairsAllTop)
```

```
##   IndexInOriginal  Contrast Feature     logFC   logCPM        LR
## 1            1465 Cl01-Cl02    DLK1  13.90356 8.804668 172.58983
## 2            5044 Cl01-Cl02  RPS4Y1  12.51876 7.424298 119.76806
## 3            2291 Cl01-Cl02    GPC3  12.21939 7.126492 110.84635
## 4            3788 Cl01-Cl02    NNAT -13.34129 8.751824 110.37000
## 5            3985 Cl01-Cl02    OTX2  11.80412 6.714252  87.07782
## 6            1640 Cl01-Cl02   EFNA5  11.35424 8.430487  84.19065
##        P.Value    adj.P.Val
## 1 2.011624e-39 1.422017e-34
## 2 7.110712e-28 2.513281e-23
## 3 6.393907e-26 9.579242e-22
## 4 8.130634e-26 9.579242e-22
## 5 1.043343e-20 9.219237e-17
## 6 4.492896e-20 3.528920e-16
```

Notice that compared to the quick start guide, we didn’t set the parameter `number` which is passed to topTable, so we can get out *at most* 10 significant features for each contrast/comparison (because the default value of `number` in `topTable` is 10). Similarly, if we didn’t set a value for `p.value`, `topTable` would return the top `number` genes per contrast, regardless of whether they were all significant or not. These are the defaults of `topTable`, which we purposefully do not modify, but we urge the user to read the documentation of `topTable` carefully to understand what is being asked for. In the QuickStart, we set `number=NROW(rsecFluidigm)` to make sure we got *all* significant genes.

In addition to the columns provided by `topTable`, the column “Contrast” tells us what pairwise contrast the result is from. “Cl01-Cl02” means a comparison of cluster 1 and cluster 2 (note that these refer to the cluster ids, not any name they might have). The column “IndexInOriginal” gives the index of the gene to the original input data matrix, namely `assay(ce)`. The other columns are given by `topTable` (with the column “Feature” renamed – it is usually “ProbeID” in `limma`).

### 7.1.2 One Against All

The choice `type="OneAgainsAll"` performs a comparison of a cluster against the mean of all of the other clusters.

```
best1vsAll<-getBestFeatures(rsecFluidigm,contrastType="OneAgainstAll",DEMethod="edgeR",p.value=0.05,number=NROW(rsecFluidigm))
head(best1vsAll)
```

```
##   IndexInOriginal ContrastName                     Contrast Feature
## 1            3788         Cl01 Cl01-(Cl02+Cl03+Cl04+Cl05)/4    NNAT
## 2            1465         Cl01 Cl01-(Cl02+Cl03+Cl04+Cl05)/4    DLK1
## 3            5044         Cl01 Cl01-(Cl02+Cl03+Cl04+Cl05)/4  RPS4Y1
## 4            2291         Cl01 Cl01-(Cl02+Cl03+Cl04+Cl05)/4    GPC3
## 5            4285         Cl01 Cl01-(Cl02+Cl03+Cl04+Cl05)/4  PLXNA2
## 6            1640         Cl01 Cl01-(Cl02+Cl03+Cl04+Cl05)/4   EFNA5
##       logFC   logCPM        LR      P.Value    adj.P.Val
## 1 -13.99068 8.751824 115.39303 6.454855e-27 2.281468e-22
## 2  14.75266 8.804668  95.24763 1.679989e-22 2.968961e-18
## 3  13.36825 7.424298  66.29970 3.873178e-16 4.563249e-12
## 4  13.06897 7.126492  61.43385 4.578701e-15 4.045854e-11
## 5 -11.22931 8.736883  60.23644 8.412054e-15 5.946481e-11
## 6  13.83358 8.430487  55.30939 1.029755e-13 6.066113e-10
```

Notice that now there is both a “Contrast” and a “ContrastName” column, unlike with the pairs comparison. Like before, “Contrast” gives an explicit definition of what is the comparisons, in the form of “(Cl02+Cl03+Cl04+Cl05+Cl06)/5-Cl01”, meaning the mean of the means of clusters 2-6 is compared to the mean of cluster1. Note that the contrasts here are always written in terms of the internal (numeric) cluster id, with an “Cl” in front of the number and a ‘0’ to make the number 2 digits. “ContrastName” interprets this into a more usable name, namely that this contrast can be easily identified as a test of “Cl01” (cluster 1).

We can plot the contrasts with a heatmap for these results. Here we notice that the color next to the gene group matches the cluster that the contrast matches.

```
plotContrastHeatmap(rsecFluidigm,signifTable=best1vsAll,nBlankLines=10, whichCluster="primary")
```

### 7.1.3 Dendrogram

The option `type="Dendro"` is more complex; it assumes that there is a hierarchy of the clusters (created by `makeDendrogram` and stored in the `ClusterExperiment` object). Then for each *node* of the dendrogram, `getBestFeatures` defines a contrast or comparison of the mean expression between the daughter nodes.

```
bestDendro<-getBestFeatures(rsecFluidigm,contrastType="Dendro",DEMethod="edgeR",p.value=0.05,number=NROW(rsecFluidigm))
head(bestDendro)
```

```
##   IndexInOriginal ContrastName               Contrast Feature      logFC
## 1            1284        Node1 (X4+X1+X3)/3-(X5+X2)/2   CXADR  -7.193510
## 2            5211        Node1 (X4+X1+X3)/3-(X5+X2)/2  SEMA3C -10.818739
## 3            2325        Node1 (X4+X1+X3)/3-(X5+X2)/2   GRIA2  -9.884674
## 4            4285        Node1 (X4+X1+X3)/3-(X5+X2)/2  PLXNA2  -8.490279
## 5            3724        Node1 (X4+X1+X3)/3-(X5+X2)/2 NEUROD6 -10.873128
## 6            5333        Node1 (X4+X1+X3)/3-(X5+X2)/2     SLA  -9.636881
##      logCPM       LR      P.Value    adj.P.Val
## 1 10.907220 44.56590 2.459383e-11 1.159025e-07
## 2  8.749876 43.15285 5.062611e-11 1.789380e-07
## 3  8.238138 38.89834 4.464602e-10 1.238610e-06
## 4  8.736883 38.74945 4.818470e-10 1.238610e-06
## 5  7.752301 37.92304 7.359106e-10 1.669422e-06
## 6  8.897872 37.71454 8.189169e-10 1.669422e-06
```

Again, there is both a “ContrastName” and “Contrast” column. The “Contrast” column identifies which clusters ids were on each side of the node (and hence commpared) and “ContrastName” is the name of the node, determined internally during `makeDendrogram`.

```
levels((bestDendro)$Contrast)
```

```
## [1] "(X4+X1+X3)/3-(X5+X2)/2" "X4-(X1+X3)/2"          
## [3] "X5-X2"                  "X1-X3"
```

We can look at the results again with `plotContrastHeatmap`.

```
plotContrastHeatmap(rsecFluidigm,signifTable=bestDendro,nBlankLines=10)
```

We can plot the dendrogram to help make sense of which contrasts go with which nodes and choose to show the node names with `show.node.label=TRUE` (plotDendrogram calls `plot.phylo` from the `ape` package and can take as imput those arguments like `show.node.label`).

```
plotDendrogram(rsecFluidigm,show.node.label=TRUE,whichClusters=c("makeConsensus","mergeClusters"),leaf="samples",plotType="colorblock")
```

## 7.2 DE Analysis for count and other RNASeq data

The `getBestFeatures` method for `ClusterExperiment` objects has an argument `DEMethod` to determine what kind of DE method should be run. The options are `limma`, `limma-voom` and `edgeR`. The last two options assume that `assay(x)` are counts.

- **`limma`** In this case, the data is assumed to be continous and roughly normal and the `limma` DE method is performed on `transformData(x)`, i.e. the data after transformation of the data with the transformation stored in the `ClusterExperiment` object.
- **`limma-voom`** refers to calling limma with the `voom`(Law et al. 2014) correction which uses the normal linear model of `limma` but deals with the mean-variance relationship that is found with count data. This means that the differential expression analysis is done on \(log\_2(x+0.5)\). This is *regardless of what transformation is stored in the `ClusterExperiment` object*! The `voom` call within `getBestFeatures` sets `normalize.method = "none"` in the call to `voom`. Unlike edgeR or DESeq, the voom correction does not explicitly require a count matrix, and therefore it has been proposed that it can be used on FPKM or TPM entries, or data normalized via RUV. However, the authors of the package do not recommend using voom on anything other than counts, see e.g. this discussion.
- **`edgeR`** Performs a likelihood-ratio test (`glmLRT` function in `edgeR` (Robinson, Mccarthy, and Smyth 2010)) based on a negative binomial distribution and a empirical bayes estimation of the dispersion.
- **`edgeR` with zero-inflated weights** Weights can be provided to `getBestFeatures`, in which case the LRT is performed using the function `glmWeightedF` in the `zinbwave` package following the work of (Van den Berge et al. 2018). The best way to do this is to save the weights in an assay entitled `weights`. Here is an example of mock code:

```
assay(rsecFluidigm,"weights")<- myweights
```

If the initial `SummarizedExperiment` object is set up this way, then the functions `getBestFeatures`, `mergeClusters` , and `RSEC` will all automatically look for such an assay in order to use these weights, if `DEMethod="edgeR"`. Indeed for `RSEC` this is the *only* way to get weights used – unlike `getBestFeatures` and `mergeClusters`, `RSEC` doesn’t give a way for the user to make choices about the weights (this is to simplify the number of parameters). For `getBestFeatures` and `mergeClusters` you can manually set the argument `weights` to be `NULL` to force the functions to NOT use the weights stored here (and the standard edgeR routine is followed instead) or alternatively set `weights` to be another assay name or even a matrix of weights.

**Normalization Factors** Both `limma-voom` and `edgeR` calls first set up a `DGEList` object which is then given to either the `voom` function in the `limma` package or the `estimateDisp` function of `edgeR`. The argument `counts` to the `DGEList` function is given by the data in the assay, but you can pass other arguments to the call to `DGEList` by giving them in a list format to the argument `dgeArgs` of `getBestFeatures` (or the other functions that call `getBestFeatures`). In particular, you can pass arguments such as `norm.factors` or `lib.size` to specify normalization factors, which will then be used by the `voom`/`edgeR` commands.

## 7.3 Piping into other DE routines

Ultimately, for many settings, the user may prefer to use other techniques for differential expression analysis or have more control over certain aspects of it. The function `clusterContrasts` may be called by the user to get the contrasts that are defined within `getBestFeatures` (e.g. dendrogram contrasts or pairwise contrasts). These contrasts, which are in the format needed for `limma` or `edgeR` can be piped into programs that allow for contrasts in their linear models for mRNA-Seq; they can also be chosen to be returned in the formated needed by MAST (Finak et al. 2015) for single-cell sequencing by settting `outputType="MAST"`.

Similarly, more complicated normalizations, like RUV (Gagnon-Bartsch and Speed 2011), adjust each gene individually for unwanted batch or other variation within the linear model. In this case, a matrix \(W\) that describes this variation should be included in the linear model. Again, this can be done in other programs, using the contrasts provided by `clusterContrasts` in combination with \(W\).

## 7.4 Multiple Testing adjustments

The user should be careful about questions of multiple comparisons when so many contrasts are being performed on each feature; the default is to correct across all of these tests (see the help of `getBestFeatures` and the argument `contrastAdj` for more). As noted in the introduction, p-values created in this way are reusing the data (since the data was also used for creating the clusters) and hence should not be considered valid p-values regardless.

As mentioned, `getBestFeatures` accepts arguments to `limma`’s function `topTable` (or `topTags` for `edgeR`) to decide which genes should be returned (and in what order). In particular, we can set an adjusted p-value cutoff for each contrast, and set `number` to control the number of genes returned *for each contrast*. By setting `number` to be the length of all genes, and `p.value=0.05`, we can return all genes for each contrast that have adjusted p-values less than 0.05. All of the arguments to `topTable` regarding what results are returned and in what order can be given by the user at the call to `getBestFeatures`.

# 8 Working with other `assays()`

By default, `RSEC` and all the other user-facing functions in `clusterExperiment` will use the first `assay` if applied to `SummarizedExperiment`-like objects (including `SingleCellExperiment` and `ClusterExperiment`).

However, it is often the case that one needs to use the data saved in a different `assay` slot for clustering, or even two different `assays` for clustering and visualization. `clusterExperiment` allows for this level of flexibility, by using the `whichAssay` option in all the relevant functions.

As an example, consider the `fluidigm` dataset: it is saved as a `SummarizedExperiment` with `assay` several slots.

```
assayNames(fluidigm)
```

```
## [1] "tophat_counts"  "cufflinks_fpkm" "rsem_counts"    "rsem_tpm"
```

Assume that we want to use the RSEM TPM values to cluster the data with RSEM.

```
se2 <- fluidigm[,colData(fluidigm)[,"Coverage_Type"]=="High"]
wh_zero <- which(rowSums(assay(se2, "rsem_tpm"))==0)
pass_filter <- apply(assay(se2, "rsem_tpm"), 1, function(x) length(x[x >= 10]) >= 10)
se2 <- se2[pass_filter,]
dim(se2)

rsecTPM <- RSEC(se2, whichAssay = "rsem_tpm", transFun = log1p, mergeDEMethod="limma",
              reduceMethod="PCA", nReducedDims=10, consensusMinSize=3, 
              ncores=1, random.seed=176201)
```

Note that in this case, the merging is done after determining DE with `limma` rather than `edgeR` since we no longer have count values.

**Switching between assays and transformations** If you plan to use different assays for different functions, be careful about the built-in transformation function that is saved in `ClusterExperiment` objects – and which will be applied to any assay! The data will be silently transformed by pretty much every function in the package before any calculations are performed. You can set the transformation function with the command `transformation(x)<-...`, including setting it to the identify `function(x){x}`. If you plan on using a dimensionality reduction like PCA, then if they are already saved in the object (using `reducedDims`) then the functions in the `RSEC` workflow will use those and not need to transform the data. But setting `isCount=TRUE` in `RSEC` will set the transformation of the object (and also by default sets the `DEMethod` for `mergeClusters` unless the user over-rides it) even if RSEC uses the saved dimensionality reductions and the data is never transformed for the clustering analysis.

# 9 Session Information

This vignette was compiled under:

```
sessionInfo()
```

```
## R version 3.5.0 (2018-04-23)
## Platform: x86_64-apple-darwin15.6.0 (64-bit)
## Running under: OS X El Capitan 10.11.6
## 
## Matrix products: default
## BLAS: /Library/Frameworks/R.framework/Versions/3.5/Resources/lib/libRblas.0.dylib
## LAPACK: /Library/Frameworks/R.framework/Versions/3.5/Resources/lib/libRlapack.dylib
## 
## locale:
## [1] C/en_US.UTF-8/en_US.UTF-8/C/en_US.UTF-8/en_US.UTF-8
## 
## attached base packages:
## [1] parallel  stats4    stats     graphics  grDevices utils     datasets 
## [8] methods   base     
## 
## other attached packages:
##  [1] igraph_1.2.1                scran_1.8.1                
##  [3] clusterExperiment_2.1.5     SingleCellExperiment_1.2.0 
##  [5] scRNAseq_1.6.0              SummarizedExperiment_1.10.1
##  [7] DelayedArray_0.6.0          BiocParallel_1.14.1        
##  [9] matrixStats_0.53.1          Biobase_2.40.0             
## [11] GenomicRanges_1.32.2        GenomeInfoDb_1.16.0        
## [13] IRanges_2.14.8              S4Vectors_0.18.1           
## [15] BiocGenerics_0.26.0         BiocStyle_2.8.0            
## 
## loaded via a namespace (and not attached):
##   [1] uuid_0.1-2               backports_1.1.2         
##   [3] copula_0.999-18          NMF_0.21.0              
##   [5] plyr_1.8.4               lazyeval_0.2.1          
##   [7] shinydashboard_0.7.0     splines_3.5.0           
##   [9] pspline_1.0-18           rncl_0.8.2              
##  [11] ggplot2_2.2.1            gridBase_0.4-7          
##  [13] scater_1.8.0             digest_0.6.15           
##  [15] foreach_1.4.4            htmltools_0.3.6         
##  [17] viridis_0.5.1            magrittr_1.5            
##  [19] memoise_1.1.0            cluster_2.0.7-1         
##  [21] doParallel_1.0.11        limma_3.36.1            
##  [23] annotate_1.58.0          stabledist_0.7-1        
##  [25] prettyunits_1.0.2        colorspace_1.3-2        
##  [27] blob_1.1.1               xfun_0.1                
##  [29] dplyr_0.7.4              tximport_1.8.0          
##  [31] RCurl_1.95-4.10          genefilter_1.62.0       
##  [33] bindr_0.1.1              phylobase_0.8.4         
##  [35] survival_2.42-3          iterators_1.0.9         
##  [37] ape_5.1                  glue_1.2.0              
##  [39] registry_0.5             gtable_0.2.0            
##  [41] zlibbioc_1.26.0          XVector_0.20.0          
##  [43] kernlab_0.9-26           Rhdf5lib_1.2.0          
##  [45] prabclus_2.2-6           DEoptimR_1.0-8          
##  [47] HDF5Array_1.8.0          abind_1.4-5             
##  [49] scales_0.5.0             mvtnorm_1.0-7           
##  [51] DBI_1.0.0                edgeR_3.22.1            
##  [53] rngtools_1.2.4           Rcpp_0.12.16            
##  [55] viridisLite_0.3.0        xtable_1.8-2            
##  [57] progress_1.1.2           bit_1.1-12              
##  [59] mclust_5.4               DT_0.4                  
##  [61] glmnet_2.0-16            htmlwidgets_1.2         
##  [63] httr_1.3.1               FNN_1.1                 
##  [65] RColorBrewer_1.1-2       fpc_2.1-11              
##  [67] modeltools_0.2-21        pkgconfig_2.0.1         
##  [69] XML_3.98-1.11            flexmix_2.3-14          
##  [71] nnet_7.3-12              locfit_1.5-9.1          
##  [73] dynamicTreeCut_1.63-1    later_0.7.2             
##  [75] howmany_0.3-1            rlang_0.2.0             
##  [77] softImpute_1.4           reshape2_1.4.3          
##  [79] AnnotationDbi_1.42.1     munsell_0.4.3           
##  [81] tools_3.5.0              RSQLite_2.1.1           
##  [83] ade4_1.7-11              evaluate_0.10.1         
##  [85] stringr_1.3.1            yaml_2.1.19             
##  [87] knitr_1.20               bit64_0.9-7             
##  [89] robustbase_0.93-0        purrr_0.2.4             
##  [91] dendextend_1.8.0         bindrcpp_0.2.2          
##  [93] nlme_3.1-137             mime_0.5                
##  [95] whisker_0.3-2            xml2_1.2.0              
##  [97] compiler_3.5.0           beeswarm_0.2.3          
##  [99] statmod_1.4.30           tibble_1.4.2            
## [101] RNeXML_2.1.1             pcaPP_1.9-73            
## [103] stringi_1.2.2            gsl_1.9-10.3            
## [105] RSpectra_0.12-0          lattice_0.20-35         
## [107] trimcluster_0.1-2        Matrix_1.2-14           
## [109] pillar_1.2.2             ADGofTest_0.3           
## [111] zinbwave_1.2.0           data.table_1.11.2       
## [113] bitops_1.0-6             httpuv_1.4.3            
## [115] R6_2.2.2                 promises_1.0.1          
## [117] bookdown_0.7             gridExtra_2.3           
## [119] vipor_0.4.5              codetools_0.2-15        
## [121] MASS_7.3-50              assertthat_0.2.0        
## [123] rhdf5_2.24.0             MAST_1.6.1              
## [125] rjson_0.2.18             pkgmaker_0.22           
## [127] rprojroot_1.3-2          GenomeInfoDbData_1.1.0  
## [129] locfdr_1.1-8             diptest_0.75-7          
## [131] grid_3.5.0               tidyr_0.8.0             
## [133] class_7.3-14             rmarkdown_1.9           
## [135] DelayedMatrixStats_1.2.0 shiny_1.0.5             
## [137] numDeriv_2016.8-1        ggbeeswarm_0.6.0
```

# References

Finak, Greg, Andrew McDavid, Masanao Yajima, Jingyuan Deng, Vivian Gersuk, Alex K Shalek, Chloe K Slichter, et al. 2015. “MAST: a flexible statistical framework for assessing transcriptional changes and characterizing heterogeneity in single-cell RNA sequencing data.” *Genome Biology*, December, 1–13.

Gagnon-Bartsch, Johann A., and Terence P Speed. 2011. “Using control genes to correct for unwanted variation in microarray data.” *Biostatistics (Oxford, England)*, November.

Law, Charity W, Yunshun Chen, Wei Shi, and Gordon K Smyth. 2014. “voom: Precision weights unlock linear model analysis tools for RNA-seq read counts.” *Genome Biology* 15 (2): 1–17.

Ritchie, Matthew E, Belinda Phipson, Di Wu, Yifang Hu, Charity W Law, Wei Shi, and Gordon K Smyth. 2015. “limma powers differential expression analyses for RNA-sequencing and microarray studies.” *Nucleic Acids Research* 43 (7): e47–e47.

Robinson, Mark D, Davis J Mccarthy, and Gordon K Smyth. 2010. “edgeR: a Bioconductor package for differential expression analysis of digital gene expression data.” *Bioinformatics (Oxford, England)* 26 (1): 139–40.

Smyth, Gordon K. 2004. “Linear models and empirical bayes methods for assessing differential expression in microarray experiments.” *Statistical Applications in Genetics and Molecular Biology* 3 (1): Article3–25.

Tseng, George C., and Wing H. Wong. 2005. “Tight Clustering: A Resampling-Based Approach for Identifying Stable and Tight Patterns in Data.” *Biometrics* 61 (1): 10–16.

Van den Berge, Koen, Fanny Perraudeau, Charlotte Soneson, Michael I Love, Davide Risso, Jean-Philippe Vert, Mark D Robinson, Sandrine Dudoit, and Lieven Clement. 2018. “Observation Weights to Unlock Bulk Rna-Seq Tools for Zero Inflation and Single-Cell Applications.” *Genome Biology* 19 (24).
