# Supplementary material for: Correction: clusterExperiment and RSEC: A Bioconductor package and framework for clustering of single-cell and other large gene expression datasets
Source: PLoS Comput Biol. 2019 Jan 11;15(1):e1006727. doi: 10.1371/journal.pcbi.1006727 (PMC6329488; doi:10.1371/journal.pcbi.1006727)
Supplement: S1 Text — Supplemental text giving a more detailed description of the RSEC framework and its implementation, as well as information about the analysis of the datasets. (PDF) [file pcbi.1006727.s001.pdf]

We provide here detailed information regarding the implementation of the procedures described in the main text. For the most current information about implementation issues and function arguments, please consult the help pages in the Bioconductor R package `clusterExperiment`. The accompanying vignette provides extended examples.

1 Infrastructure of the `clusterExperiment` Package

The `clusterExperiment` package supplies a new Bioconductor class, `ClusterExperiment`, for easy storage and manipulation of multiple clustering results, as well as functions for visualization of these clusterings along with the original data. The class inherits from the existing `SingleCellExperiment` class, a baseline class for storing single-cell and other RNA-Seq datasets in Bioconductor [1]. The `ClusterExperiment` class additionally stores how the data should be transformed and whether the input data are counts, so as to appropriately handle data from single-cell RNA-Seq and other transcriptomics experiments. The class provides a formal mechanism for storing clustering results, even when some observations are left unclustered in some of the clusterings, making comparison across clusterings based on different filtering of samples easy.

The `clusterExperiment` package also provides a class, `ClusterFunction`, for storing clustering functions, so that user-defined functions can be easily integrated into the RSEC workflow.

2 `clusterMany`

In what follows, we will use the term *argument* to refer to the user-given arguments to the function `clusterMany` and *parameter* to refer to the values that `clusterMany` will actually pass internally to a clustering routine. A parameter can also be a logical value to include whether or not to perform a certain action (e.g. subsampling).

The function `clusterMany` divides possible parameters into those that can be compared – i.e., those parameters for which `clusterMany` may vary in different runs of clustering algorithms – and those that are fixed to be the same for all of the clusterings runs. We call one set of parameters that will be given to a particular run of a clustering algorithm a *parameter combination*. `clusterMany` will run a clustering algorithm for every parameter combination, resulting in a different clustering result.

The following arguments to the function `clusterMany` allow the user to define the values for the parameters that are allowed to vary within `clusterMany`. Specifically, all of these arguments allow for multiple input values. All combinations across all the values given to these arguments are created, restricted only by limiting to those parameter values that are feasible in conjunction. Each of these combinations will create a parameter combination. Thus, for any the following arguments, a set of values can be given (hence our use of "and/or" in describing the possible values), and all of those values will be used in combination with all of the values of the other arguments. However, only a single value from any argument to `clusterMany` is used in any particular parameter combination (i.e. multiple values given to an argument of `clusterMany` can not be used as a parameter within a single clustering routine). Furthermore, in any particular parameter combination, the individual values of the arguments listed here can potentially be interpreted differently depending on the value of other arguments used for that parameter combination.

Recall in what follows that `clusterExperiment` does not assume that all samples will be assigned to a cluster, depending on the clustering algorithm, and has a special encoding of "-1" for such lack of assignment.

- 'sequential': This argument takes on logical values (i.e. TRUE and/or FALSE), indicating whether the sequential strategy should be implemented or not (see Section 2.3).

- ‘subsample’: This argument takes on logical values (i.e. TRUE and/or FALSE), indicating whether the subsampling strategy for determining a dissimilarity matrix D should be implemented or not.

- ‘clusterFunction’: The clustering functions to be tried in the *main clustering step*. For parameter combinations that include ‘subsample=TRUE’, then the value pulled from the ‘clusterFunction’ argument defines the clustering method that will be used on the matrix D created from subsampling the data. For combinations of arguments where ‘subsample=FALSE’, the value pulled from the ‘clusterFunction’ argument is the clustering method that will be used directly on the original data. Currently the following built-in clustering functions are implemented:
  - k-means, implemented with the function `kmeans` from the package `stats`;
  - PAM, implemented with the function `pam` from the package `cluster`;
  - Clara, implemented with the function `clara` from the package `cluster`;
  - Spectral clustering, implemented with the function `speccin` from the package `kernlab`;
  - Hierarchical clustering with K clusters, implemented with the function `hclust` followed by the function `cuttree` in the package `stats`;
  - Hierarchical clustering with clusters determined by a similarity parameter α, implemented with the function `hclust` followed by our procedure, documented below (“hierarchical01”);
  - Tight clustering, implemented with our code adapted from the package `tight` (see documentation below).

- ‘ks’: For any particular parameter combination, the value pulled from the ‘ks’ argument is interpreted differently depending on the choices of the other arguments included in the parameter combination. If ‘sequential=TRUE’, ‘ks’ defines the parameter ‘k0’ of sequential clustering, which is approximately like the initial starting point for the number of clusters in the sequential process. Otherwise, ‘ks’ is passed to set the parameter ‘k’ of the main clustering step (and by default that of the subsampling step), and is only relevant if the clustering algorithm used from the ‘clusterFunction’ argument in the parameter combination is of a type that requires the user to define a value K.

- ‘reduceMethod’: This argument determines the dimensionality reduction procedure(s) to be used, in which case the clustering will be done on a reduced dataset, rather than the full dataset. `clusterExperiment` provides some built-in methods for dimensionality reduction: “PCA”, “var”, “abscv”, “mad”, “mean”, “iqr”, “median” and/or “none”. However, the user can also provide their own set of dimensionality reductions, described more fully in Section 2.1 below. “var”,“abscv”, “mad”,“mean”,“iqr”, “median” refer to filtering the data to a subset of existing genes, and indicate the method of selecting that subset of genes. The options are to chose the genes with the largest variance, absolute coefficient of variation (CV), median absolute deviation (MAD), mean, inter-quartile range (IQR) and/or coefficient of variation (CV), respectively. “PCA” refers to clustering on the top principal components of the genes.

- ‘nFilterDims’ and ‘nReducedDims‘: Based on the values given to “reduceMethod”, the user can set the number of dimensions to use via the arguments “nFilterDims” and “nReducedDims”. “nFilterDims” determines the number of genes to select methods that reduce to a subset of the existing genes, while “nReducedDims” determines the number of the new features to select for methods that use new features created from the original genes. Like the other arguments, these arguments can be given a range of values, meaning all combinations of these values will be tried. However, in creating parameter combinations, the different types of dimensionality reduction are never mixed (e.g., “PCA” and “mad” are never in the same parameter combination), and therefore the values of “nFilterDims” and

"nReducedDims" are also never mixed together despite being separate arguments to `combineMany`– each parameter combination that will be passed to the clustering algorithm either has a value from from "nFilterDims" OR from "nReducedDims".

- 'distFunction': This argument determines the distance function to be used in clustering (default being Euclidean), only applicable when 'reduceMethod' does not imply a dimensionality reduction that is not a new set of features, like "PCA" (see Section 2.1 – it is assumed that for such newly defined features, the Euclidean distance is the correct distances)
- 'minSizes': The minimum size required for a cluster – samples in clusters smaller than this size are reclassified as "unassigned" (-1).
- 'alphas': The values for $\alpha \in (0, 1)$ parameters for clustering techniques that determine clusters based on the required amount of similarity rather than the number of clusters K. Larger values of α are less stringent on the amount of similarity required in a cluster (like significance levels α in hypothesis testing).
- 'betas': This argument is used when "sequential=TRUE" and the values of this argument are passed to the β parameter used by sequential clustering (see below, Section 2.3) to determine the level of stability required between to determine that a stable cluster has been found. Larger values of β require greater stability between clusters.
- 'findBestK': This argument takes on logical values and is only applicable for parameter combinations where the clustering algorithms has a parameter "k" that defines the number of clusters K. In parameter combinations where "findBestK=TRUE", then K will be chosen automatically by running a range of K values and choosing clustering from the K that gives the largest average silhouette width.
- 'removeSil': This argument takes on logical values and determines whether samples with small silhouette width are removed from their cluster assignment, in the sense that they are not given their original cluster assignment but instead "unassigned" (-1).
- 'silCutoff': This argument takes on numerical values and is only applicable for parameter combinations where 'removeSil' is TRUE. In this case, the values of 'silCutoff' determines the silhouette width cutoff used for determining that the sample will be removed from their clustering assignment and instead classified as unassigned. This value defaults to 0, but can take on a discrete set of values, in which all of these values will be attempted.

Most of the clustering methods and procedures available in the package are straightforward applications of general, existing techniques. We document here only a few of the clustering methods that we have extensively adapted.

"hierarchical01": Hierarchical clustering based on within-cluster similarity We provide a clustering method based on the level of similarity between samples within each cluster and call this method "hierarchical01" (to distinguish it from hierarchical clustering with a prespecified number of clusters K obtained via `cuttree`). Specifically, we first run hierarchical clustering on the data, using the standard `hclust` function and an input dissimilarity matrix D. The matrix D, however, is required to take on values between 0 and 1. Starting at the root, we go down the hierarchical clustering dendrogram, checking for each node $\mathcal{N}$ whether it satisfies one of two possible criteria for determining whether the samples that comprise it are sufficiently similar:

1. all pairwise distances are $< \alpha$ (method "maximum");

2. for each sample i, $m_i < \alpha$, where m_i is the mean of the pairwise distances of sample i to the other samples in the cluster (method "average").

The default option is "maximum". Samples that do not satisfy this criterion for any node are not assigned to a cluster.

"tight": Adaptation of the code of [2] The tight clustering algorithm of [2] included subsampling of the data, a method for clustering of the resulting dissimilarity matrix D, and a procedure for repeating this process sequentially. We have modularized these steps.

We have modularized the method for clustering a dissimilarity matrix D that takes on values in $(0, 1)$ into a possible clustering method for the user to pick ("tight"). This method also requires not determination of the number of clusters K, but a level of allowed dissimilarity α. The code is pulled from the package `tight` with minimal adaptation applied to the clustering procedure. It was written with the intention of being applied to a D that was the result of subsampling. The algorithm first finds core samples, namely those that have dissimilarity 0, and picks the largest such group (where the largest group may be of size one). It then adds samples to that core group if samples have dissimilarity with all of the core group members that is no greater than α, and repeats this process until no remaining samples satisfy the criteria. Samples that do not satisfy this criteria are not assigned to a cluster.

2.1 Dimensionality Reduction

There are two varieties of dimensionality reduction supported in 'clusterExperiment' package via that "reduceMethod" argument.

1. creating a reduced data set by subsetting the original dataset to a smaller subset of genes, which is done by calculating statistic for each gene, and filtering to only genes with high (or low) values of this statistic.

2. creation of a smaller number of new features that are functions of the original genes, i.e. *not* a simple selection of genes, but a new set of variables to represent the data (some times referred to as "metagenes")

For simplicity, we'll refer to the first as filtering of the data and second as a dimensionality reduction. This is because in the first case, the reduced data set can be quickly recreated by subseting the original data, so long as the per-gene statistics have been saved. This means only a single vector of the length of the number of genes needs to be stored for the first type of dimensionality reduction (filtering) while the second kind requires saving a matrix with a value for each observation for each new variable.

The `ClusterExperiment` class created in `clusterExperiment` inherits from the standard Bioconductor `SingleCellExperiment` class. Briefly, the `SingleCellExperiment` class extends the `SummarizedExperiment` class to give a structure for saving the reduced matrices from the second class of dimensionality reductions we described above. This gives a unified way to save the results of applying a dimensionality reduction method of the second type. Multiple such dimensionality reductions can be stored, and the user gives them names, e.g. "PCA" or "tSNE".

The `clusterExperiment` package uses this structure both to save the results of dimensionality reductions if they are calculated by the `clusterMany` function and also to allow the functions of the package to reuse them if they have already been created. In this way, all of the functions in the `clusterExperiment` package can make use of any dimensionality reduction method previously calculated and saved by the user.

The `clusterExperiment` package also provides a similar procedure for storing the filtering statistics (i.e. statistics calculated on each gene that can be used to subset to a reduced set of genes). Therefore, if

the user has already calculated a per-gene statistic and appropriately saved it, this user-defined statistic can be used for filtering instead of those provided by the package.

2.2 Subsampling

The subsampling option in `clusterExperiment` generates clusterings based on randomly sampled subsets of the full set of n observations to be clustered. Each resampled subset of the dataset is clustered, leading to a collection of clusterings which are then summarized by an $n \times n$ co-clustering matrix, with entry p_{ij} defined as the proportion of times the pair of samples i and j were in the same cluster across all of the resampled datasets. `clusterExperiment` offers three different ways in which the calculation of p_{ij} can be performed, which differ based on whether samples not included in the random subset are classified into clusters.

InSample The classification of a sample into clusters is done only if the sample is part of the random subset and the classification they receive is that given by the clustering algorithm.

All After the clustering has been produced on the random sample, *all* samples are then (re)classified into clusters based on a classification method dependent on the clustering function.

OutOfSample After the clustering has been produced on the random sample, only samples *not* included in the random sample are classified into clusters based on a classification method dependent on the clustering function.

The classification method mentioned above to classify an arbitrary sample into a cluster must be defined, and not all algorithms will have such a feature (it is an optional part of defining a 'ClusterFunction' object). If missing, `clusterExperiment` will silently set the option to "InSample" which does not require a separate classification method. Note that because of the randomness in subsampling, the number of times a pair i and j were in the random subset varies for each pair, and thus the denominator of p_{ij} for "InSample" and "OutOfSample" is a random variable differing for each pair (i, j). For example, if m subsamples are taken of size k out of N total cells, then the expected value of the denominator for "InSample" is

$$m\frac{k^2(k-1)^2}{N(N-1)}.$$

The default option is "All", if the clustering algorithm given has a classification function provided, in which case the proportion is always based on all samples. Of the built-in functions provided by `clusterExperiment`, "kmeans","pam", and "clara" all have classification functions provided, which is to classify them to the nearest cluster center.

Subsampling therefore defines a dissimilarity matrix between samples, with entries $D_{ij} = 1 - p_{ij}$, but does not itself define a clustering of the samples. The dissimilarity matrix D is next used by the RSEC workflow to cluster the samples. It is important to note that the clustering algorithm applied to D does not need to be that which was used on the resampled datasets. For example, a matrix D that results from partitioning each of the resampled datasets into K clusters will not necessarily be amenable itself to a partition into K clusters. Our experience has been that because D is the result of averaging over clusterings, it is more robust to the choice of K than the underlying clustering algorithm.

Furthermore, because D is a dissimilarity matrix, with entries on a well-defined scale of 0-1, it is intuitive to constrain the level of between-sample dissimilarity within clusters, rather than setting a particular K for the number of clusters [2]. In this way, the choice of K becomes instead a choice of $\alpha \in (0, 1)$, defining the amount of dissimilarity allowed within a cluster. While this doesn't change the reliance on the selection of a tuning parameter, we find that specifying the dissimilarity α is more natural and also more robust for datasets

with widely differing numbers of actual clusters to detect. The `clusterMany` function, of course, allows the user to easily apply multiple methods for clustering the D matrix, as well as a range of corresponding parameters α or K, for comparison.

Because subsampling results in two layers of clustering algorithms – one applied to the resampled datasets and one applied to the dissimilarity matrix D – this leads to an expansion of choices that can be tried by `clusterMany`. For simplicity, `clusterMany` only allows the key parameters involved in the clustering of the matrix D to be given multiple options, while the parameters for the clustering of the resampled datasets are fixed for all the comparisons that involve subsampling (though the user can set them).

2.3 Sequential clustering

As mentioned previously, the tight algorithm of [2] included both subsampling of the data, a method for clustering of the resulting dissimilarity matrix D, and a procedure for repeating this process sequentially. We use the process they describe there for sequentially finding stable clusters for our sequential method. As noted above, we modularize the components of their algorithm, generalizing the sequential clustering to apply to any clustering technique, and with or without subsampling. Specifically, the "best" cluster is chosen to be that cluster which varies the least in its membership as the parameter controlling the number of clusters K is increased, as measured in the maximal percentage overlap of clusters from clusterings from K and $K+1$ (the ratio of cardinality of intersection to cardinality of union). In the case where the user does not make use of resampling, the parameter K refers directly to the number of clusters for the main clustering algorithm; if instead the user chooses to make use of resampling, the parameter K refers to the number of clusters for the base clustering method run on the subsampled datasets (and does not directly dictate the final number of clusters). When a cluster is found where a proportion of at least β of its members remain in it as the parameter K is increased, then the cluster is identified to be a stable cluster, the samples in it removed from further consideration, and the process begins again to find another stable cluster.

In more detail, under our generalization, the sequential searching for a cluster works in one of two ways. Either it is applied to

1. the results of a clustering algorithm that directly clusters the data, in which case the clustering function must be such that it requires the user to set K, the number of clusters

2. the results of a clustering algorithm that is applied to a dissimilarity function D that is the result of repeated clustering of subsampled data. In this case the clustering algorithm applied to the *subsampled* data must be such that it requires the user to set K, but the algorithm applied to the resulting D matrix is arbitrary; if the clustering matrix applied to D requires the user to set K to get a clustering, it is set to the K used in subsampling.

The sequential starts by setting the value $K = k_0$ (either at the main clustering level or the subsampled clustering level), and continues to increases k_0 until a cluster is found such that the similarity between the cluster with K and $K-1$ is greater than or equal to β. Specifically the method looks at the top M clusters in size from K and $K-1$, and between each pair of two clusters of samples $i(K)$ and $j(K-1)$ that are clusters within the clusterings found with K and $K-1$ clusters, respectively, the following stability measure is calculated:

$$\frac{|i(K) \bigcap j(K-1)|}{|i(K) \bigcup j(K-1)|}$$

The cluster $j(K-1)$ with stability $\geq \beta$ is chosen and if multiple such clusters are found, then the largest such cluster $j(K-1)$ is chosen by default. After finding such a cluster, the samples in this cluster are considered a cluster and removed from further consideration and k_0 is decreased by 1 and the process is continued until no more clusters can satisfy this condition, or there are too few samples remaining.

3 makeConsensus

In order to find the ensemble clustering from the results of clusterMany (or what ever set of clusterings given by the user), makeConsensus first converts all non-assigned samples in a clustering (internally encoded with a -1 or -2) into NA values.

If the user requires all samples to be clustered together 100% of the time, then the makeConsensus simply partitions the samples based on the set of unique cluster identifications across all of the clusterings, including non-assignment to clusters ("-1" values).

Otherwise, makeConsensus calculates the Hamming distance between samples, which is the number of positions at which the corresponding symbols in a string are different, and converts this into a percentage. This calculation is implemented by adapting the code posted by Johann de Jong [3] for finding hamming distance quickly in R, and adapted by us to exclude "-1" (NA) values in counting and in finding the proportion so that the proportion given back is the proportion of times a pair of samples do not cluster together *out of the clusterings for which both samples have a cluster assignment*. Those samples that have no clusterings for which neither are "-1" are given a distance of 1.

This distance matrix D is then provided to our internal clustering wrapper, which applies our "hierarchical01" clustering described above with the method argument set to "average", and α equal to $1 - p$, where p is the user-given value describing the how much shared proportion they want for samples to be considered clustered together. Samples that do not meet that requirement are not given any cluster assignment. Furthermore, any samples with too large of a number of unassigned (-1) values across the samples is then unassigned from their cluster (i.e. given value "-1"), the cutoff for which can be controlled by the user.

4 makeDendrogram

For each cluster, makeDendrogram calculates the median per gene/feature within a cluster. The function then calls the R function hclust on the squared euclidean distance of the median of the clusters. The makeDendrogram function allows users to filter the genes that are used in the calculation, with the default being to use the top 500 genes based on median absolute deviation ("mad").

Also passed to hclust is the number of samples per cluster via the argument members of hclust. According to the documentation of hclust, if this argument is given, the input dissimilarity matrix "is taken to be a dissimilarity matrix between clusters instead of dissimilarities between singletons and members gives the number of observations per cluster. This way the hierarchical cluster algorithm can be started in the middle of the dendrogram, e.g., in order to reconstruct the part of the tree above a cut (see examples)."

5 mergeClusters

mergeClusters takes as input the dendrogram from makeDendrogram and performs for each node $\mathcal{N}$ of the dendrogram a significance test (per gene) of the difference in the mean expression between the samples that are descendants of the two daughter nodes of $\mathcal{N}$. The differential expression is determined by first fitting the full model (i.e. all clusters included) and then a test of the contrast (or difference) between the average of the means of the clusters that are the descendants of one daughter nodes of $\mathcal{N}$ with the average of the means of the clusters that are the descendants of the other daughter node. Note that because the clusters are of different sizes, this is different than simply applying a t-test between the samples descendant from one daughter node against the samples descendant from the other daughter node. This reduces the dominance of large clusters. This is implemented using the getBestFeatures function, and the user can choose the DE method to be used (limma [4], limma with voom weights correction for counts [5], edgeR [6], or edgeR with weights, e.g. to account for zero-inflation [7]).

The resulting full set of p-values are provided as input to one of several different methods for calculating the proportion of non-null hypothesis tests in a set of p-values. The available methods are

- "Storey" refers to the method of [8] where the proportion of null hypothesis is estimated as

$$\frac{\#\text{ pvalues } > \lambda}{(1-\lambda)(\#\text{ pvalues})}$$

  and we set $\lambda = 0.5$.

- "PC" refers to the method of [9] where the proportion null is estimated as twice the average p-value.
- "JC" refers to the method of [10], and the implementation is copied from code available on Jiashin Ji's website [11] as of December 16, 2015.
- "locfdr" refers to the method of [12] and uses the implementation in the package `locfdr`.
- "MB" refers to the method of [13] and uses the implementation in the package `howmany`.
- "adjP" refers to simply calculating the proportion of genes that are found significant based on a FDR adjusted p-values (method "BH" in `p.adjust` in R) and a cutoff of 0.05.

  If the method chosen is "adjP", a further value can be optionally given to the argument "logFCcutoff", indicated that a gene is only considered significant in this calculation if *both* the p-value is less than 0.05 and the estimated log fold-change is greater than the value of "logFCcutoff".

6 The `RSEC` Function

While the main steps of our clustering framework are implemented in separate functions available to the user, we provide a single wrapper function `RSEC` around these individual functions, with parameter choices we find particularly relevant for finding robust, small, homogenous clusters that are often desirable for large or noisy gene expression studies, such as scRNA-Seq. Specifically, the clustering method used in the first step of `RSEC` is the tight clustering strategy of [2] adapted by us for single-cell studies. This clustering method makes use of both the subsampling and sequential detection options of `clusterMany` that make the clustering more robust. Furthermore, it results in small, homogeneous clusters without explicitly requiring the user to define the number of clusters K *a priori*; instead, it requires the user to define the level of similarity α between samples in a cluster that is desired. These parameters are more intuitive and scale better with the large numbers of samples that are seen in single-cell sequencing studies. Furthermore, our experience is that because of the underlying subsampling, variations in these parameters do not result in large changes in clusters, as compared to changing K for clustering methods. During the step of RSEC that varies the parameters of this method, the resulting ensemble clustering resembles a robust combination of perturbed clusterings.

7 Data used in the Manuscript

We used the single-cell RNA-Seq dataset on neuronal stem cell differentiation in the mouse olfactory epithelium (OE) from [14]. The featureCounts estimates of the number of reads per gene for this data is available from GEO with accession number GSE95601. The published clustering results (to which we compared our results) were retrieved from the github repository of [14], `www.github.com/rufletch/p63-HBC-diff`.

We preprocessed the read count data following the procedures of [14], including their normalization and filtering of genes. Specifically, we normalized the data by performing PCA on quality metrics of the samples, and then regressed out from the gene expression the effects due to the first PCA of the QC metrics. We removed all undetected genes (i.e. counts of 0 in all cells), as well as removing the ERCC and the CreER gene (used for FACS sorting of the cells). We filtered cells based on QC-metric using the `metric_sample_filter` function available in the `scone` package [15]. We further removed genes that did not have at least 40 counts in 5 cells. We note that [14] further removed some cells that they identified as contaminant which we did not.

See the methods of [14] for details on their analysis and choice of parameters for `RSEC`.

The user implemented function "NN" consists of building a k-nearest-neighbors graph between the cells using the function `buildSNNGraph` from the `scran` [16] package. We then find the densely connected subgraphs using the package `cluster_walktrap` in the R package `igraph`[17]. In this case, K corresponds not to the number of clusters, but the number of nearest neighbors to use in building the graph. To find the best K, the best choice of K was chosen between 5, 10, 15, 20, and 25 nearest neighbors. For the other methods, where K was the number of clusterings, the best K was found by ranging K from 4 to 15.

The second dataset we used consisted of 14,437 cells from the hypothalamus of adult mice sequenced using the Drop-Seq technology [18]. We accessed the data via the bioconductor object of the data made publicly available by [19] at:
`https://scrnaseq-public-datasets.s3.amazonaws.com/scater-objects/chen.rds`).

We followed the authors' description of how they prepared the data, normalizing and scaling the data using the 'NormalizeData' and 'ScaleData' functions from the Seurat package, version 2.3.1, before performing PCA via 'RunPCA' on the results. These PCA results were used by RSEC in creating the clusters (not our built-in calculations of the PCAs).

All of the code for running the analyses, including downloading the data, is available in the github repository for this paper: `www.github.com/epurdom/RSECPaper`.

7.1 Comparison of RSEC to clusters of [18]

The authors of [18] report 45 clusters in their data. After finding the clusters, they classify these clusters into various categories based on the overall gene expression of the cells in these clusters in order to understand their biological function. They classify 34 of these clusters as neuronal, based on *Snap25* and *Syt1* expression, which are further broken down into "Glu" (15 clusters) and "GABA" (18 clusters), and a remaining "Hista" cluster. The remaining 11 non-neuronal clusters are characterized in the original paper based on high expression of the following markers: *Olig1* (oligodendrocytes, 4 clusters), *Cldn5* (endothelial cells, 2 clusters), *C1qa* (2 clusters), and *Sox9* (3 clusters). We note that the labels provided with the public data do not exactly match those in the paper. Specifically, Endo1/Endo2 of the paper appears to correspond to the clusters named Epith1/Epith2 in the publicly available data. Similarly, there is no "NFO" (newly formed oligodendrocyte) cluster as described in the paper, but based on the *Fyn* marker that the paper's supplementary results associate to the cluster, we have identified it as the "IMO" cluster label of the public data. The public data also contains a 46th cluster "SCO", which we are not able to match to any cluster described in the paper.

S4 Fig(b) compares RSEC to the eight large categories of [18] defined by the markers above, while S4 Fig(a) shows the comparisons to the full set of 46 clusters labels provided in the public data.

We now consider further groups that have some small, but noticable differences between RSEC and the clustering of [18]: 1) the division of neuronal cells into inhibitory and excitatory neurons and 2) The division of epithelial cells.

Inhibitory and Excitatory Neurons The authors of [18] further classify their neuronal clusters into inhibitory and excitatory neurons, ("Glu" and "GABA" in [18]) based on expression of the marker genes *Slc17a6* and *Slc32a1*, respectively. We see that RSEC conserves this split (S4 Fig(c)), except for a slight mixing in one of the clusters found by RSEC (*m30*). However, on closer examination of the cells in *m30*, essentially none of the cells in this cluster expresses the excitatory gene marker *Slc17a6*, including those identified by [18] as excitatory (35 cells), while the vast majority of cells in this cluster show strong expression of inhibitory markers (Fig S1). This seems to confirm that this is a cluster of inhibitory cells, not a mixture of the two.

Endothelial Clusters Two clusters of [18] were classified by the authors as Endothelial based on the expression of *Cldn5*. RSEC similarly contains a set of clusters that cleanly separate out the Endothelial cells of [18] from the rest and and show expression of *Cldn5* (Fig S2). However, the RSEC clusters do not match the further division of the endothelial cells into "Endo1" and "Endo2" clusters given by [18]. Instead, RSEC finds six clusters containing the Endothelial cells of [18], which like the neuronal clusters contain a number of cells not classified into any cluster in [18] (Supplementary Figure S2a). Considering only those cells assigned a cluster in [18], some of these clusters are largely subdivisions of the "Endo1" or "Endo2" clusters of [18] (*m6*, *m13*, and *m32*), while some have more substantial mixing, with *m8* being roughly split between the "Endo1" or "Endo2" clusters of [18] (Supplementary Table S1). The authors characterized "Endo1" and "Endo2" biologically by their expression of the genes *Slc38a5* and *Myh11* (in the smaller group of 3,319 cells). We evaluated these expression of these three Epithelial genes markers on the full set of cells, and see that the expression of the *Slc38a5* and *Myh11* markers is not uniform even in "Endo1" and "Endo2" clusters defined by [18]. In fact in both of their clusters, the *median* expression value of the corresponding marker of "Endo1" and "Endo2" is zero, though the upper-quartile of the cluster distinguishes the two clusters (Supplementary Figures S2c,d)). The limited expression of these markers even in the original clustering of [18] makes it difficult to precisely evaluate the clustering of RSEC. However, the RSEC clusters seem to divide the endothelial cells between these three marker genes somewhat more carefully (Supplementary Table S1). In particular, the RSEC clusters separate out those cells showing no expression of the original *Cldn5* marker of [18] for Endothelial cells (clusters *m13* and *m16*). Similarly, those clusters that primarily contain "Endo2" cells (clusters *m13*, *m16*, and *m32*), are divided by RSEC into those that express *Myh11* (*m16* and *m32*) and those that don't (*m3*), unlike the "Endo2" cluster which had the median expression of *Myh11* at zero (see Supplementary Table S1 and Supplementary Figure S2). Combined with *Cldn5*, this separates these three clusters more precisely than the original clusters of [18].

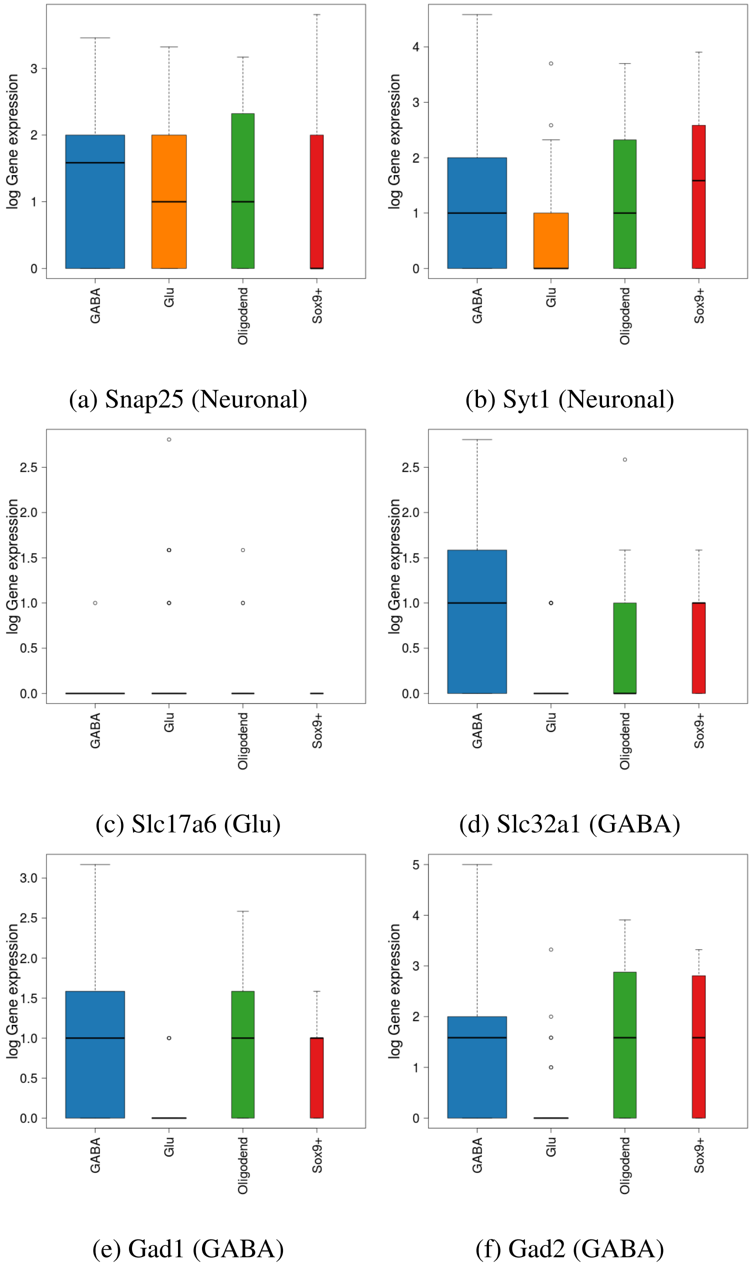

(a) Snap25 (Neuronal)

(b) Syt1 (Neuronal)

(c) Slc17a6 (Glu)

(d) Slc32a1 (GABA)

(e) Gad1 (GABA)

(f) Gad2 (GABA)

Figure S1: **Boxplots of the (log) gene expression values for neuronal markers in cluster m30 using `plotFeatureBoxplot`**. The cells are divided according to their original classification by [18]: GABA (102), Glu (35), Oligodendrocyte (15), Sox9+ (5), Endothelial (1), Hista (1) and Unassigned (418). Expression of either Snap25 or Syt1 define neuronal clusters in [18], while Slc17a6 and Slc32a1 are used by [18] to subdivide them into Glu and GABA clusters, respectively. Gad1 and Gad2 are two other markers of GABA neurons [20].

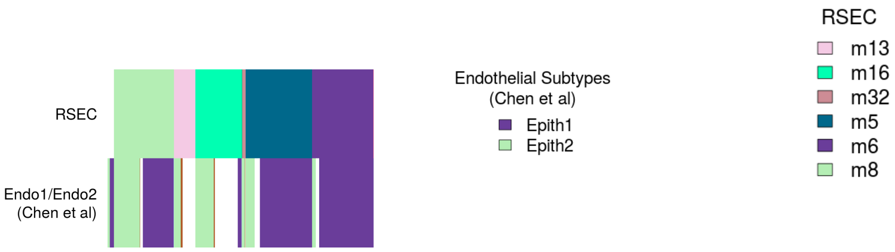

(a) Comparison of endothelial samples, RSEC and [18]

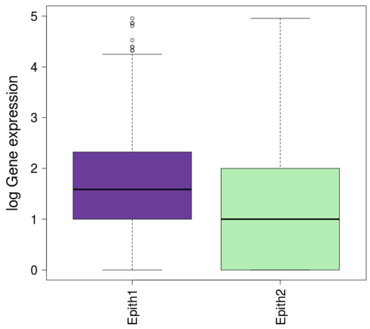

(b) Cldn5 (clusters of [18])

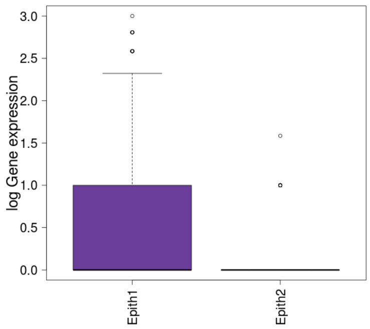

(c) Slc38a5 (clusters of [18])

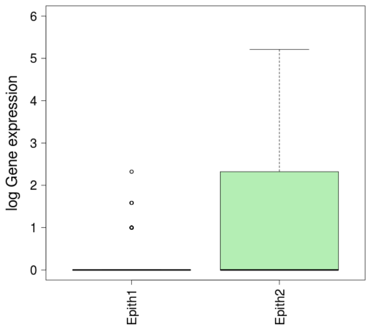

(d) Myh11 (clusters of [18])

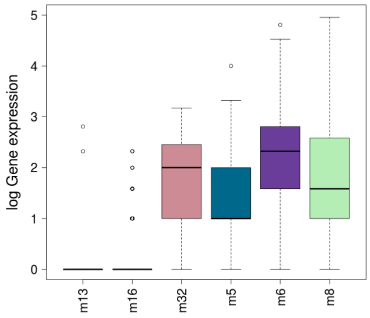

(e) Cldn5 (RSEC)

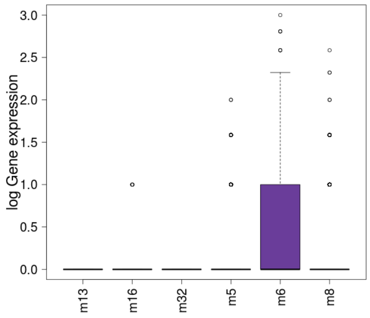

(f) Slc38a5 (RSEC)

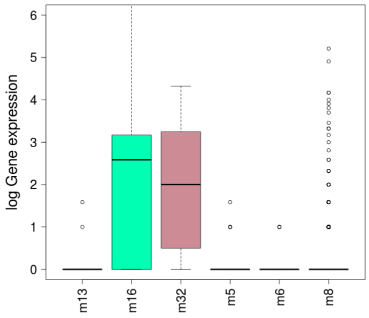

(g) Myh11 (RSEC)

Figure S2: **Boxplots of the (log) gene expression values for endothelial markers using `plotFeatureBoxplot`**. We show boxplots of the expression of the three markers used by [18] to characterize the biological function of clusters "Endo1/Epith1" and "Endo2/Epith1": Cldn5 (Endothelial), Slc38a5 (Endo1), Myh11 (Endo2). We show both the original clusters of [18] and those of RSEC.

Cluster	# Cells Total	# Assigned by [18]	Cldn5 (Endo)	Slc38a5 (Endo1)	Myh11 (Endo2)	% Endo1	% Endo2	
m5	373	342	✓	✗	✗	86	14	
m6	342	323	✓	(*)	✗	94	6	
m8	335	317	✓	✗	✗	55	45	olfactory
m13	120	38	✗	✗	✗	0	100	
m16	260	123	✗	✗	✓	17	83	
m32	20	15	✓	✗	✓	7	93	
Endo1	818		✓	(*)	✗	100	0	
Endo2	379		✓	✗	(*)	0	100	

Table S1: **Summary of expression Endothelial markers of [18]**. ✓corresponds to the gene being well expressed, ✗ corresponds to non-expressed, and (*) corresponds to a mixture of expressed and non-expressed. The percentage in Endo1/Endo2 are based only on those cells classified by [18]. See Fig S2 for more detail.

References

[1] Lun A, Risso D. SingleCellExperiment: S4 Classes for Single Cell Data; 2017.

[2] Tseng GC, Wong WH. Tight clustering: a resampling-based approach for identifying stable and tight patterns in data. Biometrics. 2005;61(1):10–16.

[3] de Jong J. Faster Hamming distance in R; 2015. Available from: `https://johanndejong.wordpress.com/2015/10/02/faster-hamming-distance-in-r-2/` [cited October 9, 2017].

[4] Smyth GK. Limma: linear models for microarray data. In: Gentleman R, Carey V, Dudoit S, R Irizarry WH, editors. Bioinformatics and Computational Biology Solutions using R and Bioconductor. New York: Springer; 2005. p. 397–420.

[5] Law CW, Chen Y, Shi W, Smyth GK. voom: Precision weights unlock linear model analysis tools for RNA-seq read counts. Genome Biology. 2014;15(2):R29.

[6] Robinson MD, Mccarthy DJ, Smyth GK. edgeR: a Bioconductor package for differential expression analysis of digital gene expression data. Bioinformatics (Oxford, England). 2010;26(1):139–140.

[7] Van den Berge K, Perraudeau F, Soneson C, Love MI, Risso D, Vert JP, et al. Observation Weights to Unlock Bulk Rna-Seq Tools for Zero Inflation and Single-Cell Applications. Genome Biology. 2018;19(24).

[8] Storey J. A Direct Approach to False Discovery Rates. Journal of the Royal Statistical Society Series B (Statistical Methodology). 2002;64(3):479–498.

[9] Pounds S, Cheng C. Improving false discovery rate estimation. Bioinformatics (Oxford, England). 2004;20(11):1737–1745.

[10] Jin, Jiashun, Cai, T Tony. Estimating the Null and the Proportion of Nonnull Effects in Large-Scale Multiple Comparisons. Journal of the American Statistical Association. 2007;102(478):495–506.

[11] Ji J;. Available from: `http://www.stat.cmu.edu/~jiashun/Research/software/NullandProp/` [cited December 16, 2015].

[12] Efron B. Large-Scale Simultaneous Hypothesis Testing: The Choice of a Null Hypothesis. Journal of the American Statistical Association. 2004;99(465):96–104.

[13] Meinshausen N, Buhlmann P. Lower bounds for the number of false null hypotheses for multiple testing of associations under general dependence structures. Biometrika. 2005;92(4):893–907.

[14] Fletcher RB, Das D, Gadye L, Street KN, Baudhuin A, Wagner A, et al. Deconstructing Olfactory Stem Cell Trajectories at Single-Cell Resolution. Cell stem cell. 2017;20(6):817–830.e8.

[15] Cole MB, Risso D, Wagner A, DeTomaso D, Ngai J, Purdom E, et al. Performance Assessment and Selection of Normalization Procedures for Single-Cell RNA-Seq. bioRxiv. 2017; p. 235382.

[16] Lun ATL, McCarthy DJ, Marioni JC. A step-by-step workflow for low-level analysis of single-cell RNA-seq data with Bioconductor. F1000Res. 2016;5:2122.

[17] Csardi G, Nepusz T. The igraph software package for complex network research. InterJournal. 2006;Complex Systems:1695.

[18] Chen R, Wu X, Jiang L, Zhang Y. Single-Cell RNA-Seq Reveals Hypothalamic Cell Diversity. Cell Reports. 2017;18(13):3227 – 3241. doi:https://doi.org/10.1016/j.celrep.2017.03.004.

[19] at the Sanger Institute HG. scRNA-Seq Datasets; 2018. Available from: `https://hemberg-lab.github.io/scRNA.seq.datasets/`.

[20] Kodama T, Guerrero S, Shin M, Moghadam S, Faulstich M, du Lac S. Neuronal Classification and Marker Gene Identification via Single-Cell Expression Profiling of Brainstem Vestibular Neurons Subserving Cerebellar Learning. Journal of Neuroscience. 2012;32(23):7819–7831.
